# Chronic activation of the epithelial immune system of the fruit fly's salivary glands has a negative effect on organismal growth and induces a peculiar set of target genes

**DOI:** 10.1186/1471-2164-11-265

**Published:** 2010-04-26

**Authors:** Ahmed Abdelsadik, Thomas Roeder

**Affiliations:** 1 Christian-Albrechts-University of Kiel, Zoophysiology, Olshausenstraße 40 D-24098 Kiel, Germany; 2 South Valley University of Aswan, Zoology Department, Faculty of Science at Aswan, Aswan Branch, 81528 Aswan, Egypt; 3 Forschungszentrum Borstel, Dept. Immunology and Cell Biology, Parkallee 1-40, 23845 Borstel, Germany

## Abstract

**Background:**

Epithelial and especially mucosal immunity represents the first line of defence against the plethora of potential pathogens trying to invade via the gastrointestinal tract. The salivary glands of the fruit fly are an indispensable part of the gastrointestinal tract, but their contribution to the mucosal immunity has almost completely been neglected. Our major goal was to elucidate if the fly's salivary glands are able to mount an immune response and what the major characteristics of this immune response are.

**Results:**

Ectopic activation of the IMD-pathway within the salivary gland cells is able to induce an immune response, indicating that the salivary glands are indeed immune competent. This reaction is characterized by the concurrent expression of numerous antimicrobial peptide genes. In addition, ectopic activation of the salivary gland's immune response induces morphological changes such as dwarfism throughout all developmental stages and a significantly decreased length of the salivary glands themselves. DNA-microarray analyses of the reaction revealed a complex pattern of up- and downregulated genes. Gene ontology analyses of regulated genes revealed a significant increase in genes associated with ribosomal and proteasomal function. On the other hand, genes coding for peptide receptors and some potassium channels are downregulated. In addition, the comparison of the transcriptional events induced following IMD-activation in the trachea and the salivary glands shows also only a small overlap, indicating that the general IMD-activated core transcriptome is rather small and that the tissue specific component of this response is dominating. Among the regulated genes, those that code for signaling associated protease activity are significantly modulated.

**Conclusions:**

The salivary glands are immune-competent and they contribute to the overall intestinal immune system. Although they produce antimicrobial peptides, their overall response is highly tissue-specific. Our analysis indicates that chronic activation of the salivary gland's immune system is costly, as it induces severe reduction in growth throughout development. The IMD-regulated increase in expression levels of the fly's presenilin representatives opens the opportunity to use the salivary glands for studying the physiological and pathophysiological role of these genes in a simple but functional environment.

## Background

The immune system is indispensable for multicellular organisms to cope with different pathogens such as bacteria, viruses or fungi. Most animals solely rely on their innate immune system that utilizes phagocytosis, encapsulation, or antimicrobial peptides among many other strategies[1,2]. Even in vertebrates, where it functions as a first line of defence, it is indispensable for normal life. Interest in innate immunity was revitalized by the identification of Toll-receptors in *Drosophila *[3] and since then the fly has served as one of its most informative models [4]. The phylogenetically most ancient type of innate immune systems, the epithelial immunity, has almost completely been overlooked. Epithelial cells, including those of the airways and the intestine, as the most important ones, are able to launch a protective, antimicrobial response if confronted with pathogens. Most notably, both epithelia react with expression of antimicrobial peptide genes [5,6]. The intestinal immune system is in a special position, because it not only has to fight pathogens, but at the same time, it has to protect the endogenous microbiota. Thus, holding a homeostatic balance between tolerance and profound immune responses is the most important regulatory task of the intestinal immune system. Deregulation of this homeostatic equilibrium might end up into chronic inflammatory diseases of the epithelium, such as Crohn's disease and ulcerative colitis. In *Drosophila*, a wealth of information regarding the architecture of innate immune systems has accumulated over the last decade. Although most studies focussed on the systemic immune response, some information regarding epithelial immunity, especially about intestinal immunity were generated. In *Drosophila*, only two signalling pathways converge onto activation of NF-κB factors, the Toll- and the IMD-pathway [4]. Now, it is common knowledge that epithelial immune responses in the fly rely on the IMD-pathway. We have shown that, at least in the airway epithelium, the inability of the Toll-pathway to function properly, is due to the lack of vital pathway members [7]. Nevertheless, it has to be mentioned that other pathways, not directly converging onto NF-κB activation, such as the JNK- or the JAK/STAT-pathway are operative in epithelial immunity [8,9].

In the intestinal epithelium, two major response types have been reported following pathogen challenge. These reactions are the release of antimicrobial peptides and the production of reactive oxygen species [10-13]. The intestine's function in *Drosophila *is to digest microbe-rich food such as decaying fruits while protecting the fly from infections. Expression of AMPs is enhanced by activation of the IMD pathway and provides a partial protection against entomopathogenic bacteria such as *Pseudomonas entomophila*[14]. Another arm of the immune response that complements the IMD response of AMP genes is the production of reactive oxygen species (ROS). Apparently, this system is sufficient to cope with most pathogens[10,11]. IMD-mediated response of AMP-genes seems to be a back up system to control ROS-response against pathogens[12,13].

In general, the intestinal epithelium appears to be refractory towards pathogen confrontation, presumably due to the presence of inhibitory signals, either those that digest peptidoglycans as the most important pathogen associated molecular patterns (PAMPs) or those that repress IMD-mediated expression[15,16]. Taken together, regulation of antimicrobial responses in the intestinal epithelium appears to be a complex network including activation and inhibition at different levels [17-19].

Another part of the digestive system that may have the potential to mount an immune response has largely been overlooked, the salivary glands. Salivary glands are an integral part of the intestinal tract, often enabling the uptake and transport of ingested material into the digestive tract. They are the gatekeepers of the intestinal system, being essential for various aspects of the intestine's immunity. *Drosophila *salivary glands consist of two major cell types: secretory cells that synthesize and secrete high levels of protein and duct cells that form the simple tubes connecting the secretory cells to the larval mouth. Salivary glands differentiate without further cell division and increase in size simply by increasing the volume of individual cells. In recent studies, salivary glands served as a model to study aspects of relevant for organogenesis and the role of various different genes in this process [20-22]. Here we show that salivary glands can mount a protective immune response that is characterized by the expression of antimicrobial peptide genes. Using ectopic expression of the *pgrp-l*c gene, a procedure, which has already been shown to activate innate immune responses in the fly [23,24], we specifically activate the immune response of the salivary glands. Fly larvae experiencing a sustained activation of their salivary gland IMD-pathway show a pronounced phenotype that is characterized by reduced overall size of the organ and reduced size of the flies. In addition to various antimicrobial peptide genes, expression of numerous other genes with relevance for signalling, including proteases with signalling activities such as presenilin or signal peptide peptidase is regulated in this organ.

## Results

**Figure 1:**
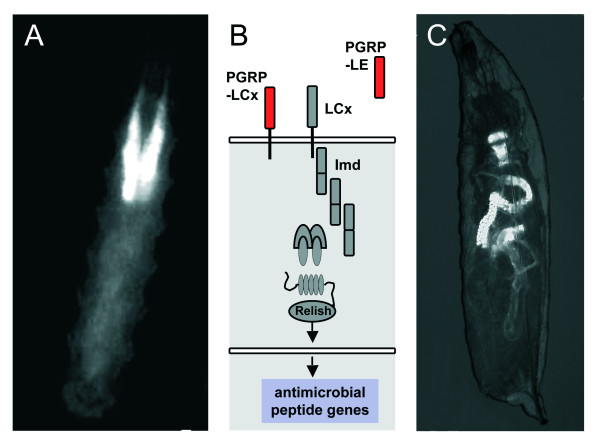
**Using the Gal4/UAS-system, activation of the salivary glands can be achieved by genetic means**. Proteins of the salivary gland secretion family are believed to be restricted to the salivary glands. Crossing the sgs3::gal4 line with a UAS::gfp responder line revealed that expression is indeed restricted to the salivary glands (A). Based on the UAS/Gal4 system for ectopic activation, the IMD-pathway, which is instrumental in epithelial immunity, can be activated by targeted overexpression of peptidoglycan recognition receptors. The membrane bound PGRP-LC activates in a cell-autonomous way, whereas the soluble PGRP-LE activates in an organ-wide, systemic way (B). If the *pgrp-le *gene is expressed in the salivary glands only, the antimicrobial peptide gene *drosomycin *(here used as a reporter for immune system activity and visualized by *drosP*::gfp) is expressed in defined parts of the intestine only (but not on the salivary glands) (C).

A major prerequisite for the current study was the ability to specifically target the salivary glands of larval flies without any confounding expression. This is required to manipulate expression of certain genes within this tissue only. Salivary gland secretion (sgs) proteins are believed to be restricted to salivary glands. To evaluate if this specific expression pattern is operational for the corresponding Gal4-lines, we used the bipartite Gal4/UAS expression control system and crossed the Gal4-driver P [Sgs3-GAL4.PD]TP1 to UAS-flies carrying *gfp *under control of the UAS-promotor. The expression of the GFP-protein is, as expected, exclusive for the salivary glands (Fig. 1A).

Targeted expression of PGRPs was shown to be sufficient for activation of the IMD-pathway, one major arm of the fly's innate immune system. It enables immune activation without any contact to pathogens in a spatially restricted manner, in those tissues that are immune competent. We used two different effector lines, enabling either production of the membrane protein PGRP-LC or of the soluble receptor PGRP-LE. Ectopic production of PGRP-LC is expected to induce a cell-autonomous immune response, whereas that of PGRP-LE is expected to induce an organ-wide or even systemic response (Fig. 1B). In initial experiments, it became apparent that ectopic expression of the *pgrp-le *gene in the salivary glands only induces activation also in other parts of the intestinal system (Fig. 1C), indicating that it acts as an organ-wide activator. In contrast, the primary effects of *pgrp-lc *expression remain restricted to the salivary glands. All following experiments were performed with the *pgrp-lc *construct allowing to focus on the effects of IMD-pathway activation in the salivary glands only.

**Figure 2:**
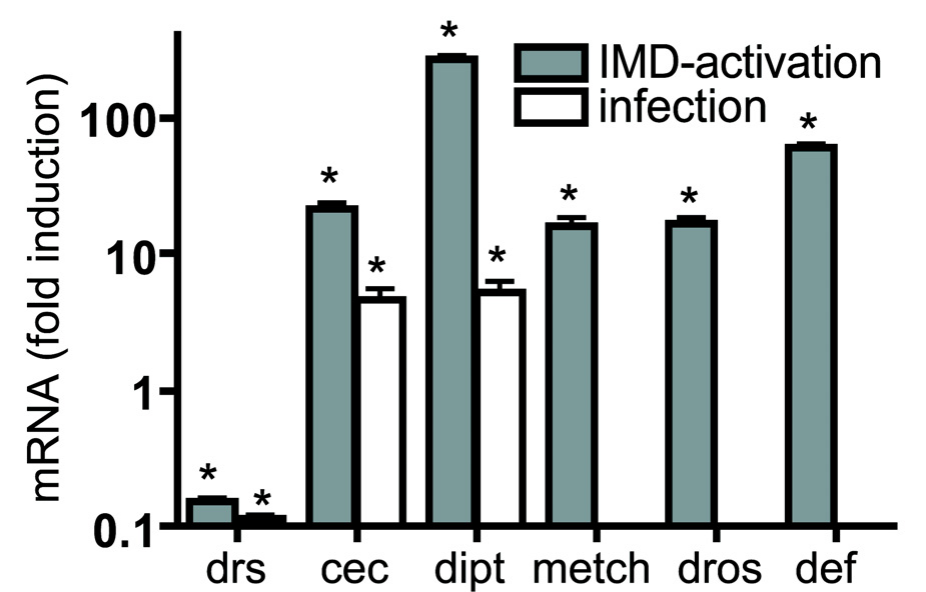
**Infection and ectopic activation of the IMD-pathway induces expression of antimicrobial peptides in the salivary glands**. Oral infection of early 3^rd ^instar with the insect pathogen *Erwinia carotovora *was used to monitor transcriptional changes in 3 selected antimicrobial peptide genes, drosomycin (drs), cecropin (cec) and diptericin (dipt)(white bars). Ectopic overexpression of the *prgp-lc *gene in the larval salivary glands was achieved using the Gal4/UAS system (sgs3::gal4 X UAS::pgrp-lc). Salivary glands of early 3^rd ^instar larvae were isolated from these crossing and a parental line (responder line) used for control. Quantitative real-time PCR was performed with oligonucleotides comprising approximately 150 bp of the corresponding antimicrobial peptide genes; drosomycin (drs), cecropin (cec), diptericin (dipt), metchnikowin (metch), drosomycin (dros) and defensin (def) (grey bars). Results are the mean of at least 3 experiments performed in triplicate. Controls are set to 1. Statistically significant differences (compared with controls are marked by an asterik (p < 0.05).

To evaluate if the salivary glands of larval *Drosophila *are able to react to an immune challenge and to characterize this reaction type, we performed two types of experiments. First, conventional oral infection experiments were used to evaluate if this epithelial tissue is able to launch a classical immune response, characterized by increased expression of antimicrobial peptide genes. Using promotor indicator lines, where *gfp *expression is under transcriptional control of antimicrobial peptide gene promoters, no response could be observed using infection with *Erwinia carotovora *(data not shown). Using these oral infection experiments with *Erwinia carotovora *and qRT-PCR, we evaluated a set of three antimicrobial peptide genes; namely, *drosomycin*, *cecropin *and *diptericin*. Whereas expression of *drosomycin *is reduced, both, *cecropin *and *diptericin *expression is increased approximately 5 fold (Fig. 1). To show that ectopic *pgrp-lc *expression is able to induce an autonomous immune response in the larval salivary glands, we isolated salivary glands of control animals (parental lines) and those of the sgs3-Gal4 X UAS-*pgrp-lc *crosses. Isolation was performed manually from early 3^rd ^larval instars and the salivary glands were thoroughly freed from attached fat body material. This material was used for quantitative real-time PCR experiments following standard procedures. We tested the expression levels of the canonical set of antimicrobial peptide genes, including *metchnikowin (metch)*, *defensin (def)*, *drosocin (dros)*, *drosomycin (drs)*, *cecropin (cec) *and *diptericin (dipt)*. Expression of all of them, except that of *drosomycin*, is upregulated between 10 and more than hundred fold (Fig. 2). Greatest changes are seen for *diptericin *and *defensin *with approx. 80 and 160 fold increases, respectively. Expression of *drosomycin *on the other hand, is instead downregulated significantly.

**Figure 3:**
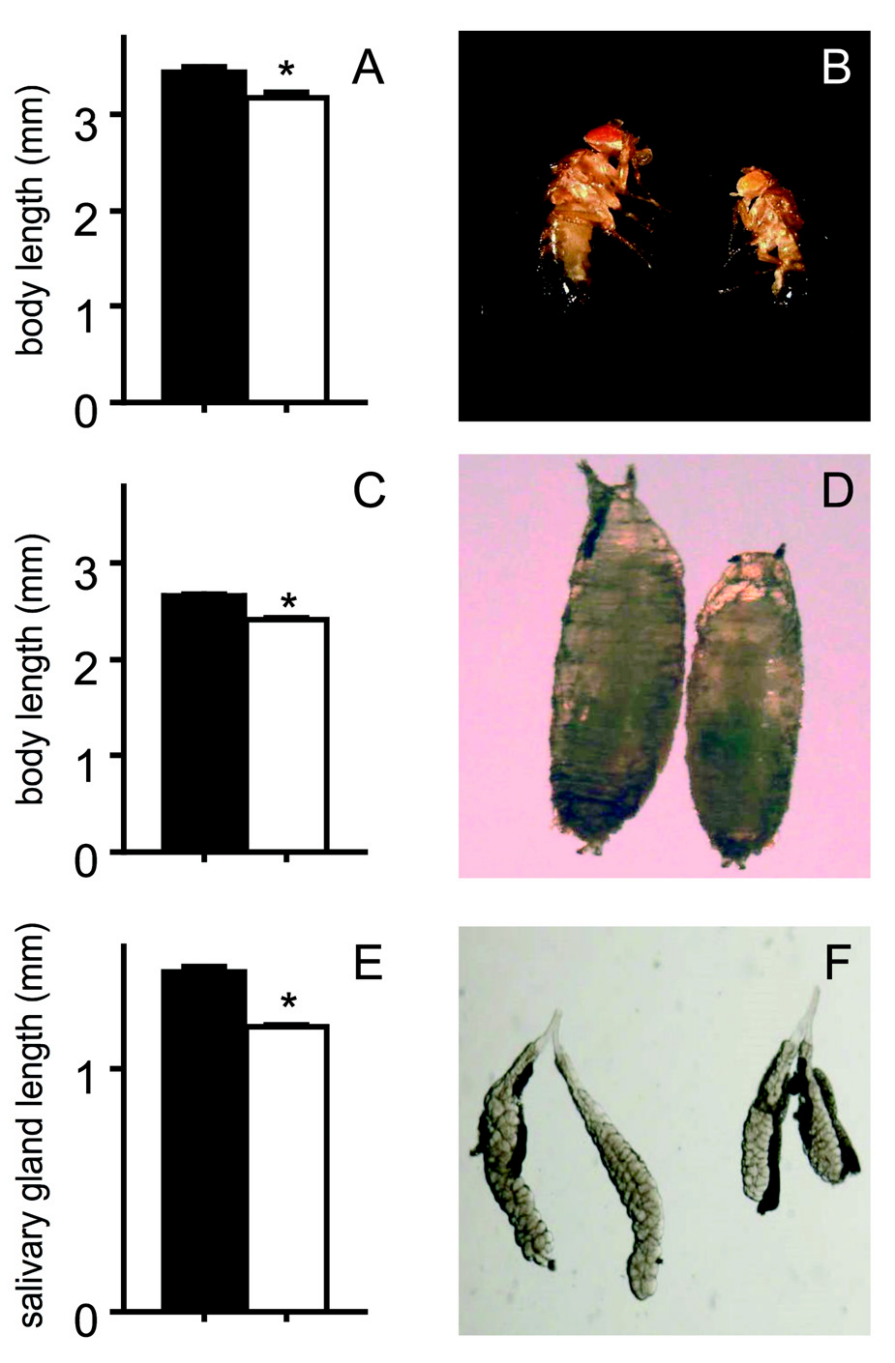
**Chronic ectopic activation of the IMD-pathway in the salivary glands only had a significant effect on growth and body length of the animals**. Adult males, where PGRP-LC is ectopically expressed in the salivary gland have an approximately 10% reduced body length if compared with the parental line (A, B). A similar reduction can be seen in the pupal length of both types of animals (C, D). Regarding the lengths of isolated salivary glands from the corresponding animals, these differences are even more pronounced (E, F). For A, C, E, median values of at least 20 measurements +/- SEM. Statistically significant differences are marked by an asterik (p < 0.05).

Flies, where we activated the immune response in the salivary glands show a distinctive phenotype, they are much smaller than their relatives from the parental lines. Adults derived from the crosses are approximately 10% smaller than their relatives of the parental lines that were kept under identical conditions (Fig. 3A, B). This difference in size is not restricted to adults, but it can also be seen in pupae (Fig. 3C, D) and wandering 3^rd ^instars respectively (controls: 954 μm ± 45.6 μm; experimental animals: 696 μm ± 68 μm; difference is statistically significant - p < 0.05). Here, also differences of about 15% are conspicuous (Fig. 3C, D). A very similar decrease in length is apparent for the salivary glands themselves, which are more than 20% shorter than those isolated from the parental lines (Fig. 3E, F).

**Figure 4:**
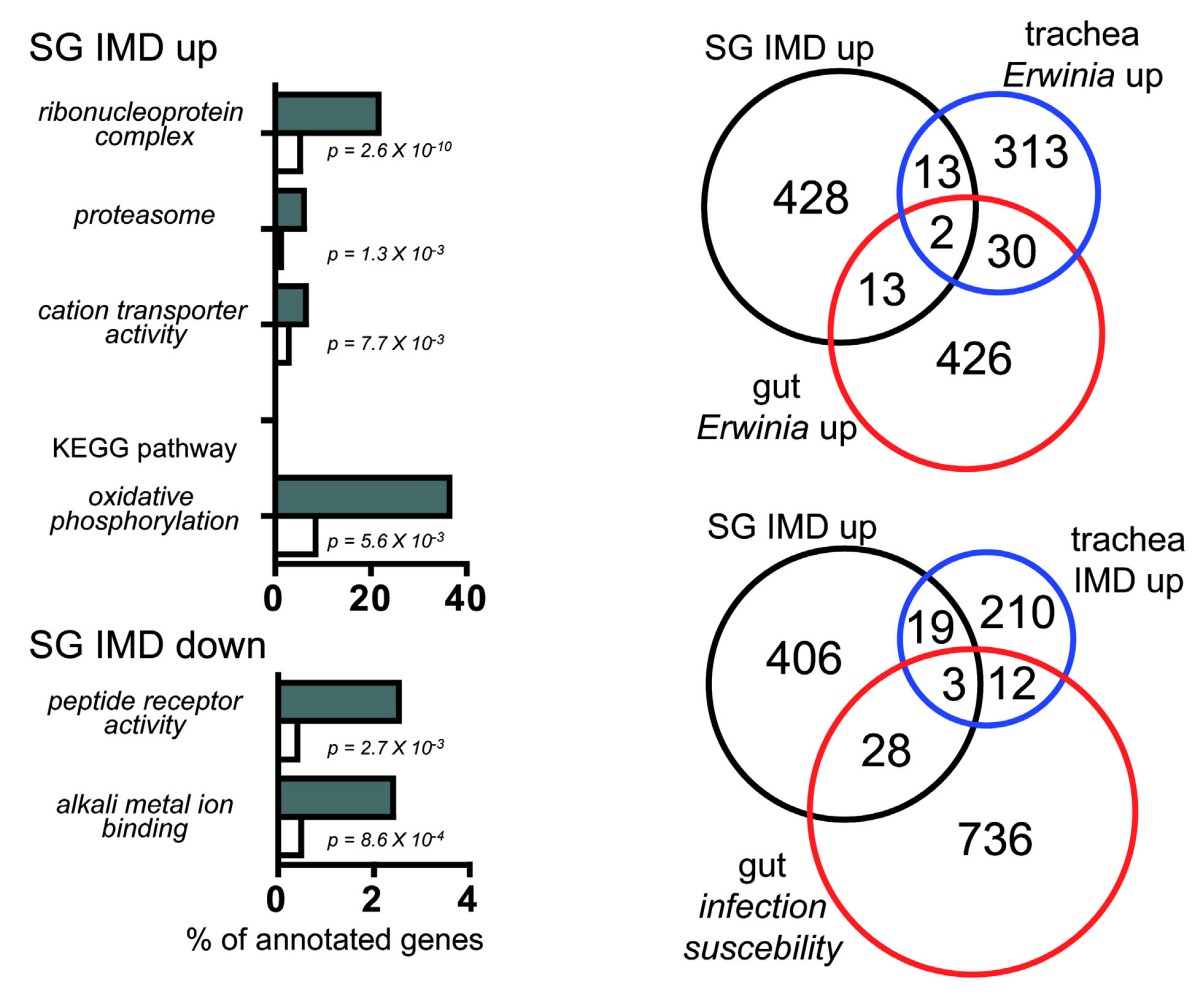
**DNA-microarray analyses of the transcriptional changes within the salivary glands that occur following chronic activation of the IMD-pathway in these cells only**. Cohorts of genes were identified that are either upregulated (additional file 3) or downregulated (additional file 4). Only those genes are included, where these differences are greater than twofold and the differences are statistically significant (evaluated with the SAM software package). Gene ontology analyses were performed with these cohorts of genes and compared to the complete set of *Drosophila *genes using the Fatigo software tool [25] revealed some statistically significant differences (A, top). Comparison of the set of upregulated genes with the complete set of *Drosophila *genes revealed some groups of genes that are highly enriched in this cohort (top). In addition, KEGG pathway analysis identified the oxidative phosphorylation as being significantly enriched in this cohort of genes. The cohort of downregulated genes includes two functional groups, being significantly different from the total number of genes (A, bottom). The corresponding lists are summarized in additional file 5. Venn diagram analyses showed that only a small minority of the up- or downregulated genes are classical immune relevant genes (additional file 1). Similarly, the intersection with those genes that are regulated upon an experimental infection with the insect pathogen *Erwinia carotovora *in two different epithelial tissues are rather small (B). In addition, small groups of genes are identical if compared with genes upregulated in the trachea following the same experimental manipulation (IMD-activation) and with susceptibility genes for gut infection (C), SG IMD up means the cohort of genes upregulated following IMD activation (see additional files 1, 2, 3, 4 and 5), gut and trachea *Erwinia *up means the groups of genes upregulated in the trachea or the gut following infection with *Erwinia carotovora *[9,27], trachea IMD up means the list of genes upregulated following IMD activation in the larval trachea [27], gut infection susceptibility means those genes that have been identified as having an influence on oral infection in a genome wide RNAi screen [8].

An unbiased study of the effects mediated by ectopic IMD-pathway activation in the larval salivary glands was performed using DNA-microarray analyses. Therefore, we isolated the salivary glands exactly as described above, amplified and labeled the material and hybridized it onto microarrays covering most known genes of the fly. If the salivary gland transcriptomes of control larvae and those of larvae that experienced a chronic IMD-pathway upregulation that was restricted to the salivary glands, significant differences can be observed. A total of 458 genes are significantly upregulated and 579 genes are significantly downregulated (Fig. 4).

Gene ontology analysis using the Fatigo program package [25] revealed significant peculiarties of both, the up- and downregulated genes in the larval salivary glands following activation of the IMD-pathway (Fig. 4A). GO ontology analysis in comparison with all *Drosophila *genes revealed that some functional groups are significantly enriched in the cohort of upregulated genes. Most significantly is the clustering of gene products associated with ribonucleoprotein complexes, the proteasome and with cation transporter activities. KEGG pathway analysis of these genes revealed a significant enrichment of the oxidative phosphorylation pathway (Fig. 4A, top, additional file 1). The cohort of downregulated genes shows a clustering of genes associated with peptide receptor activity and alkali metal ion binding (Fig 4A, bottom, additional file 1). 24 ribosomal proteins in total are among the upregulated genes in the salivary glands and 7 genes coding for proteasome subunits. Regarding the downregulated genes, peptide receptor coding genes are conspicuous. In addition to allatostatin receptors, a tachykinin-like, a SIFamide and a gonadotropin-releasing hormone receptor are downregulated (additional file 1). In the group of alkali metal ion binding, ion channels, especially potassium channels are present (such as shaker b and ether a go-go).

A more detailed analysis of cohorts of regulated genes revealed only a minor overlap with the canonical set of immune genes set up in the Lemaitre lab http://www.cnrs-gif.fr/cgm/immunity/index_gb.html (Fig. 4B, C). This comprehensive list is a compilation of genes identified in whole genome wide infection studies [26] and bioinformatics analysis of *Drosophila *genes. Only 11 genes in the upregulated and 18 genes in the downregulated cohort are classified as immune genes, meaning that just 2-5% of all regulated genes fall into this category. Based on Venn-diagram analysis, we compared the cohort of upregulated genes with those upregulated following an experimental infection with the insect pathogen *Erwinia carotovora *either in the trachea or the gut [9,27](Fig. 4B). Overlaps are also not high with 15 genes in common between Salivary glands upregulated and either the tracheal or the gut infection upregulated cohorts (Fig. 4B, additional file 2).

Using a similar experimental approach, i.e. ectopic activation of the epithelial immune system through expression of the *pgrp-lc *gene, we identified the regulated transcriptomes in the larval airway epithelium, the trachea. For these experiments, animals with the same genetic background, which are of the same age, and were held under identical conditions, were used [7]. The overlaps in the cohorts of regulated genes are slightly larger (22) (Fig. 4C). Thus, these experiments indicate that NF-kB dependent gene expression is highly context-dependent, meaning that different tissues activate largely different sets of genes if the IMD-pathway is activated ectopically. In addition, a total of 31 genes are shared with the set of genes identified as involved in susceptibility to gut infections [8] (Additional files 3 and 4. Larval salivary glands are an exquisite model for studying the molecular basis of autophagic cell death. In the experimental flies, we found no histological signs of autophagic cell death. In addition, we only found very few genes that are usually associated with autophagic cell death to be regulated (up: maf-s, rab-7. Obp-99, elf-5A, cyclophilin-1, CG10992, CG9760; down: comm3). This finding is in-line with earlier observations that NF-kB activation is not necessary for cell death of salivary glands, thus excluding confounding influences from factors related to autophagy in the salivary glands [28].

**Figure 5:**
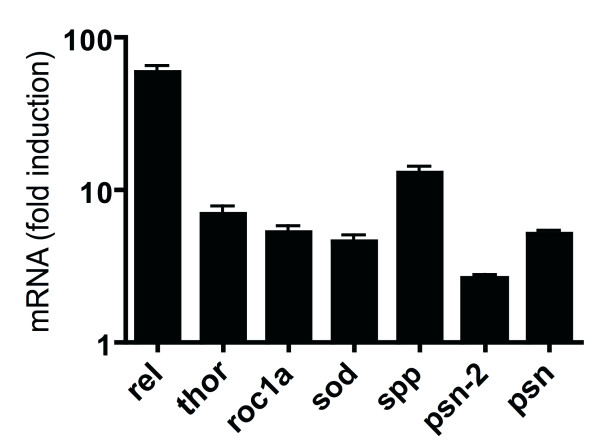
**Verification of differential expression of some highly interesting genes, which are regulated following IMD-activation within the salivary glands**. RNA is isolated from pure salivary glands (freed of contaminating material) of both experimental groups and the results are the means of at least three independent experiments performed in duplicate. All genes are transcribed at significantly higher rates in the experimental tissues compared with controls (p < 0.05).

Verification of differential expression was performed with a small subset of obviously interesting genes, that came out of the DNA-microarray studies as being regulated (Additional file 5). Among them are e.g. *sod *or *thor*, but presumably as the most important ones, those that are involved in signaling through protease activity. These are the two presenilin-like genes of *Drosophila *and the signal peptide peptidase (spp). For all these genes, we were able to verify the differential expression (Fig. 5). Experiments were performed with isolated salivary gland material from control as well as from experimental animals.

## Discussion

The salivary glands of the fly are an important part of the digestive tract and thus should contribute to the immune response of the entire organ. In addition, salivary gland function is indispensable for normal life, making it necessary to protect this organ. A partial protection is achieved through its architecture, because the salivary gland secretion moves into the intestinal system, making it hard for bacteria to overcome this constant flow. We got a first impression of the yet uncharacterized interplay between salivary glands and digestive system, while taking a look at those animals that ectopically express *pgrp-le *in the salivary glands only. This soluble protein, although produced in the salivary glands only, can migrate through the entire intestinal tract thus functioning as a general activator of the intestinal immune response. It shows that products of the salivary gland have in general the potential to modulate the intestinal immune system, highlighting its importance for intestinal immune homeostasis.

Salivary gland cells, similarly as most epithelial cells, are indeed able to launch an immune response if confronted with an orally applied pathogen. Our experiments demonstrate that two out of three antimicrobial peptide genes are expressed at significantly higher levels; namely, *cecropin (cec) *and *diptericin (dipt)*. Nevertheless, this increase is not sufficient to be visible in promotor gfp indicator strains. Conventional oral infections are hard to perform and the response characteristics are variable, making it not trivial to identify the major response characteristics of this epithelial immune response. Alternatively, the forced IMD-activation, enables a more focused view on the major characteristics of this type of immune system. Within the salivary gland cells, the response following forced IMD-pathway activation comprises increased expression of antimicrobial peptide genes, which is generally accepted to be the "normal" reaction to this type of stimulus. This clearly shows that the larval salivary gland is an immune competent epithelium, similarly as the intestinal, the airway or the subcuticular epithelium [5-7,29]. Regarding the set of AMP genes that showed an increased expression, the exceptional role of *drosomycin *(drs) is suspicious. The lack of Toll-mediated immune signaling appears to be a common feature of all epithelial immune systems characterized in the fly so far [6,29]. Drosomycin is thought to be the major Toll-pathway dependent antimicrobial peptide; a lack of *drosomycin *upregulation would thus be in-line with the absence of Toll-mediated signaling in epithelial immunity. Nevertheless, it is puzzling that in the trachea, the only epithelial tissue where a similar experimental approach has been performed, Drosomycin is the major AMP that comes up following infection or ectopic IMD-pathway activation [7]. It has to be mentioned that *drosomycin *expression is striking in adult salivary glands, which is congruent with earlier findings [6].

Chronic activation of the salivary gland's immune system comes along with costs for the animal. Reduced sizes in both, the salivary glands and of the entire organism in all its developmental stages, are the result of this activation. The reason for this remains unclear, but apparently, chronic activation impairs the function of the salivary glands, which in turn might result in a reduced capacity to take up nutrients. Therewith, this ectopic regime of epithelial immune system activation shares some commonalities with chronic inflammatory diseases of barrier epithelia [30]. Growth retardation is also seen in children suffering from inflammatory bowel diseases [30]. In these children, malnutrition is obviously not the sole reason for this growth retardation, but the production and regulation of proinflammatory cytokines may also be relevant for this phenotype [31]. Induced expression of membrane tethered proteases such as Presenilin or SPP may thus be a link, as one can speculate that they trigger signaling events while freeing signaling ligands from the membrane. Nevertheless, we have no experimental data if the phenotype inducing processes are restricted to the salivary glands or if they can be attributed to e.g. an increased antimicrobial activity in the intestinal lumen, which in turn may interfere with the microbiota. Although this possibility could not be ruled out, gnotobiotic flies do not show such a significant effect on growth parameters as the one observed in our study.

Surprisingly, only a relatively small group of genes among the cohorts of up- and downregulated ones can be assigned to the set of *Drosophila *immune genes [26]. Although expression of a complex set of AMP genes is induced, most other induced genes apparently have nothing to do with "conventional" immune responses. This obvious difference between a "conventional" immune response and that of an immune competent epithelium is not restricted to the fly's salivary glands, it can also be seen in another epithelium of the fly, the airways [7,27]. Apparently, morphological changes that result from chronic immune pathway activation may be one reason for this incongruence. Very surprising is the small overlap between the cohorts of up- or down-regulated genes between the salivary glands and those seen following a natural infection in either the gut or the airways. The same is true for the comparison between IMD-activated transcription in the salivary glands and in the airways, although both experiments were performed with a very similar design. Despite the fact that in both tissues IMD-pathway activation induces an immune response, most parts of the induced response differ substantially between both organs. This indicates that IMD-dependent signaling has only a very small core response shared by all organs and that the tissue specific response is far more important in shaping the transcriptional response following IMD-pathway activation.

A classification according to the gene ontologies revealed some very interesting aspects in both cohorts of genes (up- and downregulated). Among the upregulated genes, both, the ribosome associated as well as the proteasome associated proteins are significantly enriched. Functionally, this would mean that both, the translational capacity and the targeted degradation of proteins intended to proteasomal degradation, are enhanced. KEGG pathway analysis revealed only the oxidative phosphorylation as significantly enriched pathway, which we currently do not understand. Among the cohort of downregulated genes, peptide receptors are conspicuous. A total of six peptide receptors, including all allatostatin receptors [32] are downregulated. In before, it has not been known that this high number of different neuropeptides have the ability to modulate the activity of salivary glands, which is up to now ascribed to biogenic amines only [33]. Some of the genes that are not part of a conspicuous gene ontology are nevertheless of great interest. Intramembrane proteolysis is thought to be a highly relevant way in cell signaling, which can be fulfilled by only a very small number of proteases. Among them, the presenilin-like proteases are of prime importance. Presenilin proteases gained substantial interest following identification of presenilin mutants being causative for some forms of familial Alzheimer disease [34]. They are part of the gamma-secretase complex, thus having a central role in the formation of amyloid plaques [35]. This concerted activation of the proteases highly relevant for countless signaling systems, may be one major reason for the different transcriptomic responses that can be seen both in the trachea and the salivary gland following identical experimental stimulation protocols. Their role in the immune response of this epithelium is completely enigmatic. Nevertheless, these results offer a unique opportunity to use this natural system to study the physiological significance of them in this extremely simplified model system. The fly's salivary glands are of very simple organization, made of only two cell types. In addition, high amounts of pure material can be generated and the organ is highly amenable to specific genetic interventions of different types including RNAi-mediated gene silencing or specific and timed overexpression.

Two other genes, which are known to be associated with stress or immune responses are also upregulated in this organ, the *superoxide dismutase *(SOD) and the integrator of stress responses *dThor*. In the intestinal epithelium, the redox homeostasis appears to be one major way to fight infections while protecting the endogenous microbiota. In this context, reactive oxygen species generated by the dual oxidase Duox are apparently of prime importance. In the salivary gland, only the SOD appears to be upregulated, pointing to a role in defence of the own epithelium in response to a real or to an anticipated infection.

## Conclusions

Salivary glands are an integral part of the gastro-intestinal tract. In the fruit fly, they are indispensable for efficient food intake. As they are blind ending tubular organs, they are particularly prone to infections. We were able to show that the fly's salivary glands are able to mount an immune response if they are confronted with pathogens. This response primarily depends on the production of antimicrobial peptides. To study this response in detail, we established an ectopic expression control system that allows activation the epithelial immune system of the salivary glands without pathogen contact. Although they produce antimicrobial peptides, their overall response appears to be specific for the salivary glands. Chronic activation of the salivary gland's immune system is costly, as it induces severe reduction in growth throughout development. This negative effect could be seen in all developmental stages. Surprisingly, a number of different genes are regulated upon ectopic activation of the IMD-pathway. Among them are proteins involved in presenelin function. Studying this molecule, which is central to Alzheimer pathology, may allow us to unravel some of the aspects of presenelin function in a simple but functional environment.

## Methods

### Fly strains and experimental manipulation

For the ectopic expression experiments, we used the Gal4 driver line P [Sgs3-GAL4.PD]TP1, which directs expression into the salivary glands of larvae and adults only. Ectopic activation of the immune system was achieved by crossing these flies to either UAS-*pgrp-le *or UAS-*pgrp-lc *comprising flies [24]. *UAS-pgrp-le *containing flies also carry a *drsP::gfp *cassette (flies were generously provided by KV Andersen and S Kurata). For control experiments, we generally used the responder line (mainly UAS-*pgrp-lc*). Flies were raised on standard cornmeal-agar medium at 20°C.

### Infection experiments

For infection experiments, early 3^rd ^instar larvae were confronted with the gram-negative bacterium *Erwinia carotovora *according to protocols used in oral infection experiments. Overnight cultures of the bacterium were centrifuged, concentrated (OD_600 _= 100) and mixed with banana at a ratio of 1:1. Larvae were allowed to feed for 6 h on these bacteria, subsequently, they were allowed to feed for another 6 h on normal medium and the salivary glands were prepared out of the animals and used for further experiments. Using this material, we performed qRT-PCR analyses for *drosomycin*, *cecropin *and *diptericin *(at least using 3 independent experiments).

### Molecular biology

Manually extraction for the salivary glands of the 3^rd ^larvae instars followed by total RNA extracted using R*NAmagic solution *from (Bio-Budget technologies GmbH, Krefeld, Germany). cDNA-synthesis was performed using CapFinder as described[36]. Amplification of the cDNA was performed for 26 cycles taking advantage of a long and accurate PCR system. The integrity and quality of the amplificate was checked by gel electrophoresis. This material was used for RT-PCR experiments, qRT-PCR experiments and the production of labeled hybridization probes for DNA-microarray analysis. cDNA used for downstream applications shouldn't have any contamination of the fat bodies and thus was used only, if amplification with oligonucleotides matching the fat body specific gene P6 was not successful. Quantitative RT-PCR was performed with a Lightcycler (Roche Diagnostics, Ingelheim, Germany) using the Takara SYBR Green Real-time PCR kit (Takara Bio Inc, Japan). Probe sets were normalized against the housekeeping gene Rpl 32. At least three independent experiments were performed in duplicate.

Microarray experiments were essentially performed as described before[7]. In brief, equal amounts of amplified and purified cDNA were subjected to T7-based cRNA synthesis. The synthesis was performed with the T7 MEGAscript kit (Ambion, Applera, Darmstadt, Germany) supplemented with aaUTPs (Ambion, Applera, Darmstadt, Germany) for the labeling of the cRNA. Following purification, approximately 10 μg of aminoallyl modified-cRNA was coupled to succinimidyl modified-Cy3 und -Cy5 dyes (GE Healthcare, Freiburg, Germany) in the presence of 50% DMSO and 0.05 M NaHCO_3 _(pH 9.0) [37]. Coupling reaction was carried out for two hours in the dark followed by purification and precipitation of the labeled cRNA. After assessment of the labeled cRNA approximately 2-3 μg (or 150pmol) of Cy3 and Cy5 labeled probe were used for hybridization. Hybridisation was carried out at 42°C overnight. After hybridization, slides were washed twice with 1 × SSC, 0.1% Triton-X-100 at 60°C for 15 minutes and with 0.1 × SSC, 0.1% Triton-X-100 at 37°C for 15 min. Subsequently, they were washed with 0.1 × SSC for 30 seconds at room temperature and rinsed with water before dried.

### Microarray analysis and bioinformatics

Gene expression analysis was performed using the *Drosophila *OLIGO 14k_version1 gene chip (Canadian Drosophila Microarray Center, University of Toronto, Canada). The slides were scanned with the GenePixTM4000B scanner (Axon Instruments, Molecular Devices, Munich, Germany). For spot finding and generating preliminary result files the raw scanned image files were analyzed using GenePixPro version 6.0 whereas data normalization, quality assurance and control, filtering and clustering were carried out with GeneTraffic (Iobion, Agilent, Waldbronn, Germany) and statistical analysis with the SAM-program package. In search of functional composition of genes significantly affected upon infection and ectopic expression we used the bioinformatics web tool FatiGO[25].

The DNA-microarray experiments have been deposited in the GEO-database (accession number: GSE20938).

## Authors' contributions

AA performed the experiments, TR conceived the study and participated in its design and coordination and drafted the manuscript. Both authors read and approved the final manuscript.

## Supplementary Material

Additional file 1 **Venn diagram analysis of Drosophila Salivary glands genes upregulated following IMD-pathway activation compared with various sets of genes**. This file contains lists of those genes from Venn-diagram analyses of genes upregulated in the salivary glands following IMD-activation with various other sets of genes, identified in other experiments. Click here for file

Additional file 2 **Venn diagram analysis of Drosophila Salivary glands genes upregulated following IMD-pathway activation with various gene lists involved in reaction to infection**. This file contains lists of those genes from Venn-diagram analyses of genes upregulated in the salivary glands following IMD-activation with various other sets of genes derived from infection experiments of different tissues. Click here for file

Additional file 3 **Drosophila Salivary glands: Genes upregulated following IMD-pathway activation**. This file contains a list of those genes whose expression in the salivary glands is upregulated significantly following activation of the IMD-pathway. Click here for file

Additional file 4 **Drosophila Salivary glands: Genes downregulated following IMD-pathway activation**. This file contains a list of those genes whose expression in the salivary glands is downregulated significantly following activation of the IMD-pathway. Click here for file

Additional file 5 **Drosophila Salivary glands genes upregulated following IMD-pathway activation - ribonucleoprotein complex**. This file contains a list of those genes whose expression in the salivary glands is upregulated significantly. It comprises only those genes with an annotation as ribonucleoprotein complex. Click here for file
