# Supplementary material for: Chronic activation of the epithelial immune system of the fruit fly's salivary glands has a negative effect on organismal growth and induces a peculiar set of target genes
Source: BMC Genomics. 2010 Apr 26;11:265. doi: 10.1186/1471-2164-11-265 (PMC2874812; doi:10.1186/1471-2164-11-265)
Supplement: Additional file 1 — Venn diagram analysis of Drosophila Salivary glands genes upregulated following IMD-pathway activation compared with various sets of genes. This file contains lists of those genes from Venn-diagram analyses of genes upregulated in the salivary glands following IMD-activation with various other sets of genes, identified in other experiments. [file 1471-2164-11-265-S1.DOC]

**Common genes: Drosophila Sal Gl IMD upregulated vs canonical set of Drosophila immune genes**

| **SUBMITTED ID** | **NAME** | **SYMBOL** |
| --- | --- | --- |
| [CG10520](http://flybase.org/cgi-bin/fbidq.html?FBgn0003882) | tube | [tub](http://flybase.org/cgi-bin/fbidq.html?FBgn0003882) |
| [CG10640](http://flybase.org/cgi-bin/fbidq.html?FBgn0035601) | Uev1A | [Uev1A](http://flybase.org/cgi-bin/fbidq.html?FBgn0035601) |
| [CG18319](http://flybase.org/cgi-bin/fbidq.html?FBgn0000173) | bendless | [ben](http://flybase.org/cgi-bin/fbidq.html?FBgn0000173) |
| [CG2217](http://flybase.org/cgi-bin/fbidq.html?FBgn0027544) | - | [CG2217](http://flybase.org/cgi-bin/fbidq.html?FBgn0027544) |
| [CG32282](http://flybase.org/cgi-bin/fbidq.html?FBgn0052282) | drosomycin-4 | [dro4](http://flybase.org/cgi-bin/fbidq.html?FBgn0052282) |
| [CG4665](http://flybase.org/cgi-bin/fbidq.html?FBgn0035964) | Dihydropteridine reductase | [Dhpr](http://flybase.org/cgi-bin/fbidq.html?FBgn0035964) |
| [CG5118](http://flybase.org/cgi-bin/fbidq.html?FBgn0031317) | - | [CG5118](http://flybase.org/cgi-bin/fbidq.html?FBgn0031317) |
| [CG5773](http://flybase.org/cgi-bin/fbidq.html?FBgn0034290) | - | [CG5773](http://flybase.org/cgi-bin/fbidq.html?FBgn0034290) |
| [CG6202](http://flybase.org/cgi-bin/fbidq.html?FBgn0019925) | Surfeit 4 | [Surf4](http://flybase.org/cgi-bin/fbidq.html?FBgn0019925) |
| [CG8846](http://flybase.org/cgi-bin/fbidq.html?FBgn0022073) | Thor | [Thor](http://flybase.org/cgi-bin/fbidq.html?FBgn0022073) |
| [CG9080](http://flybase.org/cgi-bin/fbidq.html?FBgn0033593) | - | [CG9080](http://flybase.org/cgi-bin/fbidq.html?FBgn0033593) |

**Common genes: Drosophila Sal Gl IMD downregulated vs canonical set of Drosophila immune genes**

| **SUBMITTED ID** | **NAME** | **SYMBOL** |
| --- | --- | --- |
| [CG1165](http://flybase.org/cgi-bin/fbidq.html?FBgn0004430) | Lysozyme S | [LysS](http://flybase.org/cgi-bin/fbidq.html?FBgn0004430) |
| [CG4472](http://flybase.org/cgi-bin/fbidq.html?FBgn0020416) | Imaginal disc growth factor 1 | [Idgf1](http://flybase.org/cgi-bin/fbidq.html?FBgn0020416) |
| [CG5493](http://flybase.org/cgi-bin/fbidq.html?FBgn0034364) | - | [CG5493](http://flybase.org/cgi-bin/fbidq.html?FBgn0034364) |
| [CG6127](http://flybase.org/cgi-bin/fbidq.html?FBgn0004197) | Serrate | [Ser](http://flybase.org/cgi-bin/fbidq.html?FBgn0004197) |
| [CG7586](http://flybase.org/cgi-bin/fbidq.html?FBgn0020240) | Macroglobulin complement-related | [Mcr](http://flybase.org/cgi-bin/fbidq.html?FBgn0020240) |
| [CG7602](http://flybase.org/cgi-bin/fbidq.html?FBgn0037554) | DNApol-iota | [DNApol-iota](http://flybase.org/cgi-bin/fbidq.html?FBgn0037554) |
| [CG8193](http://flybase.org/cgi-bin/fbidq.html?FBgn0033367) | - | [CG8193](http://flybase.org/cgi-bin/fbidq.html?FBgn0033367) |
| [CG8577](http://flybase.org/cgi-bin/fbidq.html?FBgn0033327) | PGRP-SC1b | [PGRP-SC1b](http://flybase.org/cgi-bin/fbidq.html?FBgn0033327) |
| [CG8896](http://flybase.org/cgi-bin/fbidq.html?FBgn0004364) | 18 wheeler | [18w](http://flybase.org/cgi-bin/fbidq.html?FBgn0004364) |
| [CG9453](http://flybase.org/cgi-bin/fbidq.html?FBgn0028985) | Serine protease inhibitor 4 | [Spn4](http://flybase.org/cgi-bin/fbidq.html?FBgn0028985) |
| [CG9681](http://flybase.org/cgi-bin/fbidq.html?FBgn0043578) | PGRP-SB1 | [PGRP-SB1](http://flybase.org/cgi-bin/fbidq.html?FBgn0043578) |
| [CG11992](http://flybase.org/cgi-bin/fbidq.html?FBgn0014018) | Relish | [Rel](http://flybase.org/cgi-bin/fbidq.html?FBgn0014018) |
| [CG13905](http://flybase.org/cgi-bin/fbidq.html?FBgn0035176) | - | [CG13905](http://flybase.org/cgi-bin/fbidq.html?FBgn0035176) |
| [CG15282](http://flybase.org/cgi-bin/fbidq.html?FBgn0028855) | - | [CG15282](http://flybase.org/cgi-bin/fbidq.html?FBgn0028855) |
| [CG15829](http://flybase.org/cgi-bin/fbidq.html?FBgn0035743) | - | [CG15829](http://flybase.org/cgi-bin/fbidq.html?FBgn0035743) |
| [CG16743](http://flybase.org/cgi-bin/fbidq.html?FBgn0032322) | - | [CG16743](http://flybase.org/cgi-bin/fbidq.html?FBgn0032322) |
| [CG16756](http://flybase.org/cgi-bin/fbidq.html?FBgn0029765) | - | [CG16756](http://flybase.org/cgi-bin/fbidq.html?FBgn0029765) |
| [CG31507](http://flybase.org/cgi-bin/fbidq.html?FBgn0044809) | Turandot Z | [TotZ](http://flybase.org/cgi-bin/fbidq.html?FBgn0044809) |

**Common genes: Drosophila Sal Gl IMD upregulated vs Susceptible genes for oral infection in a genome wide RNAi screen (Cronin et al. 2009).**

| **SUBMITTED ID** | **NAME** | **SYMBOL** |
| --- | --- | --- |
| [CG10992](http://flybase.org/cgi-bin/fbidq.html?FBgn0030521) | - | [CG10992](http://flybase.org/cgi-bin/fbidq.html?FBgn0030521) |
| [CG11051](http://flybase.org/cgi-bin/fbidq.html?FBgn0040813) | Neuropeptide-like precursor 2 | [Nplp2](http://flybase.org/cgi-bin/fbidq.html?FBgn0040813) |
| [CG11501](http://flybase.org/cgi-bin/fbidq.html?FBgn0039666) | - | [CG11501](http://flybase.org/cgi-bin/fbidq.html?FBgn0039666) |
| [CG12323](http://flybase.org/cgi-bin/fbidq.html?FBgn0029134) | Proteasome beta5 subunit | [Prosbeta5](http://flybase.org/cgi-bin/fbidq.html?FBgn0029134) |
| [CG12770](http://flybase.org/cgi-bin/fbidq.html?FBgn0021814) | Vacuolar protein sorting 28 | [Vps28](http://flybase.org/cgi-bin/fbidq.html?FBgn0021814) |
| [CG14214](http://flybase.org/cgi-bin/fbidq.html?FBgn0031049) | Sec61gamma | [Sec61gamma](http://flybase.org/cgi-bin/fbidq.html?FBgn0031049) |
| [CG1662](http://flybase.org/cgi-bin/fbidq.html?FBgn0030481) | - | [CG1662](http://flybase.org/cgi-bin/fbidq.html?FBgn0030481) |
| [CG18210](http://flybase.org/cgi-bin/fbidq.html?FBgn0030636) | - | [CG18210](http://flybase.org/cgi-bin/fbidq.html?FBgn0030636) |
| [CG18624](http://flybase.org/cgi-bin/fbidq.html?FBgn0029971) | - | [CG18624](http://flybase.org/cgi-bin/fbidq.html?FBgn0029971) |
| [CG2291](http://flybase.org/cgi-bin/fbidq.html?FBgn0033279) | - | [CG2291](http://flybase.org/cgi-bin/fbidq.html?FBgn0033279) |
| [CG2358](http://flybase.org/cgi-bin/fbidq.html?FBgn0026567) | Spase 18/21-subunit | [Spase18-21](http://flybase.org/cgi-bin/fbidq.html?FBgn0026567) |
| [CG2934](http://flybase.org/cgi-bin/fbidq.html?FBgn0028665) | Vacuolar H[+]-ATPase C39 subunit | [VhaAC39](http://flybase.org/cgi-bin/fbidq.html?FBgn0028665) |
| [CG30382](http://flybase.org/cgi-bin/fbidq.html?FBgn0050382) | - | [CG30382](http://flybase.org/cgi-bin/fbidq.html?FBgn0050382) |
| [CG31370](http://flybase.org/cgi-bin/fbidq.html?FBgn0051370) | - | [CG31370](http://flybase.org/cgi-bin/fbidq.html?FBgn0051370) |
| [CG31715](http://flybase.org/cgi-bin/fbidq.html?FBgn0051715) | - | [CG31715](http://flybase.org/cgi-bin/fbidq.html?FBgn0051715) |
| [CG32198](http://flybase.org/cgi-bin/fbidq.html?FBgn0052198) | - | [CG32198](http://flybase.org/cgi-bin/fbidq.html?FBgn0052198) |
| [CG3314](http://flybase.org/cgi-bin/fbidq.html?FBgn0014026) | Ribosomal protein L7A | [RpL7A](http://flybase.org/cgi-bin/fbidq.html?FBgn0014026) |
| [CG3773](http://flybase.org/cgi-bin/fbidq.html?FBgn0038692) | - | [CG3773](http://flybase.org/cgi-bin/fbidq.html?FBgn0038692) |
| [CG4097](http://flybase.org/cgi-bin/fbidq.html?FBgn0002284) | Proteasome 26kD subunit | [Pros26](http://flybase.org/cgi-bin/fbidq.html?FBgn0002284) |
| [CG4957](http://flybase.org/cgi-bin/fbidq.html?FBgn0032205) | - | [CG4957](http://flybase.org/cgi-bin/fbidq.html?FBgn0032205) |
| [CG5864](http://flybase.org/cgi-bin/fbidq.html?FBgn0039132) | AP-1sigma | [AP-1sigma](http://flybase.org/cgi-bin/fbidq.html?FBgn0039132) |
| [CG5915](http://flybase.org/cgi-bin/fbidq.html?FBgn0015795) | Rab-protein 7 | [Rab7](http://flybase.org/cgi-bin/fbidq.html?FBgn0015795) |
| [CG5989](http://flybase.org/cgi-bin/fbidq.html?FBgn0017429) | - | [CG5989](http://flybase.org/cgi-bin/fbidq.html?FBgn0017429) |
| [CG6272](http://flybase.org/cgi-bin/fbidq.html?FBgn0036126) | - | [CG6272](http://flybase.org/cgi-bin/fbidq.html?FBgn0036126) |
| [CG7123](http://flybase.org/cgi-bin/fbidq.html?FBgn0002527) | Laminin B1 | [LanB1](http://flybase.org/cgi-bin/fbidq.html?FBgn0002527) |
| [CG7961](http://flybase.org/cgi-bin/fbidq.html?FBgn0025725) | alpha-coatomer protein | [alphaCop](http://flybase.org/cgi-bin/fbidq.html?FBgn0025725) |
| [CG8472](http://flybase.org/cgi-bin/fbidq.html?FBgn0000253) | Calmodulin | [Cam](http://flybase.org/cgi-bin/fbidq.html?FBgn0000253) |
| [CG8922](http://flybase.org/cgi-bin/fbidq.html?FBgn0002590) | Ribosomal protein S5a | [RpS5a](http://flybase.org/cgi-bin/fbidq.html?FBgn0002590) |
| [CG9553](http://flybase.org/cgi-bin/fbidq.html?FBgn0000308) | chickadee | [chic](http://flybase.org/cgi-bin/fbidq.html?FBgn0000308) |
| [CG9947](http://flybase.org/cgi-bin/fbidq.html?FBgn0030752) | - | [CG9947](http://flybase.org/cgi-bin/fbidq.html?FBgn0030752) |
| [CG31704](http://flybase.org/cgi-bin/fbidq.html?FBgn0051704) | - | [CG31704](http://flybase.org/cgi-bin/fbidq.html?FBgn0051704) |
