# Supplementary material for: Chronic activation of the epithelial immune system of the fruit fly's salivary glands has a negative effect on organismal growth and induces a peculiar set of target genes
Source: BMC Genomics. 2010 Apr 26;11:265. doi: 10.1186/1471-2164-11-265 (PMC2874812; doi:10.1186/1471-2164-11-265)
Supplement: Additional file 2 — Venn diagram analysis of Drosophila Salivary glands genes upregulated following IMD-pathway activation with various gene lists involved in reaction to infection. This file contains lists of those genes from Venn-diagram analyses of genes upregulated in the salivary glands following IMD-activation with various other sets of genes derived from infection experiments of different tissues. [file 1471-2164-11-265-S2.DOC]

**Common genes: Drosophila Sal Gl IMD upregulated vs Drosophila trachea immune reaction (Erwinia carotovora) upregulated (Wagner et al. 2009)**

| **SUBMITTED ID** | **NAME** | **SYMBOL** |
| --- | --- | --- |
| [CG32282](http://flybase.org/cgi-bin/fbidq.html?FBgn0052282) | drosomycin-4 | [dro4](http://flybase.org/cgi-bin/fbidq.html?FBgn0052282) |
| [CG3348](http://flybase.org/cgi-bin/fbidq.html?FBgn0040609) | - | [CG3348](http://flybase.org/cgi-bin/fbidq.html?FBgn0040609) |
| [CG8891](http://flybase.org/cgi-bin/fbidq.html?FBgn0031663) | - | [CG8891](http://flybase.org/cgi-bin/fbidq.html?FBgn0031663) |
| [CG9080](http://flybase.org/cgi-bin/fbidq.html?FBgn0033593) | - | [CG9080](http://flybase.org/cgi-bin/fbidq.html?FBgn0033593) |
| [CG10527](http://flybase.org/cgi-bin/fbidq.html?FBgn0034583) | - | [CG10527](http://flybase.org/cgi-bin/fbidq.html?FBgn0034583) |
| [CG11015](http://flybase.org/cgi-bin/fbidq.html?FBgn0031830) | - | [CG11015](http://flybase.org/cgi-bin/fbidq.html?FBgn0031830) |
| [CG11840](http://flybase.org/cgi-bin/fbidq.html?FBgn0031260) | Signal peptide protease | [Spp](http://flybase.org/cgi-bin/fbidq.html?FBgn0031260) |
| [CG11981](http://flybase.org/cgi-bin/fbidq.html?FBgn0026380) | Proteasome beta3 subunit | [Prosbeta3](http://flybase.org/cgi-bin/fbidq.html?FBgn0026380) |
| [CG12012](http://flybase.org/cgi-bin/fbidq.html?FBgn0035444) | - | [CG12012](http://flybase.org/cgi-bin/fbidq.html?FBgn0035444) |
| [CG13315](http://flybase.org/cgi-bin/fbidq.html?FBgn0040827) | - | [CG13315](http://flybase.org/cgi-bin/fbidq.html?FBgn0040827) |
| [CG17691](http://flybase.org/cgi-bin/fbidq.html?FBgn0039993) | - | [CG17691](http://flybase.org/cgi-bin/fbidq.html?FBgn0039993) |
| [CG32446](http://flybase.org/cgi-bin/fbidq.html?FBgn0052446) | - | [Atox1](http://flybase.org/cgi-bin/fbidq.html?FBgn0052446) |
| [CG32954](http://flybase.org/cgi-bin/fbidq.html?FBgn0000056) | Adh-related | [Adhr](http://flybase.org/cgi-bin/fbidq.html?FBgn0000056) |
| [CG3683](http://flybase.org/cgi-bin/fbidq.html?FBgn0035046) | - | [CG3683](http://flybase.org/cgi-bin/fbidq.html?FBgn0035046) |
| [CG7188](http://flybase.org/cgi-bin/fbidq.html?FBgn0035871) | - | [CG7188](http://flybase.org/cgi-bin/fbidq.html?FBgn0035871) |

**Common genes: Drosophila Sal Gl IMD upregulated vs Drosophila trachea ectopic IMD activation upregulated (Wagner et al. 2009)**

| **SUBMITTED ID** | **NAME** | **SYMBOL** |
| --- | --- | --- |
| [CG11858](http://flybase.org/cgi-bin/fbidq.html?FBgn0039305) | - | [CG11858](http://flybase.org/cgi-bin/fbidq.html?FBgn0039305) |
| [CG11859](http://flybase.org/cgi-bin/fbidq.html?FBgn0039306) | - | [CG11859](http://flybase.org/cgi-bin/fbidq.html?FBgn0039306) |
| [CG11906](http://flybase.org/cgi-bin/fbidq.html?FBgn0034425) | - | [CG11906](http://flybase.org/cgi-bin/fbidq.html?FBgn0034425) |
| [CG14619](http://flybase.org/cgi-bin/fbidq.html?FBgn0031187) | - | [CG14619](http://flybase.org/cgi-bin/fbidq.html?FBgn0031187) |
| [CG1532](http://flybase.org/cgi-bin/fbidq.html?FBgn0031143) | - | [CG1532](http://flybase.org/cgi-bin/fbidq.html?FBgn0031143) |
| [CG30382](http://flybase.org/cgi-bin/fbidq.html?FBgn0050382) | - | [CG30382](http://flybase.org/cgi-bin/fbidq.html?FBgn0050382) |
| [CG32640](http://flybase.org/cgi-bin/fbidq.html?FBgn0052640) | - | [CG32640](http://flybase.org/cgi-bin/fbidq.html?FBgn0052640) |
| [CG4463](http://flybase.org/cgi-bin/fbidq.html?FBgn0001224) | Heat shock protein 23 | [Hsp23](http://flybase.org/cgi-bin/fbidq.html?FBgn0001224) |
| [CG4878](http://flybase.org/cgi-bin/fbidq.html?FBgn0034237) | eIF3-S9 | [eIF3-S9](http://flybase.org/cgi-bin/fbidq.html?FBgn0034237) |
| [CG5214](http://flybase.org/cgi-bin/fbidq.html?FBgn0037891) | - | [CG5214](http://flybase.org/cgi-bin/fbidq.html?FBgn0037891) |
| [CG5773](http://flybase.org/cgi-bin/fbidq.html?FBgn0034290) | - | [CG5773](http://flybase.org/cgi-bin/fbidq.html?FBgn0034290) |
| [CG6272](http://flybase.org/cgi-bin/fbidq.html?FBgn0036126) | - | [CG6272](http://flybase.org/cgi-bin/fbidq.html?FBgn0036126) |
| [CG6523](http://flybase.org/cgi-bin/fbidq.html?FBgn0032509) | - | [CG6523](http://flybase.org/cgi-bin/fbidq.html?FBgn0032509) |
| [CG8945](http://flybase.org/cgi-bin/fbidq.html?FBgn0030815) | - | [CG8945](http://flybase.org/cgi-bin/fbidq.html?FBgn0030815) |
| [CG9296](http://flybase.org/cgi-bin/fbidq.html?FBgn0032059) | Prenyl-binding protein | [PrBP](http://flybase.org/cgi-bin/fbidq.html?FBgn0032059) |
| [CG32282](http://flybase.org/cgi-bin/fbidq.html?FBgn0052282) | drosomycin-4 | [dro4](http://flybase.org/cgi-bin/fbidq.html?FBgn0052282) |
| [CG3348](http://flybase.org/cgi-bin/fbidq.html?FBgn0040609) | - | [CG3348](http://flybase.org/cgi-bin/fbidq.html?FBgn0040609) |
| [CG8891](http://flybase.org/cgi-bin/fbidq.html?FBgn0031663) | - | [CG8891](http://flybase.org/cgi-bin/fbidq.html?FBgn0031663) |
| [CG9080](http://flybase.org/cgi-bin/fbidq.html?FBgn0033593) | - | [CG9080](http://flybase.org/cgi-bin/fbidq.html?FBgn0033593) |

**Common genes: Drosophila Sal Gl IMD upregulated vs Drosophila gut infection (Erwinia carotovora) upregulated (Wagner et al. 2009)**

| **SUBMITTED ID** | **NAME** | **SYMBOL** |
| --- | --- | --- |
| [CG11750](http://flybase.org/cgi-bin/fbidq.html?FBgn0030294) | - | [CG11750](http://flybase.org/cgi-bin/fbidq.html?FBgn0030294) |
| [CG12918](http://flybase.org/cgi-bin/fbidq.html?FBgn0033477) | - | [CG12918](http://flybase.org/cgi-bin/fbidq.html?FBgn0033477) |
| [CG1572](http://flybase.org/cgi-bin/fbidq.html?FBgn0030309) | - | [CG1572](http://flybase.org/cgi-bin/fbidq.html?FBgn0030309) |
| [CG17294](http://flybase.org/cgi-bin/fbidq.html?FBgn0032032) | - | [CG17294](http://flybase.org/cgi-bin/fbidq.html?FBgn0032032) |
| [CG17949](http://flybase.org/cgi-bin/fbidq.html?FBgn0061209) | His2B:CG17949 | [His2B:CG17949](http://flybase.org/cgi-bin/fbidq.html?FBgn0061209) |
| [CG18212](http://flybase.org/cgi-bin/fbidq.html?FBgn0038535) | aluminum tubes | [alt](http://flybase.org/cgi-bin/fbidq.html?FBgn0038535) |
| [CG31363](http://flybase.org/cgi-bin/fbidq.html?FBgn0051363) | Jupiter | [Jupiter](http://flybase.org/cgi-bin/fbidq.html?FBgn0051363) |
| [CG31704](http://flybase.org/cgi-bin/fbidq.html?FBgn0051704) | - | [CG31704](http://flybase.org/cgi-bin/fbidq.html?FBgn0051704) |
| [CG3172](http://flybase.org/cgi-bin/fbidq.html?FBgn0038206) | twinfilin | [twf](http://flybase.org/cgi-bin/fbidq.html?FBgn0038206) |
| [CG4463](http://flybase.org/cgi-bin/fbidq.html?FBgn0001224) | Heat shock protein 23 | [Hsp23](http://flybase.org/cgi-bin/fbidq.html?FBgn0001224) |
| [CG4600](http://flybase.org/cgi-bin/fbidq.html?FBgn0040064) | yippee interacting protein 2 | [yip2](http://flybase.org/cgi-bin/fbidq.html?FBgn0040064) |
| [CG5371](http://flybase.org/cgi-bin/fbidq.html?FBgn0011703) | Ribonucleoside diphosphate reductase large subunit | [RnrL](http://flybase.org/cgi-bin/fbidq.html?FBgn0011703) |
| [CG7668](http://flybase.org/cgi-bin/fbidq.html?FBgn0036929) | - | [CG7668](http://flybase.org/cgi-bin/fbidq.html?FBgn0036929) |
| [CG32282](http://flybase.org/cgi-bin/fbidq.html?FBgn0052282) | drosomycin-4 | [dro4](http://flybase.org/cgi-bin/fbidq.html?FBgn0052282) |
| [CG9080](http://flybase.org/cgi-bin/fbidq.html?FBgn0033593) | - | [CG9080](http://flybase.org/cgi-bin/fbidq.html?FBgn0033593) |
