# Supplementary material for: Chronic activation of the epithelial immune system of the fruit fly's salivary glands has a negative effect on organismal growth and induces a peculiar set of target genes
Source: BMC Genomics. 2010 Apr 26;11:265. doi: 10.1186/1471-2164-11-265 (PMC2874812; doi:10.1186/1471-2164-11-265)
Supplement: Additional file 3 — Drosophila Salivary glands: Genes upregulated following IMD-pathway activation. This file contains a list of those genes whose expression in the salivary glands is upregulated significantly following activation of the IMD-pathway. [file 1471-2164-11-265-S3.DOC]

**Drosophila Salivary glands: Genes upregulated following IMD-activation**

| **SUBMITTED ID** | **NAME** | **SYMBOL** |
| --- | --- | --- |
| [CG10157](http://flybase.org/cgi-bin/fbidq.html?FBgn0039099) | - | [CG10157](http://flybase.org/cgi-bin/fbidq.html?FBgn0039099) |
| [CG10166](http://flybase.org/cgi-bin/fbidq.html?FBgn0032799) | - | [CG10166](http://flybase.org/cgi-bin/fbidq.html?FBgn0032799) |
| [CG10230](http://flybase.org/cgi-bin/fbidq.html?FBgn0028691) | Rpn9 | [Rpn9](http://flybase.org/cgi-bin/fbidq.html?FBgn0028691) |
| [CG10272](http://flybase.org/cgi-bin/fbidq.html?FBgn0014963) | grappa | [gpp](http://flybase.org/cgi-bin/fbidq.html?FBgn0014963) |
| [CG10306](http://flybase.org/cgi-bin/fbidq.html?FBgn0034654) | - | [CG10306](http://flybase.org/cgi-bin/fbidq.html?FBgn0034654) |
| [CG10373](http://flybase.org/cgi-bin/fbidq.html?FBgn0032704) | - | [CG10373](http://flybase.org/cgi-bin/fbidq.html?FBgn0032704) |
| [CG10423](http://flybase.org/cgi-bin/fbidq.html?FBgn0039300) | Ribosomal protein S27 | [RpS27](http://flybase.org/cgi-bin/fbidq.html?FBgn0039300) |
| [CG10445](http://flybase.org/cgi-bin/fbidq.html?FBgn0037531) | - | [CG10445](http://flybase.org/cgi-bin/fbidq.html?FBgn0037531) |
| [CG10449](http://flybase.org/cgi-bin/fbidq.html?FBgn0002022) | Catecholamines up | [Catsup](http://flybase.org/cgi-bin/fbidq.html?FBgn0002022) |
| [CG10460](http://flybase.org/cgi-bin/fbidq.html?FBgn0034443) | crammer | [cer](http://flybase.org/cgi-bin/fbidq.html?FBgn0034443) |
| [CG10463](http://flybase.org/cgi-bin/fbidq.html?FBgn0032819) | - | [CG10463](http://flybase.org/cgi-bin/fbidq.html?FBgn0032819) |
| [CG10481](http://flybase.org/cgi-bin/fbidq.html?FBgn0032827) | - | [CG10481](http://flybase.org/cgi-bin/fbidq.html?FBgn0032827) |
| [CG10520](http://flybase.org/cgi-bin/fbidq.html?FBgn0003882) | tube | [tub](http://flybase.org/cgi-bin/fbidq.html?FBgn0003882) |
| [CG10527](http://flybase.org/cgi-bin/fbidq.html?FBgn0034583) | - | [CG10527](http://flybase.org/cgi-bin/fbidq.html?FBgn0034583) |
| [CG10640](http://flybase.org/cgi-bin/fbidq.html?FBgn0035601) | Uev1A | [Uev1A](http://flybase.org/cgi-bin/fbidq.html?FBgn0035601) |
| [CG10658](http://flybase.org/cgi-bin/fbidq.html?FBgn0014000) | Helical Factor | [Hf](http://flybase.org/cgi-bin/fbidq.html?FBgn0014000) |
| [CG10679](http://flybase.org/cgi-bin/fbidq.html?FBgn0032725) | Nedd8 | [Nedd8](http://flybase.org/cgi-bin/fbidq.html?FBgn0032725) |
| [CG10688](http://flybase.org/cgi-bin/fbidq.html?FBgn0036300) | - | [CG10688](http://flybase.org/cgi-bin/fbidq.html?FBgn0036300) |
| [CG1077](http://flybase.org/cgi-bin/fbidq.html?FBgn0037405) | - | [CG1077](http://flybase.org/cgi-bin/fbidq.html?FBgn0037405) |
| [CG10802](http://flybase.org/cgi-bin/fbidq.html?FBgn0029664) | - | [CG10802](http://flybase.org/cgi-bin/fbidq.html?FBgn0029664) |
| [CG10825](http://flybase.org/cgi-bin/fbidq.html?FBgn0038860) | - | [CG10825](http://flybase.org/cgi-bin/fbidq.html?FBgn0038860) |
| [CG10904](http://flybase.org/cgi-bin/fbidq.html?FBgn0034945) | - | [CG10904](http://flybase.org/cgi-bin/fbidq.html?FBgn0034945) |
| [CG10933](http://flybase.org/cgi-bin/fbidq.html?FBgn0034264) | - | [CG10933](http://flybase.org/cgi-bin/fbidq.html?FBgn0034264) |
| [CG10949](http://flybase.org/cgi-bin/fbidq.html?FBgn0032858) | - | [CG10949](http://flybase.org/cgi-bin/fbidq.html?FBgn0032858) |
| [CG10992](http://flybase.org/cgi-bin/fbidq.html?FBgn0030521) | - | [CG10992](http://flybase.org/cgi-bin/fbidq.html?FBgn0030521) |
| [CG11015](http://flybase.org/cgi-bin/fbidq.html?FBgn0031830) | - | [CG11015](http://flybase.org/cgi-bin/fbidq.html?FBgn0031830) |
| [CG11027](http://flybase.org/cgi-bin/fbidq.html?FBgn0013749) | ADP ribosylation factor 102F | [Arf102F](http://flybase.org/cgi-bin/fbidq.html?FBgn0013749) |
| [CG11043](http://flybase.org/cgi-bin/fbidq.html?FBgn0031831) | - | [CG11043](http://flybase.org/cgi-bin/fbidq.html?FBgn0031831) |
| [CG11051](http://flybase.org/cgi-bin/fbidq.html?FBgn0040813) | Neuropeptide-like precursor 2 | [Nplp2](http://flybase.org/cgi-bin/fbidq.html?FBgn0040813) |
| [CG1108](http://flybase.org/cgi-bin/fbidq.html?FBgn0015574) | alpha-Esterase-6 | [alpha-Est6](http://flybase.org/cgi-bin/fbidq.html?FBgn0015574) |
| [CG11103](http://flybase.org/cgi-bin/fbidq.html?FBgn0030522) | - | [CG11103](http://flybase.org/cgi-bin/fbidq.html?FBgn0030522) |
| [CG11154](http://flybase.org/cgi-bin/fbidq.html?FBgn0010217) | ATP synthase-beta | [ATPsyn-beta](http://flybase.org/cgi-bin/fbidq.html?FBgn0010217) |
| [CG11403](http://flybase.org/cgi-bin/fbidq.html?FBgn0026876) | - | [CG11403](http://flybase.org/cgi-bin/fbidq.html?FBgn0026876) |
| [CG11444](http://flybase.org/cgi-bin/fbidq.html?FBgn0029715) | - | [CG11444](http://flybase.org/cgi-bin/fbidq.html?FBgn0029715) |
| [CG11482](http://flybase.org/cgi-bin/fbidq.html?FBgn0011659) | Mlh1 | [Mlh1](http://flybase.org/cgi-bin/fbidq.html?FBgn0011659) |
| [CG11500](http://flybase.org/cgi-bin/fbidq.html?FBgn0040623) | Spase 12-subunit | [Spase12](http://flybase.org/cgi-bin/fbidq.html?FBgn0040623) |
| [CG11501](http://flybase.org/cgi-bin/fbidq.html?FBgn0039666) | - | [CG11501](http://flybase.org/cgi-bin/fbidq.html?FBgn0039666) |
| [CG11522](http://flybase.org/cgi-bin/fbidq.html?FBgn0039857) | Ribosomal protein L6 | [RpL6](http://flybase.org/cgi-bin/fbidq.html?FBgn0039857) |
| [CG11527](http://flybase.org/cgi-bin/fbidq.html?FBgn0011722) | Tiggrin | [Tig](http://flybase.org/cgi-bin/fbidq.html?FBgn0011722) |
| [CG11611](http://flybase.org/cgi-bin/fbidq.html?FBgn0036204) | Tim13 | [Tim13](http://flybase.org/cgi-bin/fbidq.html?FBgn0036204) |
| [CG1163](http://flybase.org/cgi-bin/fbidq.html?FBgn0003275) | RNA polymerase II 18kD subunit | [RpII18](http://flybase.org/cgi-bin/fbidq.html?FBgn0003275) |
| [CG11750](http://flybase.org/cgi-bin/fbidq.html?FBgn0030294) | - | [CG11750](http://flybase.org/cgi-bin/fbidq.html?FBgn0030294) |
| [CG11790](http://flybase.org/cgi-bin/fbidq.html?FBgn0039265) | - | [CG11790](http://flybase.org/cgi-bin/fbidq.html?FBgn0039265) |
| [CG11793](http://flybase.org/cgi-bin/fbidq.html?FBgn0003462) | Superoxide dismutase | [Sod](http://flybase.org/cgi-bin/fbidq.html?FBgn0003462) |
| [CG11840](http://flybase.org/cgi-bin/fbidq.html?FBgn0031260) | Signal peptide protease | [Spp](http://flybase.org/cgi-bin/fbidq.html?FBgn0031260) |
| [CG11858](http://flybase.org/cgi-bin/fbidq.html?FBgn0039305) | - | [CG11858](http://flybase.org/cgi-bin/fbidq.html?FBgn0039305) |
| [CG11859](http://flybase.org/cgi-bin/fbidq.html?FBgn0039306) | - | [CG11859](http://flybase.org/cgi-bin/fbidq.html?FBgn0039306) |
| [CG11906](http://flybase.org/cgi-bin/fbidq.html?FBgn0034425) | - | [CG11906](http://flybase.org/cgi-bin/fbidq.html?FBgn0034425) |
| [CG11980](http://flybase.org/cgi-bin/fbidq.html?FBgn0037652) | - | [CG11980](http://flybase.org/cgi-bin/fbidq.html?FBgn0037652) |
| [CG11981](http://flybase.org/cgi-bin/fbidq.html?FBgn0026380) | Proteasome beta3 subunit | [Prosbeta3](http://flybase.org/cgi-bin/fbidq.html?FBgn0026380) |
| [CG11985](http://flybase.org/cgi-bin/fbidq.html?FBgn0040534) | - | [CG11985](http://flybase.org/cgi-bin/fbidq.html?FBgn0040534) |
| [CG12012](http://flybase.org/cgi-bin/fbidq.html?FBgn0035444) | - | [CG12012](http://flybase.org/cgi-bin/fbidq.html?FBgn0035444) |
| [CG12045](http://flybase.org/cgi-bin/fbidq.html?FBgn0039805) | Cuticular protein 100A | [Cpr100A](http://flybase.org/cgi-bin/fbidq.html?FBgn0039805) |
| [CG12081](http://flybase.org/cgi-bin/fbidq.html?FBgn0030053) | - | [CG12081](http://flybase.org/cgi-bin/fbidq.html?FBgn0030053) |
| [CG12220](http://flybase.org/cgi-bin/fbidq.html?FBgn0039835) | mitochondrial ribosomal protein L32 | [mRpL32](http://flybase.org/cgi-bin/fbidq.html?FBgn0039835) |
| [CG12323](http://flybase.org/cgi-bin/fbidq.html?FBgn0029134) | Proteasome beta5 subunit | [Prosbeta5](http://flybase.org/cgi-bin/fbidq.html?FBgn0029134) |
| [CG12324](http://flybase.org/cgi-bin/fbidq.html?FBgn0033555) | Ribosomal protein S15Ab | [RpS15Ab](http://flybase.org/cgi-bin/fbidq.html?FBgn0033555) |
| [CG12346](http://flybase.org/cgi-bin/fbidq.html?FBgn0017414) | cag | [cag](http://flybase.org/cgi-bin/fbidq.html?FBgn0017414) |
| [CG12350](http://flybase.org/cgi-bin/fbidq.html?FBgn0043470) | lambdaTry | [lambdaTry](http://flybase.org/cgi-bin/fbidq.html?FBgn0043470) |
| [CG1236](http://flybase.org/cgi-bin/fbidq.html?FBgn0037370) | - | [CG1236](http://flybase.org/cgi-bin/fbidq.html?FBgn0037370) |
| [CG12372](http://flybase.org/cgi-bin/fbidq.html?FBgn0028683) | spt4 | [spt4](http://flybase.org/cgi-bin/fbidq.html?FBgn0028683) |
| [CG12384](http://flybase.org/cgi-bin/fbidq.html?FBgn0033624) | - | [CG12384](http://flybase.org/cgi-bin/fbidq.html?FBgn0033624) |
| [CG12478](http://flybase.org/cgi-bin/fbidq.html?FBgn0036379) | bruno-3 | [bru-3](http://flybase.org/cgi-bin/fbidq.html?FBgn0036379) |
| [CG1249](http://flybase.org/cgi-bin/fbidq.html?FBgn0037434) | snRNP2 | [snRNP2](http://flybase.org/cgi-bin/fbidq.html?FBgn0037434) |
| [CG12508](http://flybase.org/cgi-bin/fbidq.html?FBgn0040995) | - | [CG12508](http://flybase.org/cgi-bin/fbidq.html?FBgn0040995) |
| [CG12522](http://flybase.org/cgi-bin/fbidq.html?FBgn0036131) | - | [CG12522](http://flybase.org/cgi-bin/fbidq.html?FBgn0036131) |
| [CG12546](http://flybase.org/cgi-bin/fbidq.html?FBgn0037178) | - | [CG12546](http://flybase.org/cgi-bin/fbidq.html?FBgn0037178) |
| [CG12551](http://flybase.org/cgi-bin/fbidq.html?FBgn0033045) | - | [CG12551](http://flybase.org/cgi-bin/fbidq.html?FBgn0033045) |
| [CG12592](http://flybase.org/cgi-bin/fbidq.html?FBgn0037811) | - | [CG12592](http://flybase.org/cgi-bin/fbidq.html?FBgn0037811) |
| [CG12620](http://flybase.org/cgi-bin/fbidq.html?FBgn0032626) | - | [CG12620](http://flybase.org/cgi-bin/fbidq.html?FBgn0032626) |
| [CG1275](http://flybase.org/cgi-bin/fbidq.html?FBgn0035321) | - | [CG1275](http://flybase.org/cgi-bin/fbidq.html?FBgn0035321) |
| [CG12770](http://flybase.org/cgi-bin/fbidq.html?FBgn0021814) | Vacuolar protein sorting 28 | [Vps28](http://flybase.org/cgi-bin/fbidq.html?FBgn0021814) |
| [CG12795](http://flybase.org/cgi-bin/fbidq.html?FBgn0031535) | - | [CG12795](http://flybase.org/cgi-bin/fbidq.html?FBgn0031535) |
| [CG12811](http://flybase.org/cgi-bin/fbidq.html?FBgn0037779) | - | [CG12811](http://flybase.org/cgi-bin/fbidq.html?FBgn0037779) |
| [CG12846](http://flybase.org/cgi-bin/fbidq.html?FBgn0029507) | Tetraspanin 42Ed | [Tsp42Ed](http://flybase.org/cgi-bin/fbidq.html?FBgn0029507) |
| [CG12848](http://flybase.org/cgi-bin/fbidq.html?FBgn0040666) | - | [CG12848](http://flybase.org/cgi-bin/fbidq.html?FBgn0040666) |
| [CG12918](http://flybase.org/cgi-bin/fbidq.html?FBgn0033477) | - | [CG12918](http://flybase.org/cgi-bin/fbidq.html?FBgn0033477) |
| [CG1298](http://flybase.org/cgi-bin/fbidq.html?FBgn0033032) | - | [CG1298](http://flybase.org/cgi-bin/fbidq.html?FBgn0033032) |
| [CG1320](http://flybase.org/cgi-bin/fbidq.html?FBgn0035335) | mitochondrial ribosomal protein L23 | [mRpL23](http://flybase.org/cgi-bin/fbidq.html?FBgn0035335) |
| [CG13216](http://flybase.org/cgi-bin/fbidq.html?FBgn0033591) | - | [CG13216](http://flybase.org/cgi-bin/fbidq.html?FBgn0033591) |
| [CG13220](http://flybase.org/cgi-bin/fbidq.html?FBgn0033608) | - | [CG13220](http://flybase.org/cgi-bin/fbidq.html?FBgn0033608) |
| [CG13231](http://flybase.org/cgi-bin/fbidq.html?FBgn0033580) | - | [CG13231](http://flybase.org/cgi-bin/fbidq.html?FBgn0033580) |
| [CG13297](http://flybase.org/cgi-bin/fbidq.html?FBgn0035685) | - | [CG13297](http://flybase.org/cgi-bin/fbidq.html?FBgn0035685) |
| [CG13298](http://flybase.org/cgi-bin/fbidq.html?FBgn0035692) | - | [CG13298](http://flybase.org/cgi-bin/fbidq.html?FBgn0035692) |
| [CG13315](http://flybase.org/cgi-bin/fbidq.html?FBgn0040827) | - | [CG13315](http://flybase.org/cgi-bin/fbidq.html?FBgn0040827) |
| [CG13393](http://flybase.org/cgi-bin/fbidq.html?FBgn0032035) | - | [CG13393](http://flybase.org/cgi-bin/fbidq.html?FBgn0032035) |
| [CG13460](http://flybase.org/cgi-bin/fbidq.html?FBgn0036471) | - | [CG13460](http://flybase.org/cgi-bin/fbidq.html?FBgn0036471) |
| [CG13590](http://flybase.org/cgi-bin/fbidq.html?FBgn0035012) | - | [CG13590](http://flybase.org/cgi-bin/fbidq.html?FBgn0035012) |
| [CG13749](http://flybase.org/cgi-bin/fbidq.html?FBgn0033353) | - | [CG13749](http://flybase.org/cgi-bin/fbidq.html?FBgn0033353) |
| [CG1381](http://flybase.org/cgi-bin/fbidq.html?FBgn0033485) | - | [CG1381](http://flybase.org/cgi-bin/fbidq.html?FBgn0033485) |
| [CG13877](http://flybase.org/cgi-bin/fbidq.html?FBgn0035112) | - | [CG13877](http://flybase.org/cgi-bin/fbidq.html?FBgn0035112) |
| [CG13962](http://flybase.org/cgi-bin/fbidq.html?FBgn0032824) | - | [CG13962](http://flybase.org/cgi-bin/fbidq.html?FBgn0032824) |
| [CG1404](http://flybase.org/cgi-bin/fbidq.html?FBgn0020255) | ran | [ran](http://flybase.org/cgi-bin/fbidq.html?FBgn0020255) |
| [CG14084](http://flybase.org/cgi-bin/fbidq.html?FBgn0260857) | - | [Bet1](http://flybase.org/cgi-bin/fbidq.html?FBgn0260857) |
| [CG14109](http://flybase.org/cgi-bin/fbidq.html?FBgn0036364) | - | [CG14109](http://flybase.org/cgi-bin/fbidq.html?FBgn0036364) |
| [CG14207](http://flybase.org/cgi-bin/fbidq.html?FBgn0031037) | - | [CG14207](http://flybase.org/cgi-bin/fbidq.html?FBgn0031037) |
| [CG14214](http://flybase.org/cgi-bin/fbidq.html?FBgn0031049) | Sec61gamma | [Sec61gamma](http://flybase.org/cgi-bin/fbidq.html?FBgn0031049) |
| [CG14237](http://flybase.org/cgi-bin/fbidq.html?FBgn0039428) | - | [CG14237](http://flybase.org/cgi-bin/fbidq.html?FBgn0039428) |
| [CG14265](http://flybase.org/cgi-bin/fbidq.html?FBgn0040393) | - | [CG14265](http://flybase.org/cgi-bin/fbidq.html?FBgn0040393) |
| [CG14266](http://flybase.org/cgi-bin/fbidq.html?FBgn0010294) | new glue 2 | [ng2](http://flybase.org/cgi-bin/fbidq.html?FBgn0010294) |
| [CG14356](http://flybase.org/cgi-bin/fbidq.html?FBgn0038207) | - | [CG14356](http://flybase.org/cgi-bin/fbidq.html?FBgn0038207) |
| [CG14430](http://flybase.org/cgi-bin/fbidq.html?FBgn0261284) | boudin | [bou](http://flybase.org/cgi-bin/fbidq.html?FBgn0261284) |
| [CG14434](http://flybase.org/cgi-bin/fbidq.html?FBgn0029915) | - | [CG14434](http://flybase.org/cgi-bin/fbidq.html?FBgn0029915) |
| [CG14483](http://flybase.org/cgi-bin/fbidq.html?FBgn0034248) | - | [CG14483](http://flybase.org/cgi-bin/fbidq.html?FBgn0034248) |
| [CG14543](http://flybase.org/cgi-bin/fbidq.html?FBgn0039404) | - | [CG14543](http://flybase.org/cgi-bin/fbidq.html?FBgn0039404) |
| [CG14577](http://flybase.org/cgi-bin/fbidq.html?FBgn0037110) | ORMDL | [ORMDL](http://flybase.org/cgi-bin/fbidq.html?FBgn0037110) |
| [CG14578](http://flybase.org/cgi-bin/fbidq.html?FBgn0260469) | - | [CR14578](http://flybase.org/cgi-bin/fbidq.html?FBgn0260469) |
| [CG1458](http://flybase.org/cgi-bin/fbidq.html?FBgn0062442) | - | [CG1458](http://flybase.org/cgi-bin/fbidq.html?FBgn0062442) |
| [CG14584](http://flybase.org/cgi-bin/fbidq.html?FBgn0031181) | Ionotropic receptor 20a | [Ir20a](http://flybase.org/cgi-bin/fbidq.html?FBgn0031181) |
| [CG14619](http://flybase.org/cgi-bin/fbidq.html?FBgn0031187) | - | [CG14619](http://flybase.org/cgi-bin/fbidq.html?FBgn0031187) |
| [CG14641](http://flybase.org/cgi-bin/fbidq.html?FBgn0037220) | - | [CG14641](http://flybase.org/cgi-bin/fbidq.html?FBgn0037220) |
| [CG14671](http://flybase.org/cgi-bin/fbidq.html?FBgn0037340) | - | [CG14671](http://flybase.org/cgi-bin/fbidq.html?FBgn0037340) |
| [CG14715](http://flybase.org/cgi-bin/fbidq.html?FBgn0037930) | - | [CG14715](http://flybase.org/cgi-bin/fbidq.html?FBgn0037930) |
| [CG14724](http://flybase.org/cgi-bin/fbidq.html?FBgn0019624) | Cytochrome c oxidase subunit Va | [CoVa](http://flybase.org/cgi-bin/fbidq.html?FBgn0019624) |
| [CG14812](http://flybase.org/cgi-bin/fbidq.html?FBgn0026090) | - | [CG14812](http://flybase.org/cgi-bin/fbidq.html?FBgn0026090) |
| [CG14818](http://flybase.org/cgi-bin/fbidq.html?FBgn0026088) | - | [CG14818](http://flybase.org/cgi-bin/fbidq.html?FBgn0026088) |
| [CG14852](http://flybase.org/cgi-bin/fbidq.html?FBgn0038242) | - | [CG14852](http://flybase.org/cgi-bin/fbidq.html?FBgn0038242) |
| [CG14865](http://flybase.org/cgi-bin/fbidq.html?FBgn0011476) | lethal (3) neo43 | [l(3)neo43](http://flybase.org/cgi-bin/fbidq.html?FBgn0011476) |
| [CG1488](http://flybase.org/cgi-bin/fbidq.html?FBgn0030367) | Cyp311a1 | [Cyp311a1](http://flybase.org/cgi-bin/fbidq.html?FBgn0030367) |
| [CG1489](http://flybase.org/cgi-bin/fbidq.html?FBgn0020369) | Pros45 | [Pros45](http://flybase.org/cgi-bin/fbidq.html?FBgn0020369) |
| [CG14903](http://flybase.org/cgi-bin/fbidq.html?FBgn0038446) | - | [CG14903](http://flybase.org/cgi-bin/fbidq.html?FBgn0038446) |
| [CG14933](http://flybase.org/cgi-bin/fbidq.html?FBgn0040968) | - | [CG14933](http://flybase.org/cgi-bin/fbidq.html?FBgn0040968) |
| [CG15000](http://flybase.org/cgi-bin/fbidq.html?FBgn0259986) | nab | [nab](http://flybase.org/cgi-bin/fbidq.html?FBgn0259986) |
| [CG15191](http://flybase.org/cgi-bin/fbidq.html?FBgn0000618) | enhancer of yellow 2 | [e(y)2](http://flybase.org/cgi-bin/fbidq.html?FBgn0000618) |
| [CG1527](http://flybase.org/cgi-bin/fbidq.html?FBgn0004404) | Ribosomal protein S14b | [RpS14b](http://flybase.org/cgi-bin/fbidq.html?FBgn0004404) |
| [CG1532](http://flybase.org/cgi-bin/fbidq.html?FBgn0031143) | - | [CG1532](http://flybase.org/cgi-bin/fbidq.html?FBgn0031143) |
| [CG15362](http://flybase.org/cgi-bin/fbidq.html?FBgn0031378) | - | [CG15362](http://flybase.org/cgi-bin/fbidq.html?FBgn0031378) |
| [CG15505](http://flybase.org/cgi-bin/fbidq.html?FBgn0039684) | Odorant-binding protein 99d | [Obp99d](http://flybase.org/cgi-bin/fbidq.html?FBgn0039684) |
| [CG15530](http://flybase.org/cgi-bin/fbidq.html?FBgn0039752) | - | [CG15530](http://flybase.org/cgi-bin/fbidq.html?FBgn0039752) |
| [CG15571](http://flybase.org/cgi-bin/fbidq.html?FBgn0029696) | - | [CG15571](http://flybase.org/cgi-bin/fbidq.html?FBgn0029696) |
| [CG1558](http://flybase.org/cgi-bin/fbidq.html?FBgn0027259) | kinetochore Mis12-Ndc80 network component 1 | [Kmn1](http://flybase.org/cgi-bin/fbidq.html?FBgn0027259) |
| [CG15626](http://flybase.org/cgi-bin/fbidq.html?FBgn0031635) | - | [CG15626](http://flybase.org/cgi-bin/fbidq.html?FBgn0031635) |
| [CG15701](http://flybase.org/cgi-bin/fbidq.html?FBgn0034095) | - | [CG15701](http://flybase.org/cgi-bin/fbidq.html?FBgn0034095) |
| [CG1572](http://flybase.org/cgi-bin/fbidq.html?FBgn0030309) | - | [CG1572](http://flybase.org/cgi-bin/fbidq.html?FBgn0030309) |
| [CG1577](http://flybase.org/cgi-bin/fbidq.html?FBgn0033208) | mitochondrial ribosomal protein L52 | [mRpL52](http://flybase.org/cgi-bin/fbidq.html?FBgn0033208) |
| [CG15800](http://flybase.org/cgi-bin/fbidq.html?FBgn0034904) | - | [CG15800](http://flybase.org/cgi-bin/fbidq.html?FBgn0034904) |
| [CG1660](http://flybase.org/cgi-bin/fbidq.html?FBgn0030480) | Tim9a | [Tim9a](http://flybase.org/cgi-bin/fbidq.html?FBgn0030480) |
| [CG1662](http://flybase.org/cgi-bin/fbidq.html?FBgn0030481) | - | [CG1662](http://flybase.org/cgi-bin/fbidq.html?FBgn0030481) |
| [CG16726](http://flybase.org/cgi-bin/fbidq.html?FBgn0053696) | - | [CG33696](http://flybase.org/cgi-bin/fbidq.html?FBgn0053696) |
| [CG16784](http://flybase.org/cgi-bin/fbidq.html?FBgn0003141) | purple | [pr](http://flybase.org/cgi-bin/fbidq.html?FBgn0003141) |
| [CG16817](http://flybase.org/cgi-bin/fbidq.html?FBgn0037728) | - | [CG16817](http://flybase.org/cgi-bin/fbidq.html?FBgn0037728) |
| [CG16916](http://flybase.org/cgi-bin/fbidq.html?FBgn0028686) | Rpt3 | [Rpt3](http://flybase.org/cgi-bin/fbidq.html?FBgn0028686) |
| [CG16941](http://flybase.org/cgi-bin/fbidq.html?FBgn0038464) | - | [CG16941](http://flybase.org/cgi-bin/fbidq.html?FBgn0038464) |
| [CG16982](http://flybase.org/cgi-bin/fbidq.html?FBgn0025638) | Roc1a | [Roc1a](http://flybase.org/cgi-bin/fbidq.html?FBgn0025638) |
| [CG17059](http://flybase.org/cgi-bin/fbidq.html?FBgn0040754) | - | [CG17059](http://flybase.org/cgi-bin/fbidq.html?FBgn0040754) |
| [CG17082](http://flybase.org/cgi-bin/fbidq.html?FBgn0039994) | - | [CG17082](http://flybase.org/cgi-bin/fbidq.html?FBgn0039994) |
| [CG17134](http://flybase.org/cgi-bin/fbidq.html?FBgn0032304) | - | [CG17134](http://flybase.org/cgi-bin/fbidq.html?FBgn0032304) |
| [CG17143](http://flybase.org/cgi-bin/fbidq.html?FBgn0035110) | thoc7 | [thoc7](http://flybase.org/cgi-bin/fbidq.html?FBgn0035110) |
| [CG17153](http://flybase.org/cgi-bin/fbidq.html?FBgn0036248) | - | [CG17153](http://flybase.org/cgi-bin/fbidq.html?FBgn0036248) |
| [CG17170](http://flybase.org/cgi-bin/fbidq.html?FBgn0003559) | suppressor of forked | [su(f)](http://flybase.org/cgi-bin/fbidq.html?FBgn0003559) |
| [CG17180](http://flybase.org/cgi-bin/fbidq.html?FBgn0035140) | - | [CG17180](http://flybase.org/cgi-bin/fbidq.html?FBgn0035140) |
| [CG17187](http://flybase.org/cgi-bin/fbidq.html?FBgn0037882) | - | [CG17187](http://flybase.org/cgi-bin/fbidq.html?FBgn0037882) |
| [CG17212](http://flybase.org/cgi-bin/fbidq.html?FBgn0032415) | rhomboid-6 | [rho-6](http://flybase.org/cgi-bin/fbidq.html?FBgn0032415) |
| [CG17294](http://flybase.org/cgi-bin/fbidq.html?FBgn0032032) | - | [CG17294](http://flybase.org/cgi-bin/fbidq.html?FBgn0032032) |
| [CG17327](http://flybase.org/cgi-bin/fbidq.html?FBgn0038107) | - | [CG17327](http://flybase.org/cgi-bin/fbidq.html?FBgn0038107) |
| [CG17333](http://flybase.org/cgi-bin/fbidq.html?FBgn0030239) | - | [CG17333](http://flybase.org/cgi-bin/fbidq.html?FBgn0030239) |
| [CG17337](http://flybase.org/cgi-bin/fbidq.html?FBgn0259979) | - | [CG17337](http://flybase.org/cgi-bin/fbidq.html?FBgn0259979) |
| [CG17358](http://flybase.org/cgi-bin/fbidq.html?FBgn0011290) | TBP-associated factor 12 | [Taf12](http://flybase.org/cgi-bin/fbidq.html?FBgn0011290) |
| [CG17420](http://flybase.org/cgi-bin/fbidq.html?FBgn0028697) | Ribosomal protein L15 | [RpL15](http://flybase.org/cgi-bin/fbidq.html?FBgn0028697) |
| [CG1749](http://flybase.org/cgi-bin/fbidq.html?FBgn0030305) | - | [CG1749](http://flybase.org/cgi-bin/fbidq.html?FBgn0030305) |
| [CG17556](http://flybase.org/cgi-bin/fbidq.html?FBgn0038462) | - | [CG17556](http://flybase.org/cgi-bin/fbidq.html?FBgn0038462) |
| [CG17691](http://flybase.org/cgi-bin/fbidq.html?FBgn0039993) | - | [CG17691](http://flybase.org/cgi-bin/fbidq.html?FBgn0039993) |
| [CG17721](http://flybase.org/cgi-bin/fbidq.html?FBgn0037885) | - | [CG17721](http://flybase.org/cgi-bin/fbidq.html?FBgn0037885) |
| [CG17737](http://flybase.org/cgi-bin/fbidq.html?FBgn0035423) | - | [CG17737](http://flybase.org/cgi-bin/fbidq.html?FBgn0035423) |
| [CG17776](http://flybase.org/cgi-bin/fbidq.html?FBgn0040899) | - | [CG17776](http://flybase.org/cgi-bin/fbidq.html?FBgn0040899) |
| [CG17912](http://flybase.org/cgi-bin/fbidq.html?FBgn0032600) | - | [CG17912](http://flybase.org/cgi-bin/fbidq.html?FBgn0032600) |
| [CG17921](http://flybase.org/cgi-bin/fbidq.html?FBgn0010228) | HMG protein Z | [HmgZ](http://flybase.org/cgi-bin/fbidq.html?FBgn0010228) |
| [CG17949](http://flybase.org/cgi-bin/fbidq.html?FBgn0061209) | His2B:CG17949 | [His2B:CG17949](http://flybase.org/cgi-bin/fbidq.html?FBgn0061209) |
| [CG17991](http://flybase.org/cgi-bin/fbidq.html?FBgn0039498) | - | [CG17991](http://flybase.org/cgi-bin/fbidq.html?FBgn0039498) |
| [CG1803](http://flybase.org/cgi-bin/fbidq.html?FBgn0030362) | regucalcin | [regucalcin](http://flybase.org/cgi-bin/fbidq.html?FBgn0030362) |
| [CG18210](http://flybase.org/cgi-bin/fbidq.html?FBgn0030636) | - | [CG18210](http://flybase.org/cgi-bin/fbidq.html?FBgn0030636) |
| [CG18212](http://flybase.org/cgi-bin/fbidq.html?FBgn0038535) | aluminum tubes | [alt](http://flybase.org/cgi-bin/fbidq.html?FBgn0038535) |
| [CG18319](http://flybase.org/cgi-bin/fbidq.html?FBgn0000173) | bendless | [ben](http://flybase.org/cgi-bin/fbidq.html?FBgn0000173) |
| [CG1836](http://flybase.org/cgi-bin/fbidq.html?FBgn0026777) | Rad23 | [Rad23](http://flybase.org/cgi-bin/fbidq.html?FBgn0026777) |
| [CG18495](http://flybase.org/cgi-bin/fbidq.html?FBgn0026781) | Proteasome alpha1 subunit | [Prosalpha1](http://flybase.org/cgi-bin/fbidq.html?FBgn0026781) |
| [CG18508](http://flybase.org/cgi-bin/fbidq.html?FBgn0028746) | - | [CG18508](http://flybase.org/cgi-bin/fbidq.html?FBgn0028746) |
| [CG18581](http://flybase.org/cgi-bin/fbidq.html?FBgn0036466) | - | [CG18581](http://flybase.org/cgi-bin/fbidq.html?FBgn0036466) |
| [CG18591](http://flybase.org/cgi-bin/fbidq.html?FBgn0031962) | - | [CG18591](http://flybase.org/cgi-bin/fbidq.html?FBgn0031962) |
| [CG18619](http://flybase.org/cgi-bin/fbidq.html?FBgn0032202) | - | [CG18619](http://flybase.org/cgi-bin/fbidq.html?FBgn0032202) |
| [CG18624](http://flybase.org/cgi-bin/fbidq.html?FBgn0029971) | - | [CG18624](http://flybase.org/cgi-bin/fbidq.html?FBgn0029971) |
| [CG1863](http://flybase.org/cgi-bin/fbidq.html?FBgn0261016) | - | [CG42600](http://flybase.org/cgi-bin/fbidq.html?FBgn0261016) |
| [CG18731](http://flybase.org/cgi-bin/fbidq.html?FBgn0042213) | - | [CG18731](http://flybase.org/cgi-bin/fbidq.html?FBgn0042213) |
| [CG18803](http://flybase.org/cgi-bin/fbidq.html?FBgn0019947) | Presenilin | [Psn](http://flybase.org/cgi-bin/fbidq.html?FBgn0019947) |
| [CG18815](http://flybase.org/cgi-bin/fbidq.html?FBgn0042138) | - | [CG18815](http://flybase.org/cgi-bin/fbidq.html?FBgn0042138) |
| [CG1935](http://flybase.org/cgi-bin/fbidq.html?FBgn0025820) | JTBR | [JTBR](http://flybase.org/cgi-bin/fbidq.html?FBgn0025820) |
| [CG1967](http://flybase.org/cgi-bin/fbidq.html?FBgn0030341) | p24-related-1 | [p24-1](http://flybase.org/cgi-bin/fbidq.html?FBgn0030341) |
| [CG2014](http://flybase.org/cgi-bin/fbidq.html?FBgn0039669) | - | [CG2014](http://flybase.org/cgi-bin/fbidq.html?FBgn0039669) |
| [CG2140](http://flybase.org/cgi-bin/fbidq.html?FBgn0033189) | Cyt-b5 | [Cyt-b5](http://flybase.org/cgi-bin/fbidq.html?FBgn0033189) |
| [CG2152](http://flybase.org/cgi-bin/fbidq.html?FBgn0086768) | Protein-L-isoaspartate (D-aspartate) O-methyltransferase | [Pcmt](http://flybase.org/cgi-bin/fbidq.html?FBgn0086768) |
| [CG2168](http://flybase.org/cgi-bin/fbidq.html?FBgn0017545) | Ribosomal protein S3A | [RpS3A](http://flybase.org/cgi-bin/fbidq.html?FBgn0017545) |
| [CG2202](http://flybase.org/cgi-bin/fbidq.html?FBgn0030240) | - | [CG2202](http://flybase.org/cgi-bin/fbidq.html?FBgn0030240) |
| [CG2217](http://flybase.org/cgi-bin/fbidq.html?FBgn0027544) | - | [CG2217](http://flybase.org/cgi-bin/fbidq.html?FBgn0027544) |
| [CG2264](http://flybase.org/cgi-bin/fbidq.html?FBgn0033478) | - | [CG2264](http://flybase.org/cgi-bin/fbidq.html?FBgn0033478) |
| [CG2291](http://flybase.org/cgi-bin/fbidq.html?FBgn0033279) | - | [CG2291](http://flybase.org/cgi-bin/fbidq.html?FBgn0033279) |
| [CG2358](http://flybase.org/cgi-bin/fbidq.html?FBgn0026567) | Spase 18/21-subunit | [Spase18-21](http://flybase.org/cgi-bin/fbidq.html?FBgn0026567) |
| [CG2444](http://flybase.org/cgi-bin/fbidq.html?FBgn0030326) | - | [CG2444](http://flybase.org/cgi-bin/fbidq.html?FBgn0030326) |
| [CG2668](http://flybase.org/cgi-bin/fbidq.html?FBgn0004181) | Protein ejaculatory bulb | [Peb](http://flybase.org/cgi-bin/fbidq.html?FBgn0004181) |
| [CG2720](http://flybase.org/cgi-bin/fbidq.html?FBgn0024352) | Hsp70/Hsp90 organizing protein homolog | [Hop](http://flybase.org/cgi-bin/fbidq.html?FBgn0024352) |
| [CG2789](http://flybase.org/cgi-bin/fbidq.html?FBgn0031263) | - | [CG2789](http://flybase.org/cgi-bin/fbidq.html?FBgn0031263) |
| [CG2811](http://flybase.org/cgi-bin/fbidq.html?FBgn0035082) | - | [CG2811](http://flybase.org/cgi-bin/fbidq.html?FBgn0035082) |
| [CG2852](http://flybase.org/cgi-bin/fbidq.html?FBgn0034753) | - | [CG2852](http://flybase.org/cgi-bin/fbidq.html?FBgn0034753) |
| [CG2859](http://flybase.org/cgi-bin/fbidq.html?FBgn0028398) | TBP-associated factor 10 | [Taf10](http://flybase.org/cgi-bin/fbidq.html?FBgn0028398) |
| [CG2862](http://flybase.org/cgi-bin/fbidq.html?FBgn0031459) | - | [CG2862](http://flybase.org/cgi-bin/fbidq.html?FBgn0031459) |
| [CG2934](http://flybase.org/cgi-bin/fbidq.html?FBgn0028665) | Vacuolar H[+]-ATPase C39 subunit | [VhaAC39](http://flybase.org/cgi-bin/fbidq.html?FBgn0028665) |
| [CG2968](http://flybase.org/cgi-bin/fbidq.html?FBgn0028342) | lethal (1) G0230 | [l(1)G0230](http://flybase.org/cgi-bin/fbidq.html?FBgn0028342) |
| [CG2983](http://flybase.org/cgi-bin/fbidq.html?FBgn0031472) | - | [CG2983](http://flybase.org/cgi-bin/fbidq.html?FBgn0031472) |
| [CG2991](http://flybase.org/cgi-bin/fbidq.html?FBgn0031474) | - | [CG2991](http://flybase.org/cgi-bin/fbidq.html?FBgn0031474) |
| [CG2998](http://flybase.org/cgi-bin/fbidq.html?FBgn0030136) | Ribosomal protein S28b | [RpS28b](http://flybase.org/cgi-bin/fbidq.html?FBgn0030136) |
| [CG30042](http://flybase.org/cgi-bin/fbidq.html?FBgn0050042) | Cuticular protein 49Ab | [Cpr49Ab](http://flybase.org/cgi-bin/fbidq.html?FBgn0050042) |
| [CG30100](http://flybase.org/cgi-bin/fbidq.html?FBgn0050100) | - | [CG30100](http://flybase.org/cgi-bin/fbidq.html?FBgn0050100) |
| [CG30104](http://flybase.org/cgi-bin/fbidq.html?FBgn0050104) | - | [CG30104](http://flybase.org/cgi-bin/fbidq.html?FBgn0050104) |
| [CG3019](http://flybase.org/cgi-bin/fbidq.html?FBgn0003638) | suppressor of white-apricot | [su(w[a])](http://flybase.org/cgi-bin/fbidq.html?FBgn0003638) |
| [CG30278](http://flybase.org/cgi-bin/fbidq.html?FBgn0050278) | - | [CG30278](http://flybase.org/cgi-bin/fbidq.html?FBgn0050278) |
| [CG30338](http://flybase.org/cgi-bin/fbidq.html?FBgn0050338) | - | [CG30338](http://flybase.org/cgi-bin/fbidq.html?FBgn0050338) |
| [CG30382](http://flybase.org/cgi-bin/fbidq.html?FBgn0050382) | - | [CG30382](http://flybase.org/cgi-bin/fbidq.html?FBgn0050382) |
| [CG3054](http://flybase.org/cgi-bin/fbidq.html?FBgn0022153) | lethal (2) k05819 | [l(2)k05819](http://flybase.org/cgi-bin/fbidq.html?FBgn0022153) |
| [CG31049](http://flybase.org/cgi-bin/fbidq.html?FBgn0259220) | Darkener of apricot | [Doa](http://flybase.org/cgi-bin/fbidq.html?FBgn0259220) |
| [CG31184](http://flybase.org/cgi-bin/fbidq.html?FBgn0051184) | - | [LSm3](http://flybase.org/cgi-bin/fbidq.html?FBgn0051184) |
| [CG31196](http://flybase.org/cgi-bin/fbidq.html?FBgn0020238) | 14-3-3epsilon | [14-3-3epsilon](http://flybase.org/cgi-bin/fbidq.html?FBgn0020238) |
| [CG31203](http://flybase.org/cgi-bin/fbidq.html?FBgn0259222) | - | [CG42322](http://flybase.org/cgi-bin/fbidq.html?FBgn0259222) |
| [CG31224](http://flybase.org/cgi-bin/fbidq.html?FBgn0051224) | - | [CG31224](http://flybase.org/cgi-bin/fbidq.html?FBgn0051224) |
| [CG31229](http://flybase.org/cgi-bin/fbidq.html?FBgn0051229) | - | [CG31229](http://flybase.org/cgi-bin/fbidq.html?FBgn0051229) |
| [CG31363](http://flybase.org/cgi-bin/fbidq.html?FBgn0051363) | Jupiter | [Jupiter](http://flybase.org/cgi-bin/fbidq.html?FBgn0051363) |
| [CG31370](http://flybase.org/cgi-bin/fbidq.html?FBgn0051370) | - | [CG31370](http://flybase.org/cgi-bin/fbidq.html?FBgn0051370) |
| [CG31450](http://flybase.org/cgi-bin/fbidq.html?FBgn0051450) | mitochondrial ribosomal protein S18A | [mRpS18A](http://flybase.org/cgi-bin/fbidq.html?FBgn0051450) |
| [CG31460](http://flybase.org/cgi-bin/fbidq.html?FBgn0051460) | - | [CG31460](http://flybase.org/cgi-bin/fbidq.html?FBgn0051460) |
| [CG31472](http://flybase.org/cgi-bin/fbidq.html?FBgn0051472) | - | [CG31472](http://flybase.org/cgi-bin/fbidq.html?FBgn0051472) |
| [CG31618](http://flybase.org/cgi-bin/fbidq.html?FBgn0051618) | His2A:CG31618 | [His2A:CG31618](http://flybase.org/cgi-bin/fbidq.html?FBgn0051618) |
| [CG31704](http://flybase.org/cgi-bin/fbidq.html?FBgn0051704) | - | [CG31704](http://flybase.org/cgi-bin/fbidq.html?FBgn0051704) |
| [CG31715](http://flybase.org/cgi-bin/fbidq.html?FBgn0051715) | - | [CG31715](http://flybase.org/cgi-bin/fbidq.html?FBgn0051715) |
| [CG31717](http://flybase.org/cgi-bin/fbidq.html?FBgn0051717) | - | [CG31717](http://flybase.org/cgi-bin/fbidq.html?FBgn0051717) |
| [CG3172](http://flybase.org/cgi-bin/fbidq.html?FBgn0038206) | twinfilin | [twf](http://flybase.org/cgi-bin/fbidq.html?FBgn0038206) |
| [CG31743](http://flybase.org/cgi-bin/fbidq.html?FBgn0032618) | - | [CG31743](http://flybase.org/cgi-bin/fbidq.html?FBgn0032618) |
| [CG31751](http://flybase.org/cgi-bin/fbidq.html?FBgn0086909) | - | [CG31751](http://flybase.org/cgi-bin/fbidq.html?FBgn0086909) |
| [CG31810](http://flybase.org/cgi-bin/fbidq.html?FBgn0051810) | - | [CG31810](http://flybase.org/cgi-bin/fbidq.html?FBgn0051810) |
| [CG3186](http://flybase.org/cgi-bin/fbidq.html?FBgn0034967) | eIF-5A | [eIF-5A](http://flybase.org/cgi-bin/fbidq.html?FBgn0034967) |
| [CG32041](http://flybase.org/cgi-bin/fbidq.html?FBgn0001223) | Heat shock protein 22 | [Hsp22](http://flybase.org/cgi-bin/fbidq.html?FBgn0001223) |
| [CG32068](http://flybase.org/cgi-bin/fbidq.html?FBgn0052068) | - | [CG32068](http://flybase.org/cgi-bin/fbidq.html?FBgn0052068) |
| [CG32071](http://flybase.org/cgi-bin/fbidq.html?FBgn0052071) | - | [CG32071](http://flybase.org/cgi-bin/fbidq.html?FBgn0052071) |
| [CG3214](http://flybase.org/cgi-bin/fbidq.html?FBgn0031436) | - | [CG3214](http://flybase.org/cgi-bin/fbidq.html?FBgn0031436) |
| [CG32147](http://flybase.org/cgi-bin/fbidq.html?FBgn0047178) | - | [CG32147](http://flybase.org/cgi-bin/fbidq.html?FBgn0047178) |
| [CG32163](http://flybase.org/cgi-bin/fbidq.html?FBgn0052163) | - | [CG32163](http://flybase.org/cgi-bin/fbidq.html?FBgn0052163) |
| [CG32198](http://flybase.org/cgi-bin/fbidq.html?FBgn0052198) | - | [CG32198](http://flybase.org/cgi-bin/fbidq.html?FBgn0052198) |
| [CG32282](http://flybase.org/cgi-bin/fbidq.html?FBgn0052282) | drosomycin-4 | [dro4](http://flybase.org/cgi-bin/fbidq.html?FBgn0052282) |
| [CG32353](http://flybase.org/cgi-bin/fbidq.html?FBgn0052353) | - | [CG32353](http://flybase.org/cgi-bin/fbidq.html?FBgn0052353) |
| [CG32441](http://flybase.org/cgi-bin/fbidq.html?FBgn0052441) | - | [CG32441](http://flybase.org/cgi-bin/fbidq.html?FBgn0052441) |
| [CG32446](http://flybase.org/cgi-bin/fbidq.html?FBgn0052446) | - | [Atox1](http://flybase.org/cgi-bin/fbidq.html?FBgn0052446) |
| [CG32448](http://flybase.org/cgi-bin/fbidq.html?FBgn0052448) | - | [CG32448](http://flybase.org/cgi-bin/fbidq.html?FBgn0052448) |
| [CG32458](http://flybase.org/cgi-bin/fbidq.html?FBgn0052458) | - | [CG32458](http://flybase.org/cgi-bin/fbidq.html?FBgn0052458) |
| [CG32494](http://flybase.org/cgi-bin/fbidq.html?FBgn0052494) | - | [CG32494](http://flybase.org/cgi-bin/fbidq.html?FBgn0052494) |
| [CG32581](http://flybase.org/cgi-bin/fbidq.html?FBgn0052581) | - | [CG32581](http://flybase.org/cgi-bin/fbidq.html?FBgn0052581) |
| [CG32602](http://flybase.org/cgi-bin/fbidq.html?FBgn0052602) | Mucin 12Ea | [Muc12Ea](http://flybase.org/cgi-bin/fbidq.html?FBgn0052602) |
| [CG32640](http://flybase.org/cgi-bin/fbidq.html?FBgn0052640) | - | [CG32640](http://flybase.org/cgi-bin/fbidq.html?FBgn0052640) |
| [CG32954](http://flybase.org/cgi-bin/fbidq.html?FBgn0000056) | Adh-related | [Adhr](http://flybase.org/cgi-bin/fbidq.html?FBgn0000056) |
| [CG33102](http://flybase.org/cgi-bin/fbidq.html?FBgn0042711) | Hex-t1 | [Hex-t1](http://flybase.org/cgi-bin/fbidq.html?FBgn0042711) |
| [CG33105](http://flybase.org/cgi-bin/fbidq.html?FBgn0053105) | p24-related-2 | [p24-2](http://flybase.org/cgi-bin/fbidq.html?FBgn0053105) |
| [CG33127](http://flybase.org/cgi-bin/fbidq.html?FBgn0053127) | - | [CG33127](http://flybase.org/cgi-bin/fbidq.html?FBgn0053127) |
| [CG3314](http://flybase.org/cgi-bin/fbidq.html?FBgn0014026) | Ribosomal protein L7A | [RpL7A](http://flybase.org/cgi-bin/fbidq.html?FBgn0014026) |
| [CG33198](http://flybase.org/cgi-bin/fbidq.html?FBgn0053198) | presenilin enhancer | [pen-2](http://flybase.org/cgi-bin/fbidq.html?FBgn0053198) |
| [CG33228](http://flybase.org/cgi-bin/fbidq.html?FBgn0261373) | - | [CG33228](http://flybase.org/cgi-bin/fbidq.html?FBgn0261373) |
| [CG3348](http://flybase.org/cgi-bin/fbidq.html?FBgn0040609) | - | [CG3348](http://flybase.org/cgi-bin/fbidq.html?FBgn0040609) |
| [CG33493](http://flybase.org/cgi-bin/fbidq.html?FBgn0053493) | - | [CG33493](http://flybase.org/cgi-bin/fbidq.html?FBgn0053493) |
| [CG3402](http://flybase.org/cgi-bin/fbidq.html?FBgn0035148) | - | [CG3402](http://flybase.org/cgi-bin/fbidq.html?FBgn0035148) |
| [CG3495](http://flybase.org/cgi-bin/fbidq.html?FBgn0034794) | GDP-4-keto-6-deoxy-D-mannose 3,5-epimerase/4-reductase | [Gmer](http://flybase.org/cgi-bin/fbidq.html?FBgn0034794) |
| [CG3560](http://flybase.org/cgi-bin/fbidq.html?FBgn0030733) | - | [CG3560](http://flybase.org/cgi-bin/fbidq.html?FBgn0030733) |
| [CG3609](http://flybase.org/cgi-bin/fbidq.html?FBgn0031418) | - | [CG3609](http://flybase.org/cgi-bin/fbidq.html?FBgn0031418) |
| [CG3683](http://flybase.org/cgi-bin/fbidq.html?FBgn0035046) | - | [CG3683](http://flybase.org/cgi-bin/fbidq.html?FBgn0035046) |
| [CG3717](http://flybase.org/cgi-bin/fbidq.html?FBgn0013432) | bcn92 | [bcn92](http://flybase.org/cgi-bin/fbidq.html?FBgn0013432) |
| [CG3773](http://flybase.org/cgi-bin/fbidq.html?FBgn0038692) | - | [CG3773](http://flybase.org/cgi-bin/fbidq.html?FBgn0038692) |
| [CG3832](http://flybase.org/cgi-bin/fbidq.html?FBgn0019948) | Peptidylglycine-alpha-hydroxylating monooxygenase | [Phm](http://flybase.org/cgi-bin/fbidq.html?FBgn0019948) |
| [CG3887](http://flybase.org/cgi-bin/fbidq.html?FBgn0031670) | - | [CG3887](http://flybase.org/cgi-bin/fbidq.html?FBgn0031670) |
| [CG3949](http://flybase.org/cgi-bin/fbidq.html?FBgn0015393) | hoi-polloi | [hoip](http://flybase.org/cgi-bin/fbidq.html?FBgn0015393) |
| [CG3959](http://flybase.org/cgi-bin/fbidq.html?FBgn0011207) | pelota | [pelo](http://flybase.org/cgi-bin/fbidq.html?FBgn0011207) |
| [CG3997](http://flybase.org/cgi-bin/fbidq.html?FBgn0023170) | Ribosomal protein L39 | [RpL39](http://flybase.org/cgi-bin/fbidq.html?FBgn0023170) |
| [CG40042](http://flybase.org/cgi-bin/fbidq.html?FBgn0058042) | - | [CG40042](http://flybase.org/cgi-bin/fbidq.html?FBgn0058042) |
| [CG40196](http://flybase.org/cgi-bin/fbidq.html?FBgn0058196) | - | [CG40196](http://flybase.org/cgi-bin/fbidq.html?FBgn0058196) |
| [CG40218](http://flybase.org/cgi-bin/fbidq.html?FBgn0043842) | Yeti | [Yeti](http://flybase.org/cgi-bin/fbidq.html?FBgn0043842) |
| [CG40293](http://flybase.org/cgi-bin/fbidq.html?FBgn0046692) | Ste20-like kinase | [Stlk](http://flybase.org/cgi-bin/fbidq.html?FBgn0046692) |
| [CG40451](http://flybase.org/cgi-bin/fbidq.html?FBgn0067318) | - | [CG40451](http://flybase.org/cgi-bin/fbidq.html?FBgn0067318) |
| [CG4097](http://flybase.org/cgi-bin/fbidq.html?FBgn0002284) | Proteasome 26kD subunit | [Pros26](http://flybase.org/cgi-bin/fbidq.html?FBgn0002284) |
| [CG4108](http://flybase.org/cgi-bin/fbidq.html?FBgn0036805) | Chmp1 | [Chmp1](http://flybase.org/cgi-bin/fbidq.html?FBgn0036805) |
| [CG41128](http://flybase.org/cgi-bin/fbidq.html?FBgn0069923) | - | [CG41128](http://flybase.org/cgi-bin/fbidq.html?FBgn0069923) |
| [CG4173](http://flybase.org/cgi-bin/fbidq.html?FBgn0014029) | Septin-2 | [Sep2](http://flybase.org/cgi-bin/fbidq.html?FBgn0014029) |
| [CG4196](http://flybase.org/cgi-bin/fbidq.html?FBgn0260659) | - | [CG42542](http://flybase.org/cgi-bin/fbidq.html?FBgn0260659) |
| [CG4247](http://flybase.org/cgi-bin/fbidq.html?FBgn0038307) | mitochondrial ribosomal protein S10 | [mRpS10](http://flybase.org/cgi-bin/fbidq.html?FBgn0038307) |
| [CG4306](http://flybase.org/cgi-bin/fbidq.html?FBgn0036787) | - | [CG4306](http://flybase.org/cgi-bin/fbidq.html?FBgn0036787) |
| [CG4400](http://flybase.org/cgi-bin/fbidq.html?FBgn0030434) | - | [CG4400](http://flybase.org/cgi-bin/fbidq.html?FBgn0030434) |
| [CG4463](http://flybase.org/cgi-bin/fbidq.html?FBgn0001224) | Heat shock protein 23 | [Hsp23](http://flybase.org/cgi-bin/fbidq.html?FBgn0001224) |
| [CG4494](http://flybase.org/cgi-bin/fbidq.html?FBgn0026170) | smt3 | [smt3](http://flybase.org/cgi-bin/fbidq.html?FBgn0026170) |
| [CG4594](http://flybase.org/cgi-bin/fbidq.html?FBgn0032161) | - | [CG4594](http://flybase.org/cgi-bin/fbidq.html?FBgn0032161) |
| [CG4598](http://flybase.org/cgi-bin/fbidq.html?FBgn0032160) | - | [CG4598](http://flybase.org/cgi-bin/fbidq.html?FBgn0032160) |
| [CG4600](http://flybase.org/cgi-bin/fbidq.html?FBgn0040064) | yippee interacting protein 2 | [yip2](http://flybase.org/cgi-bin/fbidq.html?FBgn0040064) |
| [CG4605](http://flybase.org/cgi-bin/fbidq.html?FBgn0023415) | Accessory gland-specific peptide 32CD | [Acp32CD](http://flybase.org/cgi-bin/fbidq.html?FBgn0023415) |
| [CG4634](http://flybase.org/cgi-bin/fbidq.html?FBgn0016687) | Nucleosome remodeling factor - 38kD | [Nurf-38](http://flybase.org/cgi-bin/fbidq.html?FBgn0016687) |
| [CG4665](http://flybase.org/cgi-bin/fbidq.html?FBgn0035964) | Dihydropteridine reductase | [Dhpr](http://flybase.org/cgi-bin/fbidq.html?FBgn0035964) |
| [CG4830](http://flybase.org/cgi-bin/fbidq.html?FBgn0037996) | - | [CG4830](http://flybase.org/cgi-bin/fbidq.html?FBgn0037996) |
| [CG4872](http://flybase.org/cgi-bin/fbidq.html?FBgn0030799) | - | [CG4872](http://flybase.org/cgi-bin/fbidq.html?FBgn0030799) |
| [CG4878](http://flybase.org/cgi-bin/fbidq.html?FBgn0034237) | eIF3-S9 | [eIF3-S9](http://flybase.org/cgi-bin/fbidq.html?FBgn0034237) |
| [CG4946](http://flybase.org/cgi-bin/fbidq.html?FBgn0259482) | - | [Mob3](http://flybase.org/cgi-bin/fbidq.html?FBgn0259482) |
| [CG4957](http://flybase.org/cgi-bin/fbidq.html?FBgn0032205) | - | [CG4957](http://flybase.org/cgi-bin/fbidq.html?FBgn0032205) |
| [CG5021](http://flybase.org/cgi-bin/fbidq.html?FBgn0035944) | - | [CG5021](http://flybase.org/cgi-bin/fbidq.html?FBgn0035944) |
| [CG5039](http://flybase.org/cgi-bin/fbidq.html?FBgn0039356) | - | [CG5039](http://flybase.org/cgi-bin/fbidq.html?FBgn0039356) |
| [CG5050](http://flybase.org/cgi-bin/fbidq.html?FBgn0032637) | - | [CG5050](http://flybase.org/cgi-bin/fbidq.html?FBgn0032637) |
| [CG5067](http://flybase.org/cgi-bin/fbidq.html?FBgn0028386) | capicua | [cic](http://flybase.org/cgi-bin/fbidq.html?FBgn0028386) |
| [CG5118](http://flybase.org/cgi-bin/fbidq.html?FBgn0031317) | - | [CG5118](http://flybase.org/cgi-bin/fbidq.html?FBgn0031317) |
| [CG5134](http://flybase.org/cgi-bin/fbidq.html?FBgn0260401) | Mediator complex subunit 9 | [MED9](http://flybase.org/cgi-bin/fbidq.html?FBgn0260401) |
| [CG5184](http://flybase.org/cgi-bin/fbidq.html?FBgn0038474) | mitochondrial ribosomal protein S11 | [mRpS11](http://flybase.org/cgi-bin/fbidq.html?FBgn0038474) |
| [CG5189](http://flybase.org/cgi-bin/fbidq.html?FBgn0034350) | - | [CG5189](http://flybase.org/cgi-bin/fbidq.html?FBgn0034350) |
| [CG5214](http://flybase.org/cgi-bin/fbidq.html?FBgn0037891) | - | [CG5214](http://flybase.org/cgi-bin/fbidq.html?FBgn0037891) |
| [CG5245](http://flybase.org/cgi-bin/fbidq.html?FBgn0038047) | - | [CG5245](http://flybase.org/cgi-bin/fbidq.html?FBgn0038047) |
| [CG5249](http://flybase.org/cgi-bin/fbidq.html?FBgn0035625) | Blimp-1 | [Blimp-1](http://flybase.org/cgi-bin/fbidq.html?FBgn0035625) |
| [CG5258](http://flybase.org/cgi-bin/fbidq.html?FBgn0029148) | NHP2 | [NHP2](http://flybase.org/cgi-bin/fbidq.html?FBgn0029148) |
| [CG5266](http://flybase.org/cgi-bin/fbidq.html?FBgn0086134) | Proteasome 25kD subunit | [Pros25](http://flybase.org/cgi-bin/fbidq.html?FBgn0086134) |
| [CG5276](http://flybase.org/cgi-bin/fbidq.html?FBgn0037900) | - | [CG5276](http://flybase.org/cgi-bin/fbidq.html?FBgn0037900) |
| [CG5290](http://flybase.org/cgi-bin/fbidq.html?FBgn0036772) | - | [CG5290](http://flybase.org/cgi-bin/fbidq.html?FBgn0036772) |
| [CG5323](http://flybase.org/cgi-bin/fbidq.html?FBgn0034362) | - | [CG5323](http://flybase.org/cgi-bin/fbidq.html?FBgn0034362) |
| [CG5335](http://flybase.org/cgi-bin/fbidq.html?FBgn0034365) | - | [CG5335](http://flybase.org/cgi-bin/fbidq.html?FBgn0034365) |
| [CG5338](http://flybase.org/cgi-bin/fbidq.html?FBgn0039129) | Ribosomal protein S19b | [RpS19b](http://flybase.org/cgi-bin/fbidq.html?FBgn0039129) |
| [CG5352](http://flybase.org/cgi-bin/fbidq.html?FBgn0010083) | Small ribonucleoprotein particle protein B | [SmB](http://flybase.org/cgi-bin/fbidq.html?FBgn0010083) |
| [CG5371](http://flybase.org/cgi-bin/fbidq.html?FBgn0011703) | Ribonucleoside diphosphate reductase large subunit | [RnrL](http://flybase.org/cgi-bin/fbidq.html?FBgn0011703) |
| [CG5385](http://flybase.org/cgi-bin/fbidq.html?FBgn0032215) | - | [CG5385](http://flybase.org/cgi-bin/fbidq.html?FBgn0032215) |
| [CG5442](http://flybase.org/cgi-bin/fbidq.html?FBgn0040286) | SC35 | [SC35](http://flybase.org/cgi-bin/fbidq.html?FBgn0040286) |
| [CG5474](http://flybase.org/cgi-bin/fbidq.html?FBgn0011016) | Signal sequence receptor beta | [SsRbeta](http://flybase.org/cgi-bin/fbidq.html?FBgn0011016) |
| [CG5498](http://flybase.org/cgi-bin/fbidq.html?FBgn0027565) | - | [CG5498](http://flybase.org/cgi-bin/fbidq.html?FBgn0027565) |
| [CG5509](http://flybase.org/cgi-bin/fbidq.html?FBgn0038054) | - | [CG5509](http://flybase.org/cgi-bin/fbidq.html?FBgn0038054) |
| [CG5520](http://flybase.org/cgi-bin/fbidq.html?FBgn0039562) | Glycoprotein 93 | [Gp93](http://flybase.org/cgi-bin/fbidq.html?FBgn0039562) |
| [CG5677](http://flybase.org/cgi-bin/fbidq.html?FBgn0039172) | Spase 22/23-subunit | [Spase22-23](http://flybase.org/cgi-bin/fbidq.html?FBgn0039172) |
| [CG5703](http://flybase.org/cgi-bin/fbidq.html?FBgn0030853) | - | [CG5703](http://flybase.org/cgi-bin/fbidq.html?FBgn0030853) |
| [CG5773](http://flybase.org/cgi-bin/fbidq.html?FBgn0034290) | - | [CG5773](http://flybase.org/cgi-bin/fbidq.html?FBgn0034290) |
| [CG5851](http://flybase.org/cgi-bin/fbidq.html?FBgn0028992) | sds22 | [sds22](http://flybase.org/cgi-bin/fbidq.html?FBgn0028992) |
| [CG5864](http://flybase.org/cgi-bin/fbidq.html?FBgn0039132) | AP-1sigma | [AP-1sigma](http://flybase.org/cgi-bin/fbidq.html?FBgn0039132) |
| [CG5885](http://flybase.org/cgi-bin/fbidq.html?FBgn0025700) | - | [CG5885](http://flybase.org/cgi-bin/fbidq.html?FBgn0025700) |
| [CG5915](http://flybase.org/cgi-bin/fbidq.html?FBgn0015795) | Rab-protein 7 | [Rab7](http://flybase.org/cgi-bin/fbidq.html?FBgn0015795) |
| [CG5920](http://flybase.org/cgi-bin/fbidq.html?FBgn0004867) | string of pearls | [sop](http://flybase.org/cgi-bin/fbidq.html?FBgn0004867) |
| [CG5972](http://flybase.org/cgi-bin/fbidq.html?FBgn0031781) | Arc-p20 | [Arc-p20](http://flybase.org/cgi-bin/fbidq.html?FBgn0031781) |
| [CG5989](http://flybase.org/cgi-bin/fbidq.html?FBgn0017429) | - | [CG5989](http://flybase.org/cgi-bin/fbidq.html?FBgn0017429) |
| [CG6000](http://flybase.org/cgi-bin/fbidq.html?FBgn0039145) | - | [CG6000](http://flybase.org/cgi-bin/fbidq.html?FBgn0039145) |
| [CG6008](http://flybase.org/cgi-bin/fbidq.html?FBgn0027785) | NP15.6 | [NP15.6](http://flybase.org/cgi-bin/fbidq.html?FBgn0027785) |
| [CG6011](http://flybase.org/cgi-bin/fbidq.html?FBgn0027784) | Prp18 | [Prp18](http://flybase.org/cgi-bin/fbidq.html?FBgn0027784) |
| [CG6030](http://flybase.org/cgi-bin/fbidq.html?FBgn0016120) | ATP synthase, subunit d | [ATPsyn-d](http://flybase.org/cgi-bin/fbidq.html?FBgn0016120) |
| [CG6034](http://flybase.org/cgi-bin/fbidq.html?FBgn0036750) | - | [CG6034](http://flybase.org/cgi-bin/fbidq.html?FBgn0036750) |
| [CG6058](http://flybase.org/cgi-bin/fbidq.html?FBgn0000064) | Aldolase | [Ald](http://flybase.org/cgi-bin/fbidq.html?FBgn0000064) |
| [CG6202](http://flybase.org/cgi-bin/fbidq.html?FBgn0019925) | Surfeit 4 | [Surf4](http://flybase.org/cgi-bin/fbidq.html?FBgn0019925) |
| [CG6272](http://flybase.org/cgi-bin/fbidq.html?FBgn0036126) | - | [CG6272](http://flybase.org/cgi-bin/fbidq.html?FBgn0036126) |
| [CG6459](http://flybase.org/cgi-bin/fbidq.html?FBgn0034259) | - | [CG6459](http://flybase.org/cgi-bin/fbidq.html?FBgn0034259) |
| [CG6510](http://flybase.org/cgi-bin/fbidq.html?FBgn0010409) | Ribosomal protein L18A | [RpL18A](http://flybase.org/cgi-bin/fbidq.html?FBgn0010409) |
| [CG6523](http://flybase.org/cgi-bin/fbidq.html?FBgn0032509) | - | [CG6523](http://flybase.org/cgi-bin/fbidq.html?FBgn0032509) |
| [CG6567](http://flybase.org/cgi-bin/fbidq.html?FBgn0037842) | - | [CG6567](http://flybase.org/cgi-bin/fbidq.html?FBgn0037842) |
| [CG6647](http://flybase.org/cgi-bin/fbidq.html?FBgn0004363) | porin | [porin](http://flybase.org/cgi-bin/fbidq.html?FBgn0004363) |
| [CG6666](http://flybase.org/cgi-bin/fbidq.html?FBgn0037873) | Succinate dehydrogenase C | [SdhC](http://flybase.org/cgi-bin/fbidq.html?FBgn0037873) |
| [CG6686](http://flybase.org/cgi-bin/fbidq.html?FBgn0032388) | - | [CG6686](http://flybase.org/cgi-bin/fbidq.html?FBgn0032388) |
| [CG6719](http://flybase.org/cgi-bin/fbidq.html?FBgn0037893) | - | [CG6719](http://flybase.org/cgi-bin/fbidq.html?FBgn0037893) |
| [CG6764](http://flybase.org/cgi-bin/fbidq.html?FBgn0037899) | - | [CG6764](http://flybase.org/cgi-bin/fbidq.html?FBgn0037899) |
| [CG6773](http://flybase.org/cgi-bin/fbidq.html?FBgn0024509) | sec13 | [sec13](http://flybase.org/cgi-bin/fbidq.html?FBgn0024509) |
| [CG6779](http://flybase.org/cgi-bin/fbidq.html?FBgn0002622) | Ribosomal protein S3 | [RpS3](http://flybase.org/cgi-bin/fbidq.html?FBgn0002622) |
| [CG6910](http://flybase.org/cgi-bin/fbidq.html?FBgn0036262) | - | [CG6910](http://flybase.org/cgi-bin/fbidq.html?FBgn0036262) |
| [CG7013](http://flybase.org/cgi-bin/fbidq.html?FBgn0027095) | Mesencephalic astrocyte-derived neurotrophic factor | [Manf](http://flybase.org/cgi-bin/fbidq.html?FBgn0027095) |
| [CG7038](http://flybase.org/cgi-bin/fbidq.html?FBgn0029718) | mitochondrial ribosomal protein L30 | [mRpL30](http://flybase.org/cgi-bin/fbidq.html?FBgn0029718) |
| [CG7048](http://flybase.org/cgi-bin/fbidq.html?FBgn0038976) | - | [CG7048](http://flybase.org/cgi-bin/fbidq.html?FBgn0038976) |
| [CG7085](http://flybase.org/cgi-bin/fbidq.html?FBgn0010704) | lethal (2) s5379 | [l(2)s5379](http://flybase.org/cgi-bin/fbidq.html?FBgn0010704) |
| [CG7123](http://flybase.org/cgi-bin/fbidq.html?FBgn0002527) | Laminin B1 | [LanB1](http://flybase.org/cgi-bin/fbidq.html?FBgn0002527) |
| [CG7188](http://flybase.org/cgi-bin/fbidq.html?FBgn0035871) | - | [CG7188](http://flybase.org/cgi-bin/fbidq.html?FBgn0035871) |
| [CG7224](http://flybase.org/cgi-bin/fbidq.html?FBgn0031971) | - | [CG7224](http://flybase.org/cgi-bin/fbidq.html?FBgn0031971) |
| [CG7301](http://flybase.org/cgi-bin/fbidq.html?FBgn0053547) | Rim | [Rim](http://flybase.org/cgi-bin/fbidq.html?FBgn0053547) |
| [CG7322](http://flybase.org/cgi-bin/fbidq.html?FBgn0030968) | - | [CG7322](http://flybase.org/cgi-bin/fbidq.html?FBgn0030968) |
| [CG7424](http://flybase.org/cgi-bin/fbidq.html?FBgn0031980) | Ribosomal protein L36A | [RpL36A](http://flybase.org/cgi-bin/fbidq.html?FBgn0031980) |
| [CG7452](http://flybase.org/cgi-bin/fbidq.html?FBgn0035540) | Syntaxin 17 | [Syx17](http://flybase.org/cgi-bin/fbidq.html?FBgn0035540) |
| [CG7523](http://flybase.org/cgi-bin/fbidq.html?FBgn0038533) | - | [CG7523](http://flybase.org/cgi-bin/fbidq.html?FBgn0038533) |
| [CG7584](http://flybase.org/cgi-bin/fbidq.html?FBgn0039682) | Odorant-binding protein 99c | [Obp99c](http://flybase.org/cgi-bin/fbidq.html?FBgn0039682) |
| [CG7587](http://flybase.org/cgi-bin/fbidq.html?FBgn0038523) | - | [CG7587](http://flybase.org/cgi-bin/fbidq.html?FBgn0038523) |
| [CG7596](http://flybase.org/cgi-bin/fbidq.html?FBgn0003375) | Salivary gland secretion 5 | [Sgs5](http://flybase.org/cgi-bin/fbidq.html?FBgn0003375) |
| [CG7622](http://flybase.org/cgi-bin/fbidq.html?FBgn0002579) | Ribosomal protein L36 | [RpL36](http://flybase.org/cgi-bin/fbidq.html?FBgn0002579) |
| [CG7625](http://flybase.org/cgi-bin/fbidq.html?FBgn0028663) | VhaM9.7-2 | [VhaM9.7-2](http://flybase.org/cgi-bin/fbidq.html?FBgn0028663) |
| [CG7655](http://flybase.org/cgi-bin/fbidq.html?FBgn0038536) | - | [CG7655](http://flybase.org/cgi-bin/fbidq.html?FBgn0038536) |
| [CG7662](http://flybase.org/cgi-bin/fbidq.html?FBgn0039269) | veli | [veli](http://flybase.org/cgi-bin/fbidq.html?FBgn0039269) |
| [CG7668](http://flybase.org/cgi-bin/fbidq.html?FBgn0036929) | - | [CG7668](http://flybase.org/cgi-bin/fbidq.html?FBgn0036929) |
| [CG7685](http://flybase.org/cgi-bin/fbidq.html?FBgn0038619) | - | [CG7685](http://flybase.org/cgi-bin/fbidq.html?FBgn0038619) |
| [CG7770](http://flybase.org/cgi-bin/fbidq.html?FBgn0036918) | - | [CG7770](http://flybase.org/cgi-bin/fbidq.html?FBgn0036918) |
| [CG7787](http://flybase.org/cgi-bin/fbidq.html?FBgn0032020) | - | [CG7787](http://flybase.org/cgi-bin/fbidq.html?FBgn0032020) |
| [CG7800](http://flybase.org/cgi-bin/fbidq.html?FBgn0037552) | - | [CG7800](http://flybase.org/cgi-bin/fbidq.html?FBgn0037552) |
| [CG7823](http://flybase.org/cgi-bin/fbidq.html?FBgn0036921) | RhoGDI | [RhoGDI](http://flybase.org/cgi-bin/fbidq.html?FBgn0036921) |
| [CG7834](http://flybase.org/cgi-bin/fbidq.html?FBgn0039697) | - | [CG7834](http://flybase.org/cgi-bin/fbidq.html?FBgn0039697) |
| [CG7865](http://flybase.org/cgi-bin/fbidq.html?FBgn0033050) | PNGase | [PNGase](http://flybase.org/cgi-bin/fbidq.html?FBgn0033050) |
| [CG7891](http://flybase.org/cgi-bin/fbidq.html?FBgn0037551) | novel GTPase indispensable for equal segregation of chromosomes | [Gie](http://flybase.org/cgi-bin/fbidq.html?FBgn0037551) |
| [CG7911](http://flybase.org/cgi-bin/fbidq.html?FBgn0039735) | - | [CG7911](http://flybase.org/cgi-bin/fbidq.html?FBgn0039735) |
| [CG7925](http://flybase.org/cgi-bin/fbidq.html?FBgn0003714) | technical knockout | [tko](http://flybase.org/cgi-bin/fbidq.html?FBgn0003714) |
| [CG7940](http://flybase.org/cgi-bin/fbidq.html?FBgn0038576) | Actin-related protein 5 | [Arp5](http://flybase.org/cgi-bin/fbidq.html?FBgn0038576) |
| [CG7961](http://flybase.org/cgi-bin/fbidq.html?FBgn0025725) | alpha-coatomer protein | [alphaCop](http://flybase.org/cgi-bin/fbidq.html?FBgn0025725) |
| [CG7993](http://flybase.org/cgi-bin/fbidq.html?FBgn0038585) | - | [CG7993](http://flybase.org/cgi-bin/fbidq.html?FBgn0038585) |
| [CG8112](http://flybase.org/cgi-bin/fbidq.html?FBgn0037612) | - | [CG8112](http://flybase.org/cgi-bin/fbidq.html?FBgn0037612) |
| [CG8119](http://flybase.org/cgi-bin/fbidq.html?FBgn0030664) | - | [CG8119](http://flybase.org/cgi-bin/fbidq.html?FBgn0030664) |
| [CG8159](http://flybase.org/cgi-bin/fbidq.html?FBgn0037619) | - | [CG8159](http://flybase.org/cgi-bin/fbidq.html?FBgn0037619) |
| [CG8161](http://flybase.org/cgi-bin/fbidq.html?FBgn0014022) | Rlb1 | [Rlb1](http://flybase.org/cgi-bin/fbidq.html?FBgn0014022) |
| [CG8186](http://flybase.org/cgi-bin/fbidq.html?FBgn0022097) | Vha36 | [Vha36](http://flybase.org/cgi-bin/fbidq.html?FBgn0022097) |
| [CG8251](http://flybase.org/cgi-bin/fbidq.html?FBgn0003074) | Phosphoglucose isomerase | [Pgi](http://flybase.org/cgi-bin/fbidq.html?FBgn0003074) |
| [CG8264](http://flybase.org/cgi-bin/fbidq.html?FBgn0004856) | Bx42 | [Bx42](http://flybase.org/cgi-bin/fbidq.html?FBgn0004856) |
| [CG8268](http://flybase.org/cgi-bin/fbidq.html?FBgn0035827) | Srp9 | [Srp9](http://flybase.org/cgi-bin/fbidq.html?FBgn0035827) |
| [CG8272](http://flybase.org/cgi-bin/fbidq.html?FBgn0033337) | - | [CG8272](http://flybase.org/cgi-bin/fbidq.html?FBgn0033337) |
| [CG8315](http://flybase.org/cgi-bin/fbidq.html?FBgn0034058) | - | [CG8315](http://flybase.org/cgi-bin/fbidq.html?FBgn0034058) |
| [CG8331](http://flybase.org/cgi-bin/fbidq.html?FBgn0033906) | - | [CG8331](http://flybase.org/cgi-bin/fbidq.html?FBgn0033906) |
| [CG8332](http://flybase.org/cgi-bin/fbidq.html?FBgn0034138) | Ribosomal protein S15 | [RpS15](http://flybase.org/cgi-bin/fbidq.html?FBgn0034138) |
| [CG8372](http://flybase.org/cgi-bin/fbidq.html?FBgn0032000) | - | [CG8372](http://flybase.org/cgi-bin/fbidq.html?FBgn0032000) |
| [CG8386](http://flybase.org/cgi-bin/fbidq.html?FBgn0034061) | - | [CG8386](http://flybase.org/cgi-bin/fbidq.html?FBgn0034061) |
| [CG8396](http://flybase.org/cgi-bin/fbidq.html?FBgn0015299) | Single stranded-binding protein c31A | [Ssb-c31a](http://flybase.org/cgi-bin/fbidq.html?FBgn0015299) |
| [CG8409](http://flybase.org/cgi-bin/fbidq.html?FBgn0003607) | Suppressor of variegation 205 | [Su(var)205](http://flybase.org/cgi-bin/fbidq.html?FBgn0003607) |
| [CG8472](http://flybase.org/cgi-bin/fbidq.html?FBgn0000253) | Calmodulin | [Cam](http://flybase.org/cgi-bin/fbidq.html?FBgn0000253) |
| [CG8498](http://flybase.org/cgi-bin/fbidq.html?FBgn0031992) | - | [CG8498](http://flybase.org/cgi-bin/fbidq.html?FBgn0031992) |
| [CG8615](http://flybase.org/cgi-bin/fbidq.html?FBgn0035753) | Ribosomal protein L18 | [RpL18](http://flybase.org/cgi-bin/fbidq.html?FBgn0035753) |
| [CG8674](http://flybase.org/cgi-bin/fbidq.html?FBgn0021856) | lethal (2) k14505 | [l(2)k14505](http://flybase.org/cgi-bin/fbidq.html?FBgn0021856) |
| [CG8743](http://flybase.org/cgi-bin/fbidq.html?FBgn0036904) | transient receptor potential mucolipin | [trpml](http://flybase.org/cgi-bin/fbidq.html?FBgn0036904) |
| [CG8781](http://flybase.org/cgi-bin/fbidq.html?FBgn0033378) | tsunagi | [tsu](http://flybase.org/cgi-bin/fbidq.html?FBgn0033378) |
| [CG8844](http://flybase.org/cgi-bin/fbidq.html?FBgn0021967) | Pdsw | [Pdsw](http://flybase.org/cgi-bin/fbidq.html?FBgn0021967) |
| [CG8846](http://flybase.org/cgi-bin/fbidq.html?FBgn0022073) | Thor | [Thor](http://flybase.org/cgi-bin/fbidq.html?FBgn0022073) |
| [CG8882](http://flybase.org/cgi-bin/fbidq.html?FBgn0015834) | Trip1 | [Trip1](http://flybase.org/cgi-bin/fbidq.html?FBgn0015834) |
| [CG8891](http://flybase.org/cgi-bin/fbidq.html?FBgn0031663) | - | [CG8891](http://flybase.org/cgi-bin/fbidq.html?FBgn0031663) |
| [CG8905](http://flybase.org/cgi-bin/fbidq.html?FBgn0010213) | Superoxide dismutase 2 (Mn) | [Sod2](http://flybase.org/cgi-bin/fbidq.html?FBgn0010213) |
| [CG8922](http://flybase.org/cgi-bin/fbidq.html?FBgn0002590) | Ribosomal protein S5a | [RpS5a](http://flybase.org/cgi-bin/fbidq.html?FBgn0002590) |
| [CG8945](http://flybase.org/cgi-bin/fbidq.html?FBgn0030815) | - | [CG8945](http://flybase.org/cgi-bin/fbidq.html?FBgn0030815) |
| [CG8993](http://flybase.org/cgi-bin/fbidq.html?FBgn0035334) | - | [CG8993](http://flybase.org/cgi-bin/fbidq.html?FBgn0035334) |
| [CG8998](http://flybase.org/cgi-bin/fbidq.html?FBgn0044020) | Roc2 | [Roc2](http://flybase.org/cgi-bin/fbidq.html?FBgn0044020) |
| [CG9035](http://flybase.org/cgi-bin/fbidq.html?FBgn0021795) | Translocon-associated protein delta | [Tapdelta](http://flybase.org/cgi-bin/fbidq.html?FBgn0021795) |
| [CG9040](http://flybase.org/cgi-bin/fbidq.html?FBgn0036394) | - | [CG9040](http://flybase.org/cgi-bin/fbidq.html?FBgn0036394) |
| [CG9067](http://flybase.org/cgi-bin/fbidq.html?FBgn0033605) | - | [CG9067](http://flybase.org/cgi-bin/fbidq.html?FBgn0033605) |
| [CG9070](http://flybase.org/cgi-bin/fbidq.html?FBgn0086519) | Cuticular protein 47Eg | [Cpr47Eg](http://flybase.org/cgi-bin/fbidq.html?FBgn0086519) |
| [CG9075](http://flybase.org/cgi-bin/fbidq.html?FBgn0001942) | Eukaryotic initiation factor 4a | [eIF-4a](http://flybase.org/cgi-bin/fbidq.html?FBgn0001942) |
| [CG9075](http://flybase.org/cgi-bin/fbidq.html?FBgn0001942) | Eukaryotic initiation factor 4a | [eIF-4a](http://flybase.org/cgi-bin/fbidq.html?FBgn0001942) |
| [CG9080](http://flybase.org/cgi-bin/fbidq.html?FBgn0033593) | - | [CG9080](http://flybase.org/cgi-bin/fbidq.html?FBgn0033593) |
| [CG9091](http://flybase.org/cgi-bin/fbidq.html?FBgn0030616) | Ribosomal protein L37a | [RpL37a](http://flybase.org/cgi-bin/fbidq.html?FBgn0030616) |
| [CG9296](http://flybase.org/cgi-bin/fbidq.html?FBgn0032059) | Prenyl-binding protein | [PrBP](http://flybase.org/cgi-bin/fbidq.html?FBgn0032059) |
| [CG9327](http://flybase.org/cgi-bin/fbidq.html?FBgn0261394) | Proteasome 29kD subunit | [Pros29](http://flybase.org/cgi-bin/fbidq.html?FBgn0261394) |
| [CG9336](http://flybase.org/cgi-bin/fbidq.html?FBgn0032897) | - | [CG9336](http://flybase.org/cgi-bin/fbidq.html?FBgn0032897) |
| [CG9388](http://flybase.org/cgi-bin/fbidq.html?FBgn0024833) | AP-47 | [AP-47](http://flybase.org/cgi-bin/fbidq.html?FBgn0024833) |
| [CG9538](http://flybase.org/cgi-bin/fbidq.html?FBgn0015010) | Antigen 5-related | [Ag5r](http://flybase.org/cgi-bin/fbidq.html?FBgn0015010) |
| [CG9548](http://flybase.org/cgi-bin/fbidq.html?FBgn0031822) | - | [CG9548](http://flybase.org/cgi-bin/fbidq.html?FBgn0031822) |
| [CG9553](http://flybase.org/cgi-bin/fbidq.html?FBgn0000308) | chickadee | [chic](http://flybase.org/cgi-bin/fbidq.html?FBgn0000308) |
| [CG9603](http://flybase.org/cgi-bin/fbidq.html?FBgn0040529) | - | [CG9603](http://flybase.org/cgi-bin/fbidq.html?FBgn0040529) |
| [CG9617](http://flybase.org/cgi-bin/fbidq.html?FBgn0260939) | - | [Sgt1](http://flybase.org/cgi-bin/fbidq.html?FBgn0260939) |
| [CG9648](http://flybase.org/cgi-bin/fbidq.html?FBgn0017578) | Max | [Max](http://flybase.org/cgi-bin/fbidq.html?FBgn0017578) |
| [CG9667](http://flybase.org/cgi-bin/fbidq.html?FBgn0037550) | - | [CG9667](http://flybase.org/cgi-bin/fbidq.html?FBgn0037550) |
| [CG9670](http://flybase.org/cgi-bin/fbidq.html?FBgn0028380) | falten | [fal](http://flybase.org/cgi-bin/fbidq.html?FBgn0028380) |
| [CG9705](http://flybase.org/cgi-bin/fbidq.html?FBgn0036661) | - | [CG9705](http://flybase.org/cgi-bin/fbidq.html?FBgn0036661) |
| [CG9734](http://flybase.org/cgi-bin/fbidq.html?FBgn0027657) | globin 1 | [glob1](http://flybase.org/cgi-bin/fbidq.html?FBgn0027657) |
| [CG9762](http://flybase.org/cgi-bin/fbidq.html?FBgn0011455) | lethal (3) neo18 | [l(3)neo18](http://flybase.org/cgi-bin/fbidq.html?FBgn0011455) |
| [CG9769](http://flybase.org/cgi-bin/fbidq.html?FBgn0037270) | - | [CG9769](http://flybase.org/cgi-bin/fbidq.html?FBgn0037270) |
| [CG9779](http://flybase.org/cgi-bin/fbidq.html?FBgn0037231) | - | [CG9779](http://flybase.org/cgi-bin/fbidq.html?FBgn0037231) |
| [CG9829](http://flybase.org/cgi-bin/fbidq.html?FBgn0086371) | poly | [poly](http://flybase.org/cgi-bin/fbidq.html?FBgn0086371) |
| [CG9866](http://flybase.org/cgi-bin/fbidq.html?FBgn0031420) | - | [CG9866](http://flybase.org/cgi-bin/fbidq.html?FBgn0031420) |
| [CG9881](http://flybase.org/cgi-bin/fbidq.html?FBgn0031437) | p16-ARC | [p16-ARC](http://flybase.org/cgi-bin/fbidq.html?FBgn0031437) |
| [CG9890](http://flybase.org/cgi-bin/fbidq.html?FBgn0034814) | - | [CG9890](http://flybase.org/cgi-bin/fbidq.html?FBgn0034814) |
| [CG9916](http://flybase.org/cgi-bin/fbidq.html?FBgn0004432) | Cyclophilin 1 | [Cyp1](http://flybase.org/cgi-bin/fbidq.html?FBgn0004432) |
| [CG9947](http://flybase.org/cgi-bin/fbidq.html?FBgn0030752) | - | [CG9947](http://flybase.org/cgi-bin/fbidq.html?FBgn0030752) |
| [CG9954](http://flybase.org/cgi-bin/fbidq.html?FBgn0034534) | maf-S | [maf-S](http://flybase.org/cgi-bin/fbidq.html?FBgn0034534) |
| [CR31356](http://flybase.org/cgi-bin/fbidq.html?FBgn0051356) | transfer RNA:CR31356 | [tRNA:CR31356](http://flybase.org/cgi-bin/fbidq.html?FBgn0051356) |
| [CR32162](http://flybase.org/cgi-bin/fbidq.html?FBgn0041721) | snRNA:U12:73B | [snRNA:U12:73B](http://flybase.org/cgi-bin/fbidq.html?FBgn0041721) |
| [CR32665](http://flybase.org/cgi-bin/fbidq.html?FBgn0019660) | RNA on the X 2 | [roX2](http://flybase.org/cgi-bin/fbidq.html?FBgn0019660) |
| [CR32864](http://flybase.org/cgi-bin/fbidq.html?FBgn0000003) | RNA 7SL | [7SLRNA](http://flybase.org/cgi-bin/fbidq.html?FBgn0000003) |
| [CR32894](http://flybase.org/cgi-bin/fbidq.html?FBgn0063383) | snoRNA:U29:54Ec | [snoRNA:U29:54Ec](http://flybase.org/cgi-bin/fbidq.html?FBgn0063383) |
