# Supplementary material for: Chronic activation of the epithelial immune system of the fruit fly's salivary glands has a negative effect on organismal growth and induces a peculiar set of target genes
Source: BMC Genomics. 2010 Apr 26;11:265. doi: 10.1186/1471-2164-11-265 (PMC2874812; doi:10.1186/1471-2164-11-265)
Supplement: Additional file 4 — Drosophila Salivary glands: Genes downregulated following IMD-pathway activation. This file contains a list of those genes whose expression in the salivary glands is downregulated significantly following activation of the IMD-pathway. [file 1471-2164-11-265-S4.DOC]

**Drosophila Salivary glands: Genes downregulated following IMD-activation**

| **SUBMITTED ID** | **NAME** | **SYMBOL** |
| --- | --- | --- |
| [CG10001](http://flybase.org/cgi-bin/fbidq.html?FBgn0039595) | Allatostatin Receptor 2 | [AR-2](http://flybase.org/cgi-bin/fbidq.html?FBgn0039595) |
| [CG10031](http://flybase.org/cgi-bin/fbidq.html?FBgn0031563) | - | [CG10031](http://flybase.org/cgi-bin/fbidq.html?FBgn0031563) |
| [CG10039](http://flybase.org/cgi-bin/fbidq.html?FBgn0031581) | - | [CG10039](http://flybase.org/cgi-bin/fbidq.html?FBgn0031581) |
| [CG10073](http://flybase.org/cgi-bin/fbidq.html?FBgn0034440) | - | [CG10073](http://flybase.org/cgi-bin/fbidq.html?FBgn0034440) |
| [CG10089](http://flybase.org/cgi-bin/fbidq.html?FBgn0036369) | - | [CG10089](http://flybase.org/cgi-bin/fbidq.html?FBgn0036369) |
| [CG10109](http://flybase.org/cgi-bin/fbidq.html?FBgn0001332) | Lobe | [L](http://flybase.org/cgi-bin/fbidq.html?FBgn0001332) |
| [CG10160](http://flybase.org/cgi-bin/fbidq.html?FBgn0001258) | Ecdysone-inducible gene L3 | [ImpL3](http://flybase.org/cgi-bin/fbidq.html?FBgn0001258) |
| [CG10170](http://flybase.org/cgi-bin/fbidq.html?FBgn0039085) | - | [CG10170](http://flybase.org/cgi-bin/fbidq.html?FBgn0039085) |
| [CG10205](http://flybase.org/cgi-bin/fbidq.html?FBgn0033970) | - | [CG10205](http://flybase.org/cgi-bin/fbidq.html?FBgn0033970) |
| [CG10215](http://flybase.org/cgi-bin/fbidq.html?FBgn0028434) | Ercc1 | [Ercc1](http://flybase.org/cgi-bin/fbidq.html?FBgn0028434) |
| [CG10225](http://flybase.org/cgi-bin/fbidq.html?FBgn0039110) | - | [CG10225](http://flybase.org/cgi-bin/fbidq.html?FBgn0039110) |
| [CG10248](http://flybase.org/cgi-bin/fbidq.html?FBgn0013772) | Cytochrome P450-6a8 | [Cyp6a8](http://flybase.org/cgi-bin/fbidq.html?FBgn0013772) |
| [CG10297](http://flybase.org/cgi-bin/fbidq.html?FBgn0020765) | Acp65Aa | [Acp65Aa](http://flybase.org/cgi-bin/fbidq.html?FBgn0020765) |
| [CG1031](http://flybase.org/cgi-bin/fbidq.html?FBgn0015568) | alpha-Esterase-1 | [alpha-Est1](http://flybase.org/cgi-bin/fbidq.html?FBgn0015568) |
| [CG10334](http://flybase.org/cgi-bin/fbidq.html?FBgn0005672) | spitz | [spi](http://flybase.org/cgi-bin/fbidq.html?FBgn0005672) |
| [CG10339](http://flybase.org/cgi-bin/fbidq.html?FBgn0034972) | - | [CG10339](http://flybase.org/cgi-bin/fbidq.html?FBgn0034972) |
| [CG10372](http://flybase.org/cgi-bin/fbidq.html?FBgn0025608) | Fas-associated factor | [Faf](http://flybase.org/cgi-bin/fbidq.html?FBgn0025608) |
| [CG10391](http://flybase.org/cgi-bin/fbidq.html?FBgn0032693) | Cyp310a1 | [Cyp310a1](http://flybase.org/cgi-bin/fbidq.html?FBgn0032693) |
| [CG10480](http://flybase.org/cgi-bin/fbidq.html?FBgn0002638) | Bj1 protein | [Bj1](http://flybase.org/cgi-bin/fbidq.html?FBgn0002638) |
| [CG10537](http://flybase.org/cgi-bin/fbidq.html?FBgn0004244) | Resistant to dieldrin | [Rdl](http://flybase.org/cgi-bin/fbidq.html?FBgn0004244) |
| [CG10590](http://flybase.org/cgi-bin/fbidq.html?FBgn0035622) | - | [CG10590](http://flybase.org/cgi-bin/fbidq.html?FBgn0035622) |
| [CG10650](http://flybase.org/cgi-bin/fbidq.html?FBgn0046302) | - | [CG10650](http://flybase.org/cgi-bin/fbidq.html?FBgn0046302) |
| [CG1066](http://flybase.org/cgi-bin/fbidq.html?FBgn0003383) | Shaker cognate b | [Shab](http://flybase.org/cgi-bin/fbidq.html?FBgn0003383) |
| [CG10670](http://flybase.org/cgi-bin/fbidq.html?FBgn0027914) | XPG-like endonuclease | [Gen](http://flybase.org/cgi-bin/fbidq.html?FBgn0027914) |
| [CG1072](http://flybase.org/cgi-bin/fbidq.html?FBgn0013751) | Arrowhead | [Awh](http://flybase.org/cgi-bin/fbidq.html?FBgn0013751) |
| [CG10726](http://flybase.org/cgi-bin/fbidq.html?FBgn0014127) | barren | [barr](http://flybase.org/cgi-bin/fbidq.html?FBgn0014127) |
| [CG10743](http://flybase.org/cgi-bin/fbidq.html?FBgn0036376) | - | [CG10743](http://flybase.org/cgi-bin/fbidq.html?FBgn0036376) |
| [CG10823](http://flybase.org/cgi-bin/fbidq.html?FBgn0038880) | SIFamide receptor | [SIFR](http://flybase.org/cgi-bin/fbidq.html?FBgn0038880) |
| [CG10851](http://flybase.org/cgi-bin/fbidq.html?FBgn0004587) | B52 | [B52](http://flybase.org/cgi-bin/fbidq.html?FBgn0004587) |
| [CG1091](http://flybase.org/cgi-bin/fbidq.html?FBgn0037470) | - | [CG1091](http://flybase.org/cgi-bin/fbidq.html?FBgn0037470) |
| [CG10952](http://flybase.org/cgi-bin/fbidq.html?FBgn0000535) | ether a go-go | [eag](http://flybase.org/cgi-bin/fbidq.html?FBgn0000535) |
| [CG10953](http://flybase.org/cgi-bin/fbidq.html?FBgn0034204) | - | [CG10953](http://flybase.org/cgi-bin/fbidq.html?FBgn0034204) |
| [CG1107](http://flybase.org/cgi-bin/fbidq.html?FBgn0037218) | auxillin | [aux](http://flybase.org/cgi-bin/fbidq.html?FBgn0037218) |
| [CG11081](http://flybase.org/cgi-bin/fbidq.html?FBgn0025741) | plexin A | [plexA](http://flybase.org/cgi-bin/fbidq.html?FBgn0025741) |
| [CG11100](http://flybase.org/cgi-bin/fbidq.html?FBgn0037207) | Mes2 | [Mes2](http://flybase.org/cgi-bin/fbidq.html?FBgn0037207) |
| [CG11131](http://flybase.org/cgi-bin/fbidq.html?FBgn0037204) | - | [CG11131](http://flybase.org/cgi-bin/fbidq.html?FBgn0037204) |
| [CG11211](http://flybase.org/cgi-bin/fbidq.html?FBgn0033067) | - | [CG11211](http://flybase.org/cgi-bin/fbidq.html?FBgn0033067) |
| [CG11295](http://flybase.org/cgi-bin/fbidq.html?FBgn0013548) | lethal-(2)-denticleless | [l(2)dtl](http://flybase.org/cgi-bin/fbidq.html?FBgn0013548) |
| [CG11298](http://flybase.org/cgi-bin/fbidq.html?FBgn0034721) | - | [CG11298](http://flybase.org/cgi-bin/fbidq.html?FBgn0034721) |
| [CG11320](http://flybase.org/cgi-bin/fbidq.html?FBgn0031837) | - | [CG11320](http://flybase.org/cgi-bin/fbidq.html?FBgn0031837) |
| [CG11325](http://flybase.org/cgi-bin/fbidq.html?FBgn0025595) | Gonadotropin-releasing hormone receptor | [GRHR](http://flybase.org/cgi-bin/fbidq.html?FBgn0025595) |
| [CG1136](http://flybase.org/cgi-bin/fbidq.html?FBgn0035490) | - | [CG1136](http://flybase.org/cgi-bin/fbidq.html?FBgn0035490) |
| [CG11374](http://flybase.org/cgi-bin/fbidq.html?FBgn0031214) | - | [CG11374](http://flybase.org/cgi-bin/fbidq.html?FBgn0031214) |
| [CG11382](http://flybase.org/cgi-bin/fbidq.html?FBgn0040367) | - | [CG11382](http://flybase.org/cgi-bin/fbidq.html?FBgn0040367) |
| [CG11388](http://flybase.org/cgi-bin/fbidq.html?FBgn0034959) | - | [CG11388](http://flybase.org/cgi-bin/fbidq.html?FBgn0034959) |
| [CG11638](http://flybase.org/cgi-bin/fbidq.html?FBgn0040351) | - | [CG11638](http://flybase.org/cgi-bin/fbidq.html?FBgn0040351) |
| [CG11639](http://flybase.org/cgi-bin/fbidq.html?FBgn0040338) | TfIIA-S-2 | [TfIIA-S-2](http://flybase.org/cgi-bin/fbidq.html?FBgn0040338) |
| [CG1165](http://flybase.org/cgi-bin/fbidq.html?FBgn0004430) | Lysozyme S | [LysS](http://flybase.org/cgi-bin/fbidq.html?FBgn0004430) |
| [CG11732](http://flybase.org/cgi-bin/fbidq.html?FBgn0037589) | Odorant-binding protein 85a | [Obp85a](http://flybase.org/cgi-bin/fbidq.html?FBgn0037589) |
| [CG11765](http://flybase.org/cgi-bin/fbidq.html?FBgn0033520) | Peroxiredoxin 2540 | [Prx2540-1](http://flybase.org/cgi-bin/fbidq.html?FBgn0033520) |
| [CG11778](http://flybase.org/cgi-bin/fbidq.html?FBgn0033371) | - | [CG11778](http://flybase.org/cgi-bin/fbidq.html?FBgn0033371) |
| [CG11799](http://flybase.org/cgi-bin/fbidq.html?FBgn0036134) | Mnf | [Mnf](http://flybase.org/cgi-bin/fbidq.html?FBgn0036134) |
| [CG11820](http://flybase.org/cgi-bin/fbidq.html?FBgn0039270) | - | [CG11820](http://flybase.org/cgi-bin/fbidq.html?FBgn0039270) |
| [CG11849](http://flybase.org/cgi-bin/fbidq.html?FBgn0039286) | distal antenna | [dan](http://flybase.org/cgi-bin/fbidq.html?FBgn0039286) |
| [CG11861](http://flybase.org/cgi-bin/fbidq.html?FBgn0261268) | cullin 3 | [cul-3](http://flybase.org/cgi-bin/fbidq.html?FBgn0261268) |
| [CG11864](http://flybase.org/cgi-bin/fbidq.html?FBgn0028944) | - | [CG11864](http://flybase.org/cgi-bin/fbidq.html?FBgn0028944) |
| [CG11878](http://flybase.org/cgi-bin/fbidq.html?FBgn0039310) | - | [CG11878](http://flybase.org/cgi-bin/fbidq.html?FBgn0039310) |
| [CG11908](http://flybase.org/cgi-bin/fbidq.html?FBgn0027376) | rha | [rha](http://flybase.org/cgi-bin/fbidq.html?FBgn0027376) |
| [CG11911](http://flybase.org/cgi-bin/fbidq.html?FBgn0031249) | - | [CG11911](http://flybase.org/cgi-bin/fbidq.html?FBgn0031249) |
| [CG11992](http://flybase.org/cgi-bin/fbidq.html?FBgn0014018) | Relish | [Rel](http://flybase.org/cgi-bin/fbidq.html?FBgn0014018) |
| [CG12048](http://flybase.org/cgi-bin/fbidq.html?FBgn0039675) | pickpocket 21 | [ppk21](http://flybase.org/cgi-bin/fbidq.html?FBgn0039675) |
| [CG12072](http://flybase.org/cgi-bin/fbidq.html?FBgn0011739) | warts | [wts](http://flybase.org/cgi-bin/fbidq.html?FBgn0011739) |
| [CG1208](http://flybase.org/cgi-bin/fbidq.html?FBgn0037386) | - | [CG1208](http://flybase.org/cgi-bin/fbidq.html?FBgn0037386) |
| [CG12082](http://flybase.org/cgi-bin/fbidq.html?FBgn0035402) | - | [CG12082](http://flybase.org/cgi-bin/fbidq.html?FBgn0035402) |
| [CG12132](http://flybase.org/cgi-bin/fbidq.html?FBgn0040236) | c11.1 | [c11.1](http://flybase.org/cgi-bin/fbidq.html?FBgn0040236) |
| [CG12159](http://flybase.org/cgi-bin/fbidq.html?FBgn0033232) | - | [CG12159](http://flybase.org/cgi-bin/fbidq.html?FBgn0033232) |
| [CG12169](http://flybase.org/cgi-bin/fbidq.html?FBgn0035143) | Ppm1 | [Ppm1](http://flybase.org/cgi-bin/fbidq.html?FBgn0035143) |
| [CG12224](http://flybase.org/cgi-bin/fbidq.html?FBgn0037974) | - | [CG12224](http://flybase.org/cgi-bin/fbidq.html?FBgn0037974) |
| [CG12262](http://flybase.org/cgi-bin/fbidq.html?FBgn0035811) | - | [CG12262](http://flybase.org/cgi-bin/fbidq.html?FBgn0035811) |
| [CG12290](http://flybase.org/cgi-bin/fbidq.html?FBgn0039419) | - | [CG12290](http://flybase.org/cgi-bin/fbidq.html?FBgn0039419) |
| [CG12317](http://flybase.org/cgi-bin/fbidq.html?FBgn0028425) | JhI-21 | [JhI-21](http://flybase.org/cgi-bin/fbidq.html?FBgn0028425) |
| [CG12345](http://flybase.org/cgi-bin/fbidq.html?FBgn0015323) | VAChT | [VAChT](http://flybase.org/cgi-bin/fbidq.html?FBgn0015323) |
| [CG12385](http://flybase.org/cgi-bin/fbidq.html?FBgn0011555) | thetaTrypsin | [thetaTry](http://flybase.org/cgi-bin/fbidq.html?FBgn0011555) |
| [CG1239](http://flybase.org/cgi-bin/fbidq.html?FBgn0037368) | - | [CG1239](http://flybase.org/cgi-bin/fbidq.html?FBgn0037368) |
| [CG12607](http://flybase.org/cgi-bin/fbidq.html?FBgn0035545) | - | [CG12607](http://flybase.org/cgi-bin/fbidq.html?FBgn0035545) |
| [CG12781](http://flybase.org/cgi-bin/fbidq.html?FBgn0034797) | nahoda | [nahoda](http://flybase.org/cgi-bin/fbidq.html?FBgn0034797) |
| [CG12878](http://flybase.org/cgi-bin/fbidq.html?FBgn0045862) | barentsz | [btz](http://flybase.org/cgi-bin/fbidq.html?FBgn0045862) |
| [CG12907](http://flybase.org/cgi-bin/fbidq.html?FBgn0250840) | - | [CG12907](http://flybase.org/cgi-bin/fbidq.html?FBgn0250840) |
| [CG12920](http://flybase.org/cgi-bin/fbidq.html?FBgn0033481) | - | [CG12920](http://flybase.org/cgi-bin/fbidq.html?FBgn0033481) |
| [CG12971](http://flybase.org/cgi-bin/fbidq.html?FBgn0037078) | - | [CG12971](http://flybase.org/cgi-bin/fbidq.html?FBgn0037078) |
| [CG13044](http://flybase.org/cgi-bin/fbidq.html?FBgn0036599) | - | [CG13044](http://flybase.org/cgi-bin/fbidq.html?FBgn0036599) |
| [CG13049](http://flybase.org/cgi-bin/fbidq.html?FBgn0036592) | - | [CG13049](http://flybase.org/cgi-bin/fbidq.html?FBgn0036592) |
| [CG13055](http://flybase.org/cgi-bin/fbidq.html?FBgn0036583) | - | [CG13055](http://flybase.org/cgi-bin/fbidq.html?FBgn0036583) |
| [CG13069](http://flybase.org/cgi-bin/fbidq.html?FBgn0040798) | - | [CG13069](http://flybase.org/cgi-bin/fbidq.html?FBgn0040798) |
| [CG13073](http://flybase.org/cgi-bin/fbidq.html?FBgn0036577) | - | [CG13073](http://flybase.org/cgi-bin/fbidq.html?FBgn0036577) |
| [CG13081](http://flybase.org/cgi-bin/fbidq.html?FBgn0032804) | - | [CG13081](http://flybase.org/cgi-bin/fbidq.html?FBgn0032804) |
| [CG13111](http://flybase.org/cgi-bin/fbidq.html?FBgn0085395) | - | [CG34366](http://flybase.org/cgi-bin/fbidq.html?FBgn0085395) |
| [CG13120](http://flybase.org/cgi-bin/fbidq.html?FBgn0032142) | - | [CG13120](http://flybase.org/cgi-bin/fbidq.html?FBgn0032142) |
| [CG13131](http://flybase.org/cgi-bin/fbidq.html?FBgn0032175) | - | [CG13131](http://flybase.org/cgi-bin/fbidq.html?FBgn0032175) |
| [CG13133](http://flybase.org/cgi-bin/fbidq.html?FBgn0032181) | - | [CG13133](http://flybase.org/cgi-bin/fbidq.html?FBgn0032181) |
| [CG13135](http://flybase.org/cgi-bin/fbidq.html?FBgn0032184) | - | [CG13135](http://flybase.org/cgi-bin/fbidq.html?FBgn0032184) |
| [CG13137](http://flybase.org/cgi-bin/fbidq.html?FBgn0032188) | - | [CG13137](http://flybase.org/cgi-bin/fbidq.html?FBgn0032188) |
| [CG13215](http://flybase.org/cgi-bin/fbidq.html?FBgn0033592) | - | [CG13215](http://flybase.org/cgi-bin/fbidq.html?FBgn0033592) |
| [CG13330](http://flybase.org/cgi-bin/fbidq.html?FBgn0033848) | - | [CG13330](http://flybase.org/cgi-bin/fbidq.html?FBgn0033848) |
| [CG13373](http://flybase.org/cgi-bin/fbidq.html?FBgn0029522) | - | [CG13373](http://flybase.org/cgi-bin/fbidq.html?FBgn0029522) |
| [CG13475](http://flybase.org/cgi-bin/fbidq.html?FBgn0040318) | HGTX | [HGTX](http://flybase.org/cgi-bin/fbidq.html?FBgn0040318) |
| [CG13490](http://flybase.org/cgi-bin/fbidq.html?FBgn0085398) | - | [CG34369](http://flybase.org/cgi-bin/fbidq.html?FBgn0085398) |
| [CG13502](http://flybase.org/cgi-bin/fbidq.html?FBgn0034692) | - | [CG13502](http://flybase.org/cgi-bin/fbidq.html?FBgn0034692) |
| [CG13512](http://flybase.org/cgi-bin/fbidq.html?FBgn0260768) | - | [CG42566](http://flybase.org/cgi-bin/fbidq.html?FBgn0260768) |
| [CG13702](http://flybase.org/cgi-bin/fbidq.html?FBgn0036789) | allatostatin C receptor 2 | [AlCR2](http://flybase.org/cgi-bin/fbidq.html?FBgn0036789) |
| [CG13723](http://flybase.org/cgi-bin/fbidq.html?FBgn0036705) | - | [CG13723](http://flybase.org/cgi-bin/fbidq.html?FBgn0036705) |
| [CG13737](http://flybase.org/cgi-bin/fbidq.html?FBgn0036382) | - | [CG13737](http://flybase.org/cgi-bin/fbidq.html?FBgn0036382) |
| [CG13746](http://flybase.org/cgi-bin/fbidq.html?FBgn0033341) | MrgBP | [MrgBP](http://flybase.org/cgi-bin/fbidq.html?FBgn0033341) |
| [CG13759](http://flybase.org/cgi-bin/fbidq.html?FBgn0040376) | - | [CG13759](http://flybase.org/cgi-bin/fbidq.html?FBgn0040376) |
| [CG13794](http://flybase.org/cgi-bin/fbidq.html?FBgn0031936) | - | [CG13794](http://flybase.org/cgi-bin/fbidq.html?FBgn0031936) |
| [CG13801](http://flybase.org/cgi-bin/fbidq.html?FBgn0035332) | - | [CG13801](http://flybase.org/cgi-bin/fbidq.html?FBgn0035332) |
| [CG13845](http://flybase.org/cgi-bin/fbidq.html?FBgn0085405) | - | [CG34376](http://flybase.org/cgi-bin/fbidq.html?FBgn0085405) |
| [CG13905](http://flybase.org/cgi-bin/fbidq.html?FBgn0035176) | - | [CG13905](http://flybase.org/cgi-bin/fbidq.html?FBgn0035176) |
| [CG13928](http://flybase.org/cgi-bin/fbidq.html?FBgn0035246) | - | [CG13928](http://flybase.org/cgi-bin/fbidq.html?FBgn0035246) |
| [CG13983](http://flybase.org/cgi-bin/fbidq.html?FBgn0031792) | - | [CG13983](http://flybase.org/cgi-bin/fbidq.html?FBgn0031792) |
| [CG13988](http://flybase.org/cgi-bin/fbidq.html?FBgn0085409) | - | [CG34380](http://flybase.org/cgi-bin/fbidq.html?FBgn0085409) |
| [CG14117](http://flybase.org/cgi-bin/fbidq.html?FBgn0036331) | - | [CG14117](http://flybase.org/cgi-bin/fbidq.html?FBgn0036331) |
| [CG14131](http://flybase.org/cgi-bin/fbidq.html?FBgn0036205) | - | [CG14131](http://flybase.org/cgi-bin/fbidq.html?FBgn0036205) |
| [CG14218](http://flybase.org/cgi-bin/fbidq.html?FBgn0031031) | - | [CG14218](http://flybase.org/cgi-bin/fbidq.html?FBgn0031031) |
| [CG14249](http://flybase.org/cgi-bin/fbidq.html?FBgn0250908) | beat-VII | [beat-VII](http://flybase.org/cgi-bin/fbidq.html?FBgn0250908) |
| [CG14262](http://flybase.org/cgi-bin/fbidq.html?FBgn0039503) | - | [CG14262](http://flybase.org/cgi-bin/fbidq.html?FBgn0039503) |
| [CG14302](http://flybase.org/cgi-bin/fbidq.html?FBgn0038647) | - | [CG14302](http://flybase.org/cgi-bin/fbidq.html?FBgn0038647) |
| [CG14405](http://flybase.org/cgi-bin/fbidq.html?FBgn0032888) | Chemosensory protein B 38c | [CheB38c](http://flybase.org/cgi-bin/fbidq.html?FBgn0032888) |
| [CG14411](http://flybase.org/cgi-bin/fbidq.html?FBgn0030582) | - | [CG14411](http://flybase.org/cgi-bin/fbidq.html?FBgn0030582) |
| [CG14445](http://flybase.org/cgi-bin/fbidq.html?FBgn0029851) | - | [CG14445](http://flybase.org/cgi-bin/fbidq.html?FBgn0029851) |
| [CG14542](http://flybase.org/cgi-bin/fbidq.html?FBgn0039402) | - | [CG14542](http://flybase.org/cgi-bin/fbidq.html?FBgn0039402) |
| [CG14590](http://flybase.org/cgi-bin/fbidq.html?FBgn0033061) | - | [CG14590](http://flybase.org/cgi-bin/fbidq.html?FBgn0033061) |
| [CG14610](http://flybase.org/cgi-bin/fbidq.html?FBgn0037477) | - | [CG14610](http://flybase.org/cgi-bin/fbidq.html?FBgn0037477) |
| [CG14615](http://flybase.org/cgi-bin/fbidq.html?FBgn0031184) | - | [CG14615](http://flybase.org/cgi-bin/fbidq.html?FBgn0031184) |
| [CG14645](http://flybase.org/cgi-bin/fbidq.html?FBgn0040687) | - | [CG14645](http://flybase.org/cgi-bin/fbidq.html?FBgn0040687) |
| [CG14669](http://flybase.org/cgi-bin/fbidq.html?FBgn0037326) | - | [CG14669](http://flybase.org/cgi-bin/fbidq.html?FBgn0037326) |
| [CG14673](http://flybase.org/cgi-bin/fbidq.html?FBgn0037352) | - | [CG14673](http://flybase.org/cgi-bin/fbidq.html?FBgn0037352) |
| [CG14695](http://flybase.org/cgi-bin/fbidq.html?FBgn0037850) | - | [CG14695](http://flybase.org/cgi-bin/fbidq.html?FBgn0037850) |
| [CG14735](http://flybase.org/cgi-bin/fbidq.html?FBgn0037985) | short spindle 5 | [ssp5](http://flybase.org/cgi-bin/fbidq.html?FBgn0037985) |
| [CG14748](http://flybase.org/cgi-bin/fbidq.html?FBgn0259226) | - | [CG42326](http://flybase.org/cgi-bin/fbidq.html?FBgn0259226) |
| [CG14752](http://flybase.org/cgi-bin/fbidq.html?FBgn0033307) | - | [CG14752](http://flybase.org/cgi-bin/fbidq.html?FBgn0033307) |
| [CG14789](http://flybase.org/cgi-bin/fbidq.html?FBgn0027791) | O-fucosyltransferase 2 | [O-fut2](http://flybase.org/cgi-bin/fbidq.html?FBgn0027791) |
| [CG1483](http://flybase.org/cgi-bin/fbidq.html?FBgn0002645) | Microtubule-associated protein 205 | [Map205](http://flybase.org/cgi-bin/fbidq.html?FBgn0002645) |
| [CG1484](http://flybase.org/cgi-bin/fbidq.html?FBgn0000709) | flightless I | [fliI](http://flybase.org/cgi-bin/fbidq.html?FBgn0000709) |
| [CG14850](http://flybase.org/cgi-bin/fbidq.html?FBgn0038239) | - | [CG14850](http://flybase.org/cgi-bin/fbidq.html?FBgn0038239) |
| [CG1487](http://flybase.org/cgi-bin/fbidq.html?FBgn0040206) | kurtz | [krz](http://flybase.org/cgi-bin/fbidq.html?FBgn0040206) |
| [CG14902](http://flybase.org/cgi-bin/fbidq.html?FBgn0028381) | death executioner caspase related to Apopain/Yama | [decay](http://flybase.org/cgi-bin/fbidq.html?FBgn0028381) |
| [CG14904](http://flybase.org/cgi-bin/fbidq.html?FBgn0020907) | Sarcoplasmic calcium-binding protein 2 | [Scp2](http://flybase.org/cgi-bin/fbidq.html?FBgn0020907) |
| [CG14962](http://flybase.org/cgi-bin/fbidq.html?FBgn0035407) | - | [CG14962](http://flybase.org/cgi-bin/fbidq.html?FBgn0035407) |
| [CG14969](http://flybase.org/cgi-bin/fbidq.html?FBgn0035440) | - | [CG14969](http://flybase.org/cgi-bin/fbidq.html?FBgn0035440) |
| [CG14973](http://flybase.org/cgi-bin/fbidq.html?FBgn0259224) | - | [CG42324](http://flybase.org/cgi-bin/fbidq.html?FBgn0259224) |
| [CG14984](http://flybase.org/cgi-bin/fbidq.html?FBgn0035480) | - | [CG14984](http://flybase.org/cgi-bin/fbidq.html?FBgn0035480) |
| [CG15060](http://flybase.org/cgi-bin/fbidq.html?FBgn0030903) | - | [CG15060](http://flybase.org/cgi-bin/fbidq.html?FBgn0030903) |
| [CG15068](http://flybase.org/cgi-bin/fbidq.html?FBgn0040733) | - | [CG15068](http://flybase.org/cgi-bin/fbidq.html?FBgn0040733) |
| [CG15088](http://flybase.org/cgi-bin/fbidq.html?FBgn0034381) | - | [CG15088](http://flybase.org/cgi-bin/fbidq.html?FBgn0034381) |
| [CG15094](http://flybase.org/cgi-bin/fbidq.html?FBgn0034392) | - | [CG15094](http://flybase.org/cgi-bin/fbidq.html?FBgn0034392) |
| [CG15199](http://flybase.org/cgi-bin/fbidq.html?FBgn0030270) | - | [CG15199](http://flybase.org/cgi-bin/fbidq.html?FBgn0030270) |
| [CG15212](http://flybase.org/cgi-bin/fbidq.html?FBgn0040842) | - | [CG15212](http://flybase.org/cgi-bin/fbidq.html?FBgn0040842) |
| [CG15227](http://flybase.org/cgi-bin/fbidq.html?FBgn0034554) | - | [CG15227](http://flybase.org/cgi-bin/fbidq.html?FBgn0034554) |
| [CG15236](http://flybase.org/cgi-bin/fbidq.html?FBgn0033108) | - | [CG15236](http://flybase.org/cgi-bin/fbidq.html?FBgn0033108) |
| [CG15282](http://flybase.org/cgi-bin/fbidq.html?FBgn0028855) | - | [CG15282](http://flybase.org/cgi-bin/fbidq.html?FBgn0028855) |
| [CG15336](http://flybase.org/cgi-bin/fbidq.html?FBgn0030009) | - | [CG15336](http://flybase.org/cgi-bin/fbidq.html?FBgn0030009) |
| [CG15353](http://flybase.org/cgi-bin/fbidq.html?FBgn0040718) | - | [CG15353](http://flybase.org/cgi-bin/fbidq.html?FBgn0040718) |
| [CG15358](http://flybase.org/cgi-bin/fbidq.html?FBgn0031373) | - | [CG15358](http://flybase.org/cgi-bin/fbidq.html?FBgn0031373) |
| [CG15418](http://flybase.org/cgi-bin/fbidq.html?FBgn0031554) | - | [CG15418](http://flybase.org/cgi-bin/fbidq.html?FBgn0031554) |
| [CG15429](http://flybase.org/cgi-bin/fbidq.html?FBgn0031596) | - | [CG15429](http://flybase.org/cgi-bin/fbidq.html?FBgn0031596) |
| [CG15499](http://flybase.org/cgi-bin/fbidq.html?FBgn0038891) | - | [CG15499](http://flybase.org/cgi-bin/fbidq.html?FBgn0038891) |
| [CG15515](http://flybase.org/cgi-bin/fbidq.html?FBgn0039719) | - | [CG15515](http://flybase.org/cgi-bin/fbidq.html?FBgn0039719) |
| [CG15520](http://flybase.org/cgi-bin/fbidq.html?FBgn0039722) | capability | [capa](http://flybase.org/cgi-bin/fbidq.html?FBgn0039722) |
| [CG15531](http://flybase.org/cgi-bin/fbidq.html?FBgn0039755) | - | [CG15531](http://flybase.org/cgi-bin/fbidq.html?FBgn0039755) |
| [CG15577](http://flybase.org/cgi-bin/fbidq.html?FBgn0040904) | - | [CG15577](http://flybase.org/cgi-bin/fbidq.html?FBgn0040904) |
| [CG15578](http://flybase.org/cgi-bin/fbidq.html?FBgn0040905) | - | [CG15578](http://flybase.org/cgi-bin/fbidq.html?FBgn0040905) |
| [CG15829](http://flybase.org/cgi-bin/fbidq.html?FBgn0035743) | - | [CG15829](http://flybase.org/cgi-bin/fbidq.html?FBgn0035743) |
| [CG15880](http://flybase.org/cgi-bin/fbidq.html?FBgn0031283) | - | [CG15880](http://flybase.org/cgi-bin/fbidq.html?FBgn0031283) |
| [CG1628](http://flybase.org/cgi-bin/fbidq.html?FBgn0030218) | - | [CG1628](http://flybase.org/cgi-bin/fbidq.html?FBgn0030218) |
| [CG1657](http://flybase.org/cgi-bin/fbidq.html?FBgn0030286) | - | [CG1657](http://flybase.org/cgi-bin/fbidq.html?FBgn0030286) |
| [CG16707](http://flybase.org/cgi-bin/fbidq.html?FBgn0045823) | visgun | [vsg](http://flybase.org/cgi-bin/fbidq.html?FBgn0045823) |
| [CG16743](http://flybase.org/cgi-bin/fbidq.html?FBgn0032322) | - | [CG16743](http://flybase.org/cgi-bin/fbidq.html?FBgn0032322) |
| [CG16747](http://flybase.org/cgi-bin/fbidq.html?FBgn0014184) | Ornithine decarboxylase antizyme | [Oda](http://flybase.org/cgi-bin/fbidq.html?FBgn0014184) |
| [CG16756](http://flybase.org/cgi-bin/fbidq.html?FBgn0029765) | - | [CG16756](http://flybase.org/cgi-bin/fbidq.html?FBgn0029765) |
| [CG16761](http://flybase.org/cgi-bin/fbidq.html?FBgn0035344) | Cyp4d20 | [Cyp4d20](http://flybase.org/cgi-bin/fbidq.html?FBgn0035344) |
| [CG16790](http://flybase.org/cgi-bin/fbidq.html?FBgn0037713) | - | [CG16790](http://flybase.org/cgi-bin/fbidq.html?FBgn0037713) |
| [CG16801](http://flybase.org/cgi-bin/fbidq.html?FBgn0034012) | Hormone receptor 51 | [Hr51](http://flybase.org/cgi-bin/fbidq.html?FBgn0034012) |
| [CG16820](http://flybase.org/cgi-bin/fbidq.html?FBgn0032495) | - | [CG16820](http://flybase.org/cgi-bin/fbidq.html?FBgn0032495) |
| [CG16825](http://flybase.org/cgi-bin/fbidq.html?FBgn0032503) | - | [CG16825](http://flybase.org/cgi-bin/fbidq.html?FBgn0032503) |
| [CG16838](http://flybase.org/cgi-bin/fbidq.html?FBgn0036574) | - | [CG16838](http://flybase.org/cgi-bin/fbidq.html?FBgn0036574) |
| [CG16879](http://flybase.org/cgi-bin/fbidq.html?FBgn0028904) | - | [CG16879](http://flybase.org/cgi-bin/fbidq.html?FBgn0028904) |
| [CG16944](http://flybase.org/cgi-bin/fbidq.html?FBgn0003360) | stress-sensitive B | [sesB](http://flybase.org/cgi-bin/fbidq.html?FBgn0003360) |
| [CG16996](http://flybase.org/cgi-bin/fbidq.html?FBgn0032412) | - | [CG16996](http://flybase.org/cgi-bin/fbidq.html?FBgn0032412) |
| [CG17010](http://flybase.org/cgi-bin/fbidq.html?FBgn0032424) | - | [CG17010](http://flybase.org/cgi-bin/fbidq.html?FBgn0032424) |
| [CG17063](http://flybase.org/cgi-bin/fbidq.html?FBgn0027107) | inx6 | [inx6](http://flybase.org/cgi-bin/fbidq.html?FBgn0027107) |
| [CG17173](http://flybase.org/cgi-bin/fbidq.html?FBgn0036447) | - | [CG17173](http://flybase.org/cgi-bin/fbidq.html?FBgn0036447) |
| [CG17191](http://flybase.org/cgi-bin/fbidq.html?FBgn0039473) | - | [CG17191](http://flybase.org/cgi-bin/fbidq.html?FBgn0039473) |
| [CG17200](http://flybase.org/cgi-bin/fbidq.html?FBgn0040253) | Ugt86Dg | [Ugt86Dg](http://flybase.org/cgi-bin/fbidq.html?FBgn0040253) |
| [CG17280](http://flybase.org/cgi-bin/fbidq.html?FBgn0034877) | levy | [levy](http://flybase.org/cgi-bin/fbidq.html?FBgn0034877) |
| [CG17369](http://flybase.org/cgi-bin/fbidq.html?FBgn0005671) | Vacuolar H[+]-ATPase 55kD B subunit | [Vha55](http://flybase.org/cgi-bin/fbidq.html?FBgn0005671) |
| [CG17475](http://flybase.org/cgi-bin/fbidq.html?FBgn0038481) | - | [CG17475](http://flybase.org/cgi-bin/fbidq.html?FBgn0038481) |
| [CG17508](http://flybase.org/cgi-bin/fbidq.html?FBgn0039970) | - | [CG17508](http://flybase.org/cgi-bin/fbidq.html?FBgn0039970) |
| [CG17524](http://flybase.org/cgi-bin/fbidq.html?FBgn0063497) | Glutathione S transferase E3 | [GstE3](http://flybase.org/cgi-bin/fbidq.html?FBgn0063497) |
| [CG17567](http://flybase.org/cgi-bin/fbidq.html?FBgn0040994) | - | [CG17567](http://flybase.org/cgi-bin/fbidq.html?FBgn0040994) |
| [CG17601](http://flybase.org/cgi-bin/fbidq.html?FBgn0031197) | - | [CG17601](http://flybase.org/cgi-bin/fbidq.html?FBgn0031197) |
| [CG1771](http://flybase.org/cgi-bin/fbidq.html?FBgn0004456) | multiple edematous wings | [mew](http://flybase.org/cgi-bin/fbidq.html?FBgn0004456) |
| [CG1773](http://flybase.org/cgi-bin/fbidq.html?FBgn0033439) | - | [CG1773](http://flybase.org/cgi-bin/fbidq.html?FBgn0033439) |
| [CG17735](http://flybase.org/cgi-bin/fbidq.html?FBgn0260794) | - | [CG42574](http://flybase.org/cgi-bin/fbidq.html?FBgn0260794) |
| [CG1774](http://flybase.org/cgi-bin/fbidq.html?FBgn0039856) | - | [CG1774](http://flybase.org/cgi-bin/fbidq.html?FBgn0039856) |
| [CG17919](http://flybase.org/cgi-bin/fbidq.html?FBgn0037433) | - | [CG17919](http://flybase.org/cgi-bin/fbidq.html?FBgn0037433) |
| [CG17927](http://flybase.org/cgi-bin/fbidq.html?FBgn0086783) | Myosin heavy chain | [Mhc](http://flybase.org/cgi-bin/fbidq.html?FBgn0086783) |
| [CG1795](http://flybase.org/cgi-bin/fbidq.html?FBgn0027864) | Ogg1 | [Ogg1](http://flybase.org/cgi-bin/fbidq.html?FBgn0027864) |
| [CG1796](http://flybase.org/cgi-bin/fbidq.html?FBgn0030365) | Transport and Golgi organization 4 | [Tango4](http://flybase.org/cgi-bin/fbidq.html?FBgn0030365) |
| [CG17976](http://flybase.org/cgi-bin/fbidq.html?FBgn0028561) | sugar transporter 3 | [sut3](http://flybase.org/cgi-bin/fbidq.html?FBgn0028561) |
| [CG18011](http://flybase.org/cgi-bin/fbidq.html?FBgn0033491) | - | [CG18011](http://flybase.org/cgi-bin/fbidq.html?FBgn0033491) |
| [CG18066](http://flybase.org/cgi-bin/fbidq.html?FBgn0034517) | Cuticular protein 57A | [Cpr57A](http://flybase.org/cgi-bin/fbidq.html?FBgn0034517) |
| [CG18102](http://flybase.org/cgi-bin/fbidq.html?FBgn0003392) | shibire | [shi](http://flybase.org/cgi-bin/fbidq.html?FBgn0003392) |
| [CG18156](http://flybase.org/cgi-bin/fbidq.html?FBgn0035725) | Mis12 | [Mis12](http://flybase.org/cgi-bin/fbidq.html?FBgn0035725) |
| [CG18178](http://flybase.org/cgi-bin/fbidq.html?FBgn0036035) | - | [CG18178](http://flybase.org/cgi-bin/fbidq.html?FBgn0036035) |
| [CG18231](http://flybase.org/cgi-bin/fbidq.html?FBgn0036796) | - | [CG18231](http://flybase.org/cgi-bin/fbidq.html?FBgn0036796) |
| [CG18250](http://flybase.org/cgi-bin/fbidq.html?FBgn0034072) | Dystroglycan | [Dg](http://flybase.org/cgi-bin/fbidq.html?FBgn0034072) |
| [CG18278](http://flybase.org/cgi-bin/fbidq.html?FBgn0033836) | - | [CG18278](http://flybase.org/cgi-bin/fbidq.html?FBgn0033836) |
| [CG18285](http://flybase.org/cgi-bin/fbidq.html?FBgn0013467) | igloo | [igl](http://flybase.org/cgi-bin/fbidq.html?FBgn0013467) |
| [CG18335](http://flybase.org/cgi-bin/fbidq.html?FBgn0033610) | - | [CG18335](http://flybase.org/cgi-bin/fbidq.html?FBgn0033610) |
| [CG18347](http://flybase.org/cgi-bin/fbidq.html?FBgn0260743) | - | [CG18347](http://flybase.org/cgi-bin/fbidq.html?FBgn0260743) |
| [CG18349](http://flybase.org/cgi-bin/fbidq.html?FBgn0036109) | Cuticular protein 67Fa2 | [Cpr67Fa2](http://flybase.org/cgi-bin/fbidq.html?FBgn0036109) |
| [CG18369](http://flybase.org/cgi-bin/fbidq.html?FBgn0033860) | - | [CG18369](http://flybase.org/cgi-bin/fbidq.html?FBgn0033860) |
| [CG1837](http://flybase.org/cgi-bin/fbidq.html?FBgn0030329) | - | [CG1837](http://flybase.org/cgi-bin/fbidq.html?FBgn0030329) |
| [CG18437](http://flybase.org/cgi-bin/fbidq.html?FBgn0039536) | - | [CG18437](http://flybase.org/cgi-bin/fbidq.html?FBgn0039536) |
| [CG18445](http://flybase.org/cgi-bin/fbidq.html?FBgn0033476) | - | [CG18445](http://flybase.org/cgi-bin/fbidq.html?FBgn0033476) |
| [CG18545](http://flybase.org/cgi-bin/fbidq.html?FBgn0037812) | - | [CG18545](http://flybase.org/cgi-bin/fbidq.html?FBgn0037812) |
| [CG18558](http://flybase.org/cgi-bin/fbidq.html?FBgn0031469) | - | [CG18558](http://flybase.org/cgi-bin/fbidq.html?FBgn0031469) |
| [CG18585](http://flybase.org/cgi-bin/fbidq.html?FBgn0031929) | - | [CG18585](http://flybase.org/cgi-bin/fbidq.html?FBgn0031929) |
| [CG18594](http://flybase.org/cgi-bin/fbidq.html?FBgn0038973) | - | [CG18594](http://flybase.org/cgi-bin/fbidq.html?FBgn0038973) |
| [CG1865](http://flybase.org/cgi-bin/fbidq.html?FBgn0024293) | Serine protease inhibitor 43Ab | [Spn43Ab](http://flybase.org/cgi-bin/fbidq.html?FBgn0024293) |
| [CG18659](http://flybase.org/cgi-bin/fbidq.html?FBgn0027561) | - | [CG18659](http://flybase.org/cgi-bin/fbidq.html?FBgn0027561) |
| [CG18773](http://flybase.org/cgi-bin/fbidq.html?FBgn0020643) | Lcp65Ab2 | [Lcp65Ab2](http://flybase.org/cgi-bin/fbidq.html?FBgn0020643) |
| [CG18779](http://flybase.org/cgi-bin/fbidq.html?FBgn0086611) | Larval cuticle protein | [Lcp65Ag3](http://flybase.org/cgi-bin/fbidq.html?FBgn0086611) |
| [CG18783](http://flybase.org/cgi-bin/fbidq.html?FBgn0028420) | Kruppel homolog 1 | [Kr-h1](http://flybase.org/cgi-bin/fbidq.html?FBgn0028420) |
| [CG1922](http://flybase.org/cgi-bin/fbidq.html?FBgn0028996) | onecut | [onecut](http://flybase.org/cgi-bin/fbidq.html?FBgn0028996) |
| [CG1938](http://flybase.org/cgi-bin/fbidq.html?FBgn0030276) | Dynein light intermediate chain | [Dlic](http://flybase.org/cgi-bin/fbidq.html?FBgn0030276) |
| [CG1987](http://flybase.org/cgi-bin/fbidq.html?FBgn0030479) | Rbp1-like | [Rbp1-like](http://flybase.org/cgi-bin/fbidq.html?FBgn0030479) |
| [CG2041](http://flybase.org/cgi-bin/fbidq.html?FBgn0039907) | legless | [lgs](http://flybase.org/cgi-bin/fbidq.html?FBgn0039907) |
| [CG2069](http://flybase.org/cgi-bin/fbidq.html?FBgn0035264) | Oseg4 | [Oseg4](http://flybase.org/cgi-bin/fbidq.html?FBgn0035264) |
| [CG2118](http://flybase.org/cgi-bin/fbidq.html?FBgn0039877) | - | [CG2118](http://flybase.org/cgi-bin/fbidq.html?FBgn0039877) |
| [CG2187](http://flybase.org/cgi-bin/fbidq.html?FBgn0017448) | - | [CG2187](http://flybase.org/cgi-bin/fbidq.html?FBgn0017448) |
| [CG2213](http://flybase.org/cgi-bin/fbidq.html?FBgn0035210) | mitotic spindle density 5 | [msd5](http://flybase.org/cgi-bin/fbidq.html?FBgn0035210) |
| [CG2229](http://flybase.org/cgi-bin/fbidq.html?FBgn0039777) | Jonah 99Fii | [Jon99Fii](http://flybase.org/cgi-bin/fbidq.html?FBgn0039777) |
| [CG2345](http://flybase.org/cgi-bin/fbidq.html?FBgn0000552) | Ecdysone-dependent gene 84A | [Edg84A](http://flybase.org/cgi-bin/fbidq.html?FBgn0000552) |
| [CG2448](http://flybase.org/cgi-bin/fbidq.html?FBgn0030327) | alpha1,6-fucosyltransferase | [FucT6](http://flybase.org/cgi-bin/fbidq.html?FBgn0030327) |
| [CG2522](http://flybase.org/cgi-bin/fbidq.html?FBgn0010391) | GTP-binding protein | [Gtp-bp](http://flybase.org/cgi-bin/fbidq.html?FBgn0010391) |
| [CG2543](http://flybase.org/cgi-bin/fbidq.html?FBgn0030407) | - | [CG2543](http://flybase.org/cgi-bin/fbidq.html?FBgn0030407) |
| [CG2560](http://flybase.org/cgi-bin/fbidq.html?FBgn0030394) | Cuticular protein 11A | [Cpr11A](http://flybase.org/cgi-bin/fbidq.html?FBgn0030394) |
| [CG2841](http://flybase.org/cgi-bin/fbidq.html?FBgn0003159) | proximal to raf | [ptr](http://flybase.org/cgi-bin/fbidq.html?FBgn0003159) |
| [CG2893](http://flybase.org/cgi-bin/fbidq.html?FBgn0040030) | - | [CG2893](http://flybase.org/cgi-bin/fbidq.html?FBgn0040030) |
| [CG30037](http://flybase.org/cgi-bin/fbidq.html?FBgn0050037) | - | [CG30037](http://flybase.org/cgi-bin/fbidq.html?FBgn0050037) |
| [CG30047](http://flybase.org/cgi-bin/fbidq.html?FBgn0050047) | - | [CG30047](http://flybase.org/cgi-bin/fbidq.html?FBgn0050047) |
| [CG30116](http://flybase.org/cgi-bin/fbidq.html?FBgn0028496) | - | [CG30116](http://flybase.org/cgi-bin/fbidq.html?FBgn0028496) |
| [CG30118](http://flybase.org/cgi-bin/fbidq.html?FBgn0050118) | - | [CG30118](http://flybase.org/cgi-bin/fbidq.html?FBgn0050118) |
| [CG30164](http://flybase.org/cgi-bin/fbidq.html?FBgn0259210) | prominin | [prom](http://flybase.org/cgi-bin/fbidq.html?FBgn0259210) |
| [CG30175](http://flybase.org/cgi-bin/fbidq.html?FBgn0053519) | Unc-89 | [Unc-89](http://flybase.org/cgi-bin/fbidq.html?FBgn0053519) |
| [CG30188](http://flybase.org/cgi-bin/fbidq.html?FBgn0085400) | - | [CG34371](http://flybase.org/cgi-bin/fbidq.html?FBgn0085400) |
| [CG30219](http://flybase.org/cgi-bin/fbidq.html?FBgn0085475) | - | [CG34446](http://flybase.org/cgi-bin/fbidq.html?FBgn0085475) |
| [CG30272](http://flybase.org/cgi-bin/fbidq.html?FBgn0050272) | - | [CG30272](http://flybase.org/cgi-bin/fbidq.html?FBgn0050272) |
| [CG30332](http://flybase.org/cgi-bin/fbidq.html?FBgn0050332) | - | [CG30332](http://flybase.org/cgi-bin/fbidq.html?FBgn0050332) |
| [CG30339](http://flybase.org/cgi-bin/fbidq.html?FBgn0050339) | - | [CG30339](http://flybase.org/cgi-bin/fbidq.html?FBgn0050339) |
| [CG30426](http://flybase.org/cgi-bin/fbidq.html?FBgn0086908) | eggless | [egg](http://flybase.org/cgi-bin/fbidq.html?FBgn0086908) |
| [CG30438](http://flybase.org/cgi-bin/fbidq.html?FBgn0050438) | - | [CG30438](http://flybase.org/cgi-bin/fbidq.html?FBgn0050438) |
| [CG30457](http://flybase.org/cgi-bin/fbidq.html?FBgn0050457) | - | [CG30457](http://flybase.org/cgi-bin/fbidq.html?FBgn0050457) |
| [CG3047](http://flybase.org/cgi-bin/fbidq.html?FBgn0003372) | Salivary gland secretion 1 | [Sgs1](http://flybase.org/cgi-bin/fbidq.html?FBgn0003372) |
| [CG30470](http://flybase.org/cgi-bin/fbidq.html?FBgn0050470) | - | [CG30470](http://flybase.org/cgi-bin/fbidq.html?FBgn0050470) |
| [CG30501](http://flybase.org/cgi-bin/fbidq.html?FBgn0050501) | - | [CG30501](http://flybase.org/cgi-bin/fbidq.html?FBgn0050501) |
| [CG31002](http://flybase.org/cgi-bin/fbidq.html?FBgn0051002) | - | [CG31002](http://flybase.org/cgi-bin/fbidq.html?FBgn0051002) |
| [CG31018](http://flybase.org/cgi-bin/fbidq.html?FBgn0054041) | - | [CG34041](http://flybase.org/cgi-bin/fbidq.html?FBgn0054041) |
| [CG31105](http://flybase.org/cgi-bin/fbidq.html?FBgn0051105) | - | [CG31105](http://flybase.org/cgi-bin/fbidq.html?FBgn0051105) |
| [CG31182](http://flybase.org/cgi-bin/fbidq.html?FBgn0051182) | - | [CG31182](http://flybase.org/cgi-bin/fbidq.html?FBgn0051182) |
| [CG31227](http://flybase.org/cgi-bin/fbidq.html?FBgn0051227) | - | [CG31227](http://flybase.org/cgi-bin/fbidq.html?FBgn0051227) |
| [CG31264](http://flybase.org/cgi-bin/fbidq.html?FBgn0250829) | Poly-glutamine tract binding protein 1 | [PQBP-1](http://flybase.org/cgi-bin/fbidq.html?FBgn0250829) |
| [CG31293](http://flybase.org/cgi-bin/fbidq.html?FBgn0003227) | recombination-defective | [rec](http://flybase.org/cgi-bin/fbidq.html?FBgn0003227) |
| [CG31294](http://flybase.org/cgi-bin/fbidq.html?FBgn0051294) | - | [CG31294](http://flybase.org/cgi-bin/fbidq.html?FBgn0051294) |
| [CG31335](http://flybase.org/cgi-bin/fbidq.html?FBgn0045468) | Gustatory receptor 93d | [Gr93d](http://flybase.org/cgi-bin/fbidq.html?FBgn0045468) |
| [CG31351](http://flybase.org/cgi-bin/fbidq.html?FBgn0250910) | Octbeta3R | [Octbeta3R](http://flybase.org/cgi-bin/fbidq.html?FBgn0250910) |
| [CG31352](http://flybase.org/cgi-bin/fbidq.html?FBgn0051352) | - | [CG31352](http://flybase.org/cgi-bin/fbidq.html?FBgn0051352) |
| [CG3139](http://flybase.org/cgi-bin/fbidq.html?FBgn0004242) | Synaptotagmin 1 | [Syt1](http://flybase.org/cgi-bin/fbidq.html?FBgn0004242) |
| [CG31397](http://flybase.org/cgi-bin/fbidq.html?FBgn0051397) | - | [CG31397](http://flybase.org/cgi-bin/fbidq.html?FBgn0051397) |
| [CG31446](http://flybase.org/cgi-bin/fbidq.html?FBgn0051446) | - | [CG31446](http://flybase.org/cgi-bin/fbidq.html?FBgn0051446) |
| [CG31495](http://flybase.org/cgi-bin/fbidq.html?FBgn0051495) | - | [CG31495](http://flybase.org/cgi-bin/fbidq.html?FBgn0051495) |
| [CG31507](http://flybase.org/cgi-bin/fbidq.html?FBgn0044809) | Turandot Z | [TotZ](http://flybase.org/cgi-bin/fbidq.html?FBgn0044809) |
| [CG31522](http://flybase.org/cgi-bin/fbidq.html?FBgn0051522) | - | [CG31522](http://flybase.org/cgi-bin/fbidq.html?FBgn0051522) |
| [CG31530](http://flybase.org/cgi-bin/fbidq.html?FBgn0051530) | - | [CG31530](http://flybase.org/cgi-bin/fbidq.html?FBgn0051530) |
| [CG31558](http://flybase.org/cgi-bin/fbidq.html?FBgn0046875) | Odorant-binding protein 83g | [Obp83g](http://flybase.org/cgi-bin/fbidq.html?FBgn0046875) |
| [CG31606](http://flybase.org/cgi-bin/fbidq.html?FBgn0051606) | - | [CG31606](http://flybase.org/cgi-bin/fbidq.html?FBgn0051606) |
| [CG31609](http://flybase.org/cgi-bin/fbidq.html?FBgn0051609) | - | [CG31609](http://flybase.org/cgi-bin/fbidq.html?FBgn0051609) |
| [CG31628](http://flybase.org/cgi-bin/fbidq.html?FBgn0000053) | adenosine 3 | [ade3](http://flybase.org/cgi-bin/fbidq.html?FBgn0000053) |
| [CG31637](http://flybase.org/cgi-bin/fbidq.html?FBgn0051637) | - | [CG31637](http://flybase.org/cgi-bin/fbidq.html?FBgn0051637) |
| [CG31690](http://flybase.org/cgi-bin/fbidq.html?FBgn0051690) | - | [CG31690](http://flybase.org/cgi-bin/fbidq.html?FBgn0051690) |
| [CG31732](http://flybase.org/cgi-bin/fbidq.html?FBgn0045842) | yuri gagarin | [yuri](http://flybase.org/cgi-bin/fbidq.html?FBgn0045842) |
| [CG31735](http://flybase.org/cgi-bin/fbidq.html?FBgn0051735) | - | [CG31735](http://flybase.org/cgi-bin/fbidq.html?FBgn0051735) |
| [CG31736](http://flybase.org/cgi-bin/fbidq.html?FBgn0051736) | - | [CG31736](http://flybase.org/cgi-bin/fbidq.html?FBgn0051736) |
| [CG31819](http://flybase.org/cgi-bin/fbidq.html?FBgn0028893) | - | [CG31819](http://flybase.org/cgi-bin/fbidq.html?FBgn0028893) |
| [CG31824](http://flybase.org/cgi-bin/fbidq.html?FBgn0051824) | - | [CG31824](http://flybase.org/cgi-bin/fbidq.html?FBgn0051824) |
| [CG31834](http://flybase.org/cgi-bin/fbidq.html?FBgn0028927) | - | [CG31834](http://flybase.org/cgi-bin/fbidq.html?FBgn0028927) |
| [CG31878](http://flybase.org/cgi-bin/fbidq.html?FBgn0259713) | - | [CG42367](http://flybase.org/cgi-bin/fbidq.html?FBgn0259713) |
| [CG31898](http://flybase.org/cgi-bin/fbidq.html?FBgn0051898) | - | [CG31898](http://flybase.org/cgi-bin/fbidq.html?FBgn0051898) |
| [CG31928](http://flybase.org/cgi-bin/fbidq.html?FBgn0051928) | - | [CG31928](http://flybase.org/cgi-bin/fbidq.html?FBgn0051928) |
| [CG3194](http://flybase.org/cgi-bin/fbidq.html?FBgn0033087) | - | [CG3194](http://flybase.org/cgi-bin/fbidq.html?FBgn0033087) |
| [CG31961](http://flybase.org/cgi-bin/fbidq.html?FBgn0051961) | - | [CG31961](http://flybase.org/cgi-bin/fbidq.html?FBgn0051961) |
| [CG31988](http://flybase.org/cgi-bin/fbidq.html?FBgn0051988) | - | [CG31988](http://flybase.org/cgi-bin/fbidq.html?FBgn0051988) |
| [CG32037](http://flybase.org/cgi-bin/fbidq.html?FBgn0052037) | - | [CG32037](http://flybase.org/cgi-bin/fbidq.html?FBgn0052037) |
| [CG32177](http://flybase.org/cgi-bin/fbidq.html?FBgn0052177) | - | [CG32177](http://flybase.org/cgi-bin/fbidq.html?FBgn0052177) |
| [CG32219](http://flybase.org/cgi-bin/fbidq.html?FBgn0052219) | - | [CG32219](http://flybase.org/cgi-bin/fbidq.html?FBgn0052219) |
| [CG32295](http://flybase.org/cgi-bin/fbidq.html?FBgn0260480) | - | [CG32295](http://flybase.org/cgi-bin/fbidq.html?FBgn0260480) |
| [CG32336](http://flybase.org/cgi-bin/fbidq.html?FBgn0052336) | - | [CG32336](http://flybase.org/cgi-bin/fbidq.html?FBgn0052336) |
| [CG32390](http://flybase.org/cgi-bin/fbidq.html?FBgn0259173) | cornetto | [corn](http://flybase.org/cgi-bin/fbidq.html?FBgn0259173) |
| [CG32391](http://flybase.org/cgi-bin/fbidq.html?FBgn0052391) | - | [CG32391](http://flybase.org/cgi-bin/fbidq.html?FBgn0052391) |
| [CG32401](http://flybase.org/cgi-bin/fbidq.html?FBgn0041625) | Odorant receptor 65a | [Or65a](http://flybase.org/cgi-bin/fbidq.html?FBgn0041625) |
| [CG32434](http://flybase.org/cgi-bin/fbidq.html?FBgn0026179) | schizo | [siz](http://flybase.org/cgi-bin/fbidq.html?FBgn0026179) |
| [CG32462](http://flybase.org/cgi-bin/fbidq.html?FBgn0052462) | - | [CG32462](http://flybase.org/cgi-bin/fbidq.html?FBgn0052462) |
| [CG32513](http://flybase.org/cgi-bin/fbidq.html?FBgn0031150) | bves | [bves](http://flybase.org/cgi-bin/fbidq.html?FBgn0031150) |
| [CG32590](http://flybase.org/cgi-bin/fbidq.html?FBgn0052590) | - | [CG32590](http://flybase.org/cgi-bin/fbidq.html?FBgn0052590) |
| [CG32675](http://flybase.org/cgi-bin/fbidq.html?FBgn0052675) | Transport and Golgi organization 5 | [Tango5](http://flybase.org/cgi-bin/fbidq.html?FBgn0052675) |
| [CG32683](http://flybase.org/cgi-bin/fbidq.html?FBgn0052683) | - | [CG32683](http://flybase.org/cgi-bin/fbidq.html?FBgn0052683) |
| [CG32717](http://flybase.org/cgi-bin/fbidq.html?FBgn0243505) | stardust | [sdt](http://flybase.org/cgi-bin/fbidq.html?FBgn0243505) |
| [CG32718](http://flybase.org/cgi-bin/fbidq.html?FBgn0052718) | - | [CG32718](http://flybase.org/cgi-bin/fbidq.html?FBgn0052718) |
| [CG32721](http://flybase.org/cgi-bin/fbidq.html?FBgn0027553) | NELF-B | [NELF-B](http://flybase.org/cgi-bin/fbidq.html?FBgn0027553) |
| [CG32762](http://flybase.org/cgi-bin/fbidq.html?FBgn0052762) | - | [CG32762](http://flybase.org/cgi-bin/fbidq.html?FBgn0052762) |
| [CG32770](http://flybase.org/cgi-bin/fbidq.html?FBgn0260971) | - | [CG42594](http://flybase.org/cgi-bin/fbidq.html?FBgn0260971) |
| [CG32800](http://flybase.org/cgi-bin/fbidq.html?FBgn0053548) | msta | [msta](http://flybase.org/cgi-bin/fbidq.html?FBgn0053548) |
| [CG32810](http://flybase.org/cgi-bin/fbidq.html?FBgn0025394) | - | [CG32810](http://flybase.org/cgi-bin/fbidq.html?FBgn0025394) |
| [CG3285](http://flybase.org/cgi-bin/fbidq.html?FBgn0031522) | - | [CG3285](http://flybase.org/cgi-bin/fbidq.html?FBgn0031522) |
| [CG32850](http://flybase.org/cgi-bin/fbidq.html?FBgn0052850) | - | [CG32850](http://flybase.org/cgi-bin/fbidq.html?FBgn0052850) |
| [CG32973](http://flybase.org/cgi-bin/fbidq.html?FBgn0052973) | - | [CG32973](http://flybase.org/cgi-bin/fbidq.html?FBgn0052973) |
| [CG32987](http://flybase.org/cgi-bin/fbidq.html?FBgn0052987) | - | [CG32987](http://flybase.org/cgi-bin/fbidq.html?FBgn0052987) |
| [CG32988](http://flybase.org/cgi-bin/fbidq.html?FBgn0052988) | - | [CG32988](http://flybase.org/cgi-bin/fbidq.html?FBgn0052988) |
| [CG3299](http://flybase.org/cgi-bin/fbidq.html?FBgn0004397) | Vinculin | [Vinc](http://flybase.org/cgi-bin/fbidq.html?FBgn0004397) |
| [CG33083](http://flybase.org/cgi-bin/fbidq.html?FBgn0041224) | Gustatory receptor 97a | [Gr97a](http://flybase.org/cgi-bin/fbidq.html?FBgn0041224) |
| [CG33092](http://flybase.org/cgi-bin/fbidq.html?FBgn0053092) | - | [CG33092](http://flybase.org/cgi-bin/fbidq.html?FBgn0053092) |
| [CG33101](http://flybase.org/cgi-bin/fbidq.html?FBgn0013998) | NEM-sensitive fusion protein 2 | [Nsf2](http://flybase.org/cgi-bin/fbidq.html?FBgn0013998) |
| [CG33115](http://flybase.org/cgi-bin/fbidq.html?FBgn0028542) | nimrod B4 | [nimB4](http://flybase.org/cgi-bin/fbidq.html?FBgn0028542) |
| [CG33147](http://flybase.org/cgi-bin/fbidq.html?FBgn0053147) | Heparan sulfate 3-O sulfotransferase-A | [Hs3st-A](http://flybase.org/cgi-bin/fbidq.html?FBgn0053147) |
| [CG33192](http://flybase.org/cgi-bin/fbidq.html?FBgn0053192) | Metallothionein D | [MtnD](http://flybase.org/cgi-bin/fbidq.html?FBgn0053192) |
| [CG33196](http://flybase.org/cgi-bin/fbidq.html?FBgn0053196) | dumpy | [dp](http://flybase.org/cgi-bin/fbidq.html?FBgn0053196) |
| [CG33209](http://flybase.org/cgi-bin/fbidq.html?FBgn0259236) | comm3 | [comm3](http://flybase.org/cgi-bin/fbidq.html?FBgn0259236) |
| [CG3328](http://flybase.org/cgi-bin/fbidq.html?FBgn0034985) | - | [CG3328](http://flybase.org/cgi-bin/fbidq.html?FBgn0034985) |
| [CG33288](http://flybase.org/cgi-bin/fbidq.html?FBgn0053288) | - | [CG33288](http://flybase.org/cgi-bin/fbidq.html?FBgn0053288) |
| [CG3344](http://flybase.org/cgi-bin/fbidq.html?FBgn0035154) | - | [CG3344](http://flybase.org/cgi-bin/fbidq.html?FBgn0035154) |
| [CG33453](http://flybase.org/cgi-bin/fbidq.html?FBgn0053453) | - | [CG33453](http://flybase.org/cgi-bin/fbidq.html?FBgn0053453) |
| [CG33475](http://flybase.org/cgi-bin/fbidq.html?FBgn0053475) | - | [CG33475](http://flybase.org/cgi-bin/fbidq.html?FBgn0053475) |
| [CG3397](http://flybase.org/cgi-bin/fbidq.html?FBgn0037975) | - | [CG3397](http://flybase.org/cgi-bin/fbidq.html?FBgn0037975) |
| [CG3446](http://flybase.org/cgi-bin/fbidq.html?FBgn0029868) | - | [CG3446](http://flybase.org/cgi-bin/fbidq.html?FBgn0029868) |
| [CG3458](http://flybase.org/cgi-bin/fbidq.html?FBgn0026015) | Topoisomerase 3beta | [Top3beta](http://flybase.org/cgi-bin/fbidq.html?FBgn0026015) |
| [CG3497](http://flybase.org/cgi-bin/fbidq.html?FBgn0004837) | Suppressor of Hairless | [Su(H)](http://flybase.org/cgi-bin/fbidq.html?FBgn0004837) |
| [CG3510](http://flybase.org/cgi-bin/fbidq.html?FBgn0000405) | Cyclin B | [CycB](http://flybase.org/cgi-bin/fbidq.html?FBgn0000405) |
| [CG3542](http://flybase.org/cgi-bin/fbidq.html?FBgn0031492) | - | [CG3542](http://flybase.org/cgi-bin/fbidq.html?FBgn0031492) |
| [CG3629](http://flybase.org/cgi-bin/fbidq.html?FBgn0000157) | Distal-less | [Dll](http://flybase.org/cgi-bin/fbidq.html?FBgn0000157) |
| [CG3630](http://flybase.org/cgi-bin/fbidq.html?FBgn0023540) | - | [CG3630](http://flybase.org/cgi-bin/fbidq.html?FBgn0023540) |
| [CG3640](http://flybase.org/cgi-bin/fbidq.html?FBgn0035042) | - | [CG3640](http://flybase.org/cgi-bin/fbidq.html?FBgn0035042) |
| [CG3649](http://flybase.org/cgi-bin/fbidq.html?FBgn0034785) | - | [CG3649](http://flybase.org/cgi-bin/fbidq.html?FBgn0034785) |
| [CG3650](http://flybase.org/cgi-bin/fbidq.html?FBgn0035070) | - | [CG3650](http://flybase.org/cgi-bin/fbidq.html?FBgn0035070) |
| [CG3669](http://flybase.org/cgi-bin/fbidq.html?FBgn0039838) | - | [CG3669](http://flybase.org/cgi-bin/fbidq.html?FBgn0039838) |
| [CG3699](http://flybase.org/cgi-bin/fbidq.html?FBgn0040349) | - | [CG3699](http://flybase.org/cgi-bin/fbidq.html?FBgn0040349) |
| [CG3704](http://flybase.org/cgi-bin/fbidq.html?FBgn0040346) | - | [CG3704](http://flybase.org/cgi-bin/fbidq.html?FBgn0040346) |
| [CG3743](http://flybase.org/cgi-bin/fbidq.html?FBgn0040305) | Metal response element-binding Transcription Factor-1 | [MTF-1](http://flybase.org/cgi-bin/fbidq.html?FBgn0040305) |
| [CG3758](http://flybase.org/cgi-bin/fbidq.html?FBgn0001981) | escargot | [esg](http://flybase.org/cgi-bin/fbidq.html?FBgn0001981) |
| [CG3766](http://flybase.org/cgi-bin/fbidq.html?FBgn0011232) | scattered | [scat](http://flybase.org/cgi-bin/fbidq.html?FBgn0011232) |
| [CG3767](http://flybase.org/cgi-bin/fbidq.html?FBgn0028424) | Juvenile hormone-inducible protein 26 | [JhI-26](http://flybase.org/cgi-bin/fbidq.html?FBgn0028424) |
| [CG3801](http://flybase.org/cgi-bin/fbidq.html?FBgn0015586) | Accessory gland-specific peptide 76A | [Acp76A](http://flybase.org/cgi-bin/fbidq.html?FBgn0015586) |
| [CG3822](http://flybase.org/cgi-bin/fbidq.html?FBgn0038837) | - | [CG3822](http://flybase.org/cgi-bin/fbidq.html?FBgn0038837) |
| [CG3842](http://flybase.org/cgi-bin/fbidq.html?FBgn0029866) | - | [CG3842](http://flybase.org/cgi-bin/fbidq.html?FBgn0029866) |
| [CG3868](http://flybase.org/cgi-bin/fbidq.html?FBgn0036422) | - | [CG3868](http://flybase.org/cgi-bin/fbidq.html?FBgn0036422) |
| [CG3902](http://flybase.org/cgi-bin/fbidq.html?FBgn0036824) | - | [CG3902](http://flybase.org/cgi-bin/fbidq.html?FBgn0036824) |
| [CG3910](http://flybase.org/cgi-bin/fbidq.html?FBgn0037778) | mitochondrial transcription factor B2 | [mtTFB2](http://flybase.org/cgi-bin/fbidq.html?FBgn0037778) |
| [CG3971](http://flybase.org/cgi-bin/fbidq.html?FBgn0260960) | Baldspot | [Baldspot](http://flybase.org/cgi-bin/fbidq.html?FBgn0260960) |
| [CG40032](http://flybase.org/cgi-bin/fbidq.html?FBgn0058032) | - | [CG40032](http://flybase.org/cgi-bin/fbidq.html?FBgn0058032) |
| [CG40080](http://flybase.org/cgi-bin/fbidq.html?FBgn0046706) | Haspin | [Haspin](http://flybase.org/cgi-bin/fbidq.html?FBgn0046706) |
| [CG40096](http://flybase.org/cgi-bin/fbidq.html?FBgn0058096) | - | [CG40096](http://flybase.org/cgi-bin/fbidq.html?FBgn0058096) |
| [CG40156](http://flybase.org/cgi-bin/fbidq.html?FBgn0058156) | - | [CG40156](http://flybase.org/cgi-bin/fbidq.html?FBgn0058156) |
| [CG40188](http://flybase.org/cgi-bin/fbidq.html?FBgn0058188) | - | [CG40188](http://flybase.org/cgi-bin/fbidq.html?FBgn0058188) |
| [CG40251](http://flybase.org/cgi-bin/fbidq.html?FBgn0058251) | - | [CG40251](http://flybase.org/cgi-bin/fbidq.html?FBgn0058251) |
| [CG40303](http://flybase.org/cgi-bin/fbidq.html?FBgn0058303) | - | [CG40303](http://flybase.org/cgi-bin/fbidq.html?FBgn0058303) |
| [CG40334](http://flybase.org/cgi-bin/fbidq.html?FBgn0085586) | - | [CG41265](http://flybase.org/cgi-bin/fbidq.html?FBgn0085586) |
| [CG40370](http://flybase.org/cgi-bin/fbidq.html?FBgn0058370) | - | [CG40370](http://flybase.org/cgi-bin/fbidq.html?FBgn0058370) |
| [CG4063](http://flybase.org/cgi-bin/fbidq.html?FBgn0023444) | ebi | [ebi](http://flybase.org/cgi-bin/fbidq.html?FBgn0023444) |
| [CG41040](http://flybase.org/cgi-bin/fbidq.html?FBgn0069967) | - | [CG41040](http://flybase.org/cgi-bin/fbidq.html?FBgn0069967) |
| [CG41063](http://flybase.org/cgi-bin/fbidq.html?FBgn0069956) | - | [CG41063](http://flybase.org/cgi-bin/fbidq.html?FBgn0069956) |
| [CG41106](http://flybase.org/cgi-bin/fbidq.html?FBgn0069938) | - | [CG41106](http://flybase.org/cgi-bin/fbidq.html?FBgn0069938) |
| [CG4147](http://flybase.org/cgi-bin/fbidq.html?FBgn0001218) | Heat shock protein cognate 3 | [Hsc70-3](http://flybase.org/cgi-bin/fbidq.html?FBgn0001218) |
| [CG4169](http://flybase.org/cgi-bin/fbidq.html?FBgn0250814) | - | [CG4169](http://flybase.org/cgi-bin/fbidq.html?FBgn0250814) |
| [CG4206](http://flybase.org/cgi-bin/fbidq.html?FBgn0024332) | Minichromosome maintenance 3 | [Mcm3](http://flybase.org/cgi-bin/fbidq.html?FBgn0024332) |
| [CG4237](http://flybase.org/cgi-bin/fbidq.html?FBgn0020655) | GTPase-activating protein 69C | [Gap69C](http://flybase.org/cgi-bin/fbidq.html?FBgn0020655) |
| [CG4297](http://flybase.org/cgi-bin/fbidq.html?FBgn0031258) | - | [CG4297](http://flybase.org/cgi-bin/fbidq.html?FBgn0031258) |
| [CG4312](http://flybase.org/cgi-bin/fbidq.html?FBgn0002869) | Metallothionein B | [MtnB](http://flybase.org/cgi-bin/fbidq.html?FBgn0002869) |
| [CG4370](http://flybase.org/cgi-bin/fbidq.html?FBgn0039081) | Inwardly rectifying potassium channel 2 | [Irk2](http://flybase.org/cgi-bin/fbidq.html?FBgn0039081) |
| [CG4371](http://flybase.org/cgi-bin/fbidq.html?FBgn0010043) | Glutathione S transferase D7 | [GstD7](http://flybase.org/cgi-bin/fbidq.html?FBgn0010043) |
| [CG4421](http://flybase.org/cgi-bin/fbidq.html?FBgn0010044) | Glutathione S transferase D8 | [GstD8](http://flybase.org/cgi-bin/fbidq.html?FBgn0010044) |
| [CG4422](http://flybase.org/cgi-bin/fbidq.html?FBgn0004868) | GDP dissociation inhibitor | [Gdi](http://flybase.org/cgi-bin/fbidq.html?FBgn0004868) |
| [CG4433](http://flybase.org/cgi-bin/fbidq.html?FBgn0038763) | - | [CG4433](http://flybase.org/cgi-bin/fbidq.html?FBgn0038763) |
| [CG4439](http://flybase.org/cgi-bin/fbidq.html?FBgn0034132) | - | [CG4439](http://flybase.org/cgi-bin/fbidq.html?FBgn0034132) |
| [CG4454](http://flybase.org/cgi-bin/fbidq.html?FBgn0032105) | borealin-related | [borr](http://flybase.org/cgi-bin/fbidq.html?FBgn0032105) |
| [CG4467](http://flybase.org/cgi-bin/fbidq.html?FBgn0039064) | - | [CG4467](http://flybase.org/cgi-bin/fbidq.html?FBgn0039064) |
| [CG4472](http://flybase.org/cgi-bin/fbidq.html?FBgn0020416) | Imaginal disc growth factor 1 | [Idgf1](http://flybase.org/cgi-bin/fbidq.html?FBgn0020416) |
| [CG4477](http://flybase.org/cgi-bin/fbidq.html?FBgn0035971) | - | [CG4477](http://flybase.org/cgi-bin/fbidq.html?FBgn0035971) |
| [CG4484](http://flybase.org/cgi-bin/fbidq.html?FBgn0035968) | - | [CG4484](http://flybase.org/cgi-bin/fbidq.html?FBgn0035968) |
| [CG4538](http://flybase.org/cgi-bin/fbidq.html?FBgn0038745) | - | [CG4538](http://flybase.org/cgi-bin/fbidq.html?FBgn0038745) |
| [CG4562](http://flybase.org/cgi-bin/fbidq.html?FBgn0038740) | - | [CG4562](http://flybase.org/cgi-bin/fbidq.html?FBgn0038740) |
| [CG4734](http://flybase.org/cgi-bin/fbidq.html?FBgn0033826) | - | [CG4734](http://flybase.org/cgi-bin/fbidq.html?FBgn0033826) |
| [CG4746](http://flybase.org/cgi-bin/fbidq.html?FBgn0029003) | mab-21 | [mab-21](http://flybase.org/cgi-bin/fbidq.html?FBgn0029003) |
| [CG4749](http://flybase.org/cgi-bin/fbidq.html?FBgn0031308) | - | [CG4749](http://flybase.org/cgi-bin/fbidq.html?FBgn0031308) |
| [CG4779](http://flybase.org/cgi-bin/fbidq.html?FBgn0040211) | homogentisate 1,2-dioxygenase | [hgo](http://flybase.org/cgi-bin/fbidq.html?FBgn0040211) |
| [CG4804](http://flybase.org/cgi-bin/fbidq.html?FBgn0032178) | - | [CG4804](http://flybase.org/cgi-bin/fbidq.html?FBgn0032178) |
| [CG4807](http://flybase.org/cgi-bin/fbidq.html?FBgn0259750) | abrupt | [ab](http://flybase.org/cgi-bin/fbidq.html?FBgn0259750) |
| [CG4857](http://flybase.org/cgi-bin/fbidq.html?FBgn0026083) | - | [CG4857](http://flybase.org/cgi-bin/fbidq.html?FBgn0026083) |
| [CG4905](http://flybase.org/cgi-bin/fbidq.html?FBgn0034135) | Syntrophin-like 2 | [Syn2](http://flybase.org/cgi-bin/fbidq.html?FBgn0034135) |
| [CG4920](http://flybase.org/cgi-bin/fbidq.html?FBgn0000533) | easter | [ea](http://flybase.org/cgi-bin/fbidq.html?FBgn0000533) |
| [CG4928](http://flybase.org/cgi-bin/fbidq.html?FBgn0027556) | - | [CG4928](http://flybase.org/cgi-bin/fbidq.html?FBgn0027556) |
| [CG4951](http://flybase.org/cgi-bin/fbidq.html?FBgn0039563) | - | [CG4951](http://flybase.org/cgi-bin/fbidq.html?FBgn0039563) |
| [CG4963](http://flybase.org/cgi-bin/fbidq.html?FBgn0039561) | mitoferrin | [mfrn](http://flybase.org/cgi-bin/fbidq.html?FBgn0039561) |
| [CG4977](http://flybase.org/cgi-bin/fbidq.html?FBgn0015400) | kekkon-2 | [kek2](http://flybase.org/cgi-bin/fbidq.html?FBgn0015400) |
| [CG4988](http://flybase.org/cgi-bin/fbidq.html?FBgn0032372) | - | [CG4988](http://flybase.org/cgi-bin/fbidq.html?FBgn0032372) |
| [CG4994](http://flybase.org/cgi-bin/fbidq.html?FBgn0026409) | Mitochondrial phosphate carrier protein | [Mpcp](http://flybase.org/cgi-bin/fbidq.html?FBgn0026409) |
| [CG5010](http://flybase.org/cgi-bin/fbidq.html?FBgn0260747) | - | [CG5010](http://flybase.org/cgi-bin/fbidq.html?FBgn0260747) |
| [CG5028](http://flybase.org/cgi-bin/fbidq.html?FBgn0039358) | - | [CG5028](http://flybase.org/cgi-bin/fbidq.html?FBgn0039358) |
| [CG5224](http://flybase.org/cgi-bin/fbidq.html?FBgn0034354) | - | [CG5224](http://flybase.org/cgi-bin/fbidq.html?FBgn0034354) |
| [CG5270](http://flybase.org/cgi-bin/fbidq.html?FBgn0037897) | - | [CG5270](http://flybase.org/cgi-bin/fbidq.html?FBgn0037897) |
| [CG5281](http://flybase.org/cgi-bin/fbidq.html?FBgn0037902) | - | [CG5281](http://flybase.org/cgi-bin/fbidq.html?FBgn0037902) |
| [CG5326](http://flybase.org/cgi-bin/fbidq.html?FBgn0038983) | - | [CG5326](http://flybase.org/cgi-bin/fbidq.html?FBgn0038983) |
| [CG5327](http://flybase.org/cgi-bin/fbidq.html?FBgn0034363) | - | [CG5327](http://flybase.org/cgi-bin/fbidq.html?FBgn0034363) |
| [CG5333](http://flybase.org/cgi-bin/fbidq.html?FBcl0481533) |  |  |
| [CG5337](http://flybase.org/cgi-bin/fbidq.html?FBgn0032249) | - | [CG5337](http://flybase.org/cgi-bin/fbidq.html?FBgn0032249) |
| [CG5399](http://flybase.org/cgi-bin/fbidq.html?FBgn0038353) | - | [CG5399](http://flybase.org/cgi-bin/fbidq.html?FBgn0038353) |
| [CG5445](http://flybase.org/cgi-bin/fbidq.html?FBgn0030838) | - | [CG5445](http://flybase.org/cgi-bin/fbidq.html?FBgn0030838) |
| [CG5493](http://flybase.org/cgi-bin/fbidq.html?FBgn0034364) | - | [CG5493](http://flybase.org/cgi-bin/fbidq.html?FBgn0034364) |
| [CG5521](http://flybase.org/cgi-bin/fbidq.html?FBgn0039466) | - | [CG5521](http://flybase.org/cgi-bin/fbidq.html?FBgn0039466) |
| [CG5539](http://flybase.org/cgi-bin/fbidq.html?FBgn0034907) | - | [CG5539](http://flybase.org/cgi-bin/fbidq.html?FBgn0034907) |
| [CG5545](http://flybase.org/cgi-bin/fbidq.html?FBgn0032651) | Olig family | [Oli](http://flybase.org/cgi-bin/fbidq.html?FBgn0032651) |
| [CG5596](http://flybase.org/cgi-bin/fbidq.html?FBgn0002772) | Myosin alkali light chain 1 | [Mlc1](http://flybase.org/cgi-bin/fbidq.html?FBgn0002772) |
| [CG5638](http://flybase.org/cgi-bin/fbidq.html?FBgn0036260) | Rhodopsin 7 | [Rh7](http://flybase.org/cgi-bin/fbidq.html?FBgn0036260) |
| [CG5670](http://flybase.org/cgi-bin/fbidq.html?FBgn0002921) | Na pump alpha subunit | [Atpalpha](http://flybase.org/cgi-bin/fbidq.html?FBgn0002921) |
| [CG5714](http://flybase.org/cgi-bin/fbidq.html?FBgn0000543) | ecdysoneless | [ecd](http://flybase.org/cgi-bin/fbidq.html?FBgn0000543) |
| [CG5770](http://flybase.org/cgi-bin/fbidq.html?FBgn0034291) | - | [CG5770](http://flybase.org/cgi-bin/fbidq.html?FBgn0034291) |
| [CG5776](http://flybase.org/cgi-bin/fbidq.html?FBgn0032450) | - | [CG5776](http://flybase.org/cgi-bin/fbidq.html?FBgn0032450) |
| [CG5800](http://flybase.org/cgi-bin/fbidq.html?FBgn0030855) | - | [CG5800](http://flybase.org/cgi-bin/fbidq.html?FBgn0030855) |
| [CG5804](http://flybase.org/cgi-bin/fbidq.html?FBgn0035926) | - | [CG5804](http://flybase.org/cgi-bin/fbidq.html?FBgn0035926) |
| [CG5809](http://flybase.org/cgi-bin/fbidq.html?FBgn0025678) | CaBP1 | [CaBP1](http://flybase.org/cgi-bin/fbidq.html?FBgn0025678) |
| [CG5812](http://flybase.org/cgi-bin/fbidq.html?FBgn0029170) | TweedleT | [TwdlT](http://flybase.org/cgi-bin/fbidq.html?FBgn0029170) |
| [CG5814](http://flybase.org/cgi-bin/fbidq.html?FBgn0015625) | Cyclin B3 | [CycB3](http://flybase.org/cgi-bin/fbidq.html?FBgn0015625) |
| [CG5860](http://flybase.org/cgi-bin/fbidq.html?FBgn0038506) | - | [CG5860](http://flybase.org/cgi-bin/fbidq.html?FBgn0038506) |
| [CG5906](http://flybase.org/cgi-bin/fbidq.html?FBgn0036217) | - | [CG5906](http://flybase.org/cgi-bin/fbidq.html?FBgn0036217) |
| [CG5968](http://flybase.org/cgi-bin/fbidq.html?FBgn0032588) | - | [CG5968](http://flybase.org/cgi-bin/fbidq.html?FBgn0032588) |
| [CG6012](http://flybase.org/cgi-bin/fbidq.html?FBgn0032615) | - | [CG6012](http://flybase.org/cgi-bin/fbidq.html?FBgn0032615) |
| [CG6017](http://flybase.org/cgi-bin/fbidq.html?FBgn0259824) | Huntingtin-interacting protein 14 | [Hip14](http://flybase.org/cgi-bin/fbidq.html?FBgn0259824) |
| [CG6038](http://flybase.org/cgi-bin/fbidq.html?FBgn0036198) | - | [CG6038](http://flybase.org/cgi-bin/fbidq.html?FBgn0036198) |
| [CG6059](http://flybase.org/cgi-bin/fbidq.html?FBgn0039491) | - | [CG6059](http://flybase.org/cgi-bin/fbidq.html?FBgn0039491) |
| [CG6081](http://flybase.org/cgi-bin/fbidq.html?FBgn0031688) | Cyp28d2 | [Cyp28d2](http://flybase.org/cgi-bin/fbidq.html?FBgn0031688) |
| [CG6127](http://flybase.org/cgi-bin/fbidq.html?FBgn0004197) | Serrate | [Ser](http://flybase.org/cgi-bin/fbidq.html?FBgn0004197) |
| [CG6203](http://flybase.org/cgi-bin/fbidq.html?FBgn0028734) | Fmr1 | [Fmr1](http://flybase.org/cgi-bin/fbidq.html?FBgn0028734) |
| [CG6290](http://flybase.org/cgi-bin/fbidq.html?FBgn0030921) | - | [CG6290](http://flybase.org/cgi-bin/fbidq.html?FBgn0030921) |
| [CG6294](http://flybase.org/cgi-bin/fbidq.html?FBgn0030640) | - | [CG6294](http://flybase.org/cgi-bin/fbidq.html?FBgn0030640) |
| [CG6401](http://flybase.org/cgi-bin/fbidq.html?FBgn0034270) | - | [CG6401](http://flybase.org/cgi-bin/fbidq.html?FBgn0034270) |
| [CG6403](http://flybase.org/cgi-bin/fbidq.html?FBgn0039453) | - | [CG6403](http://flybase.org/cgi-bin/fbidq.html?FBgn0039453) |
| [CG6451](http://flybase.org/cgi-bin/fbidq.html?FBgn0041161) | bluestreak | [blue](http://flybase.org/cgi-bin/fbidq.html?FBgn0041161) |
| [CG6464](http://flybase.org/cgi-bin/fbidq.html?FBgn0004579) | spalt major | [salm](http://flybase.org/cgi-bin/fbidq.html?FBgn0004579) |
| [CG6465](http://flybase.org/cgi-bin/fbidq.html?FBgn0037818) | - | [CG6465](http://flybase.org/cgi-bin/fbidq.html?FBgn0037818) |
| [CG6502](http://flybase.org/cgi-bin/fbidq.html?FBgn0000629) | Enhancer of zeste | [E(z)](http://flybase.org/cgi-bin/fbidq.html?FBgn0000629) |
| [CG6515](http://flybase.org/cgi-bin/fbidq.html?FBgn0004841) | Tachykinin-like receptor at 86C | [Takr86C](http://flybase.org/cgi-bin/fbidq.html?FBgn0004841) |
| [CG6518](http://flybase.org/cgi-bin/fbidq.html?FBgn0004784) | inactivation no afterpotential C | [inaC](http://flybase.org/cgi-bin/fbidq.html?FBgn0004784) |
| [CG6577](http://flybase.org/cgi-bin/fbidq.html?FBgn0011569) | cannonball | [can](http://flybase.org/cgi-bin/fbidq.html?FBgn0011569) |
| [CG6600](http://flybase.org/cgi-bin/fbidq.html?FBgn0259164) | - | [CG42269](http://flybase.org/cgi-bin/fbidq.html?FBgn0259164) |
| [CG6620](http://flybase.org/cgi-bin/fbidq.html?FBgn0024227) | IplI-aurora-like kinase | [ial](http://flybase.org/cgi-bin/fbidq.html?FBgn0024227) |
| [CG6738](http://flybase.org/cgi-bin/fbidq.html?FBgn0039053) | - | [CG6738](http://flybase.org/cgi-bin/fbidq.html?FBgn0039053) |
| [CG6741](http://flybase.org/cgi-bin/fbidq.html?FBgn0000008) | arc | [a](http://flybase.org/cgi-bin/fbidq.html?FBgn0000008) |
| [CG6752](http://flybase.org/cgi-bin/fbidq.html?FBgn0038296) | - | [CG6752](http://flybase.org/cgi-bin/fbidq.html?FBgn0038296) |
| [CG6759](http://flybase.org/cgi-bin/fbidq.html?FBgn0025781) | cdc16 | [cdc16](http://flybase.org/cgi-bin/fbidq.html?FBgn0025781) |
| [CG6761](http://flybase.org/cgi-bin/fbidq.html?FBgn0036031) | - | [CG6761](http://flybase.org/cgi-bin/fbidq.html?FBgn0036031) |
| [CG6798](http://flybase.org/cgi-bin/fbidq.html?FBgn0004118) | nicotinic Acetylcholine Receptor beta 96A | [nAcRbeta-96A](http://flybase.org/cgi-bin/fbidq.html?FBgn0004118) |
| [CG6850](http://flybase.org/cgi-bin/fbidq.html?FBgn0014075) | UDP-glucose-glycoprotein glucosyltransferase | [Ugt](http://flybase.org/cgi-bin/fbidq.html?FBgn0014075) |
| [CG6948](http://flybase.org/cgi-bin/fbidq.html?FBgn0024814) | Clathrin light chain | [Clc](http://flybase.org/cgi-bin/fbidq.html?FBgn0024814) |
| [CG6977](http://flybase.org/cgi-bin/fbidq.html?FBgn0037963) | Cad87A | [Cad87A](http://flybase.org/cgi-bin/fbidq.html?FBgn0037963) |
| [CG7009](http://flybase.org/cgi-bin/fbidq.html?FBgn0038861) | - | [CG7009](http://flybase.org/cgi-bin/fbidq.html?FBgn0038861) |
| [CG7023](http://flybase.org/cgi-bin/fbidq.html?FBgn0039025) | - | [CG7023](http://flybase.org/cgi-bin/fbidq.html?FBgn0039025) |
| [CG7091](http://flybase.org/cgi-bin/fbidq.html?FBgn0038099) | - | [CG7091](http://flybase.org/cgi-bin/fbidq.html?FBgn0038099) |
| [CG7128](http://flybase.org/cgi-bin/fbidq.html?FBgn0022724) | TBP-associated factor 8 | [Taf8](http://flybase.org/cgi-bin/fbidq.html?FBgn0022724) |
| [CG7134](http://flybase.org/cgi-bin/fbidq.html?FBgn0031952) | cdc14 | [cdc14](http://flybase.org/cgi-bin/fbidq.html?FBgn0031952) |
| [CG7206](http://flybase.org/cgi-bin/fbidq.html?FBgn0030892) | - | [CG7206](http://flybase.org/cgi-bin/fbidq.html?FBgn0030892) |
| [CG7236](http://flybase.org/cgi-bin/fbidq.html?FBgn0031730) | - | [CG7236](http://flybase.org/cgi-bin/fbidq.html?FBgn0031730) |
| [CG7285](http://flybase.org/cgi-bin/fbidq.html?FBgn0036790) | allatostatin C receptor 1 | [star1](http://flybase.org/cgi-bin/fbidq.html?FBgn0036790) |
| [CG7295](http://flybase.org/cgi-bin/fbidq.html?FBgn0031372) | - | [CG7295](http://flybase.org/cgi-bin/fbidq.html?FBgn0031372) |
| [CG7299](http://flybase.org/cgi-bin/fbidq.html?FBgn0032282) | - | [CG7299](http://flybase.org/cgi-bin/fbidq.html?FBgn0032282) |
| [CG7330](http://flybase.org/cgi-bin/fbidq.html?FBgn0036780) | - | [CG7330](http://flybase.org/cgi-bin/fbidq.html?FBgn0036780) |
| [CG7363](http://flybase.org/cgi-bin/fbidq.html?FBgn0032269) | world cup | [w-cup](http://flybase.org/cgi-bin/fbidq.html?FBgn0032269) |
| [CG7364](http://flybase.org/cgi-bin/fbidq.html?FBgn0028541) | - | [TM9SF4](http://flybase.org/cgi-bin/fbidq.html?FBgn0028541) |
| [CG7365](http://flybase.org/cgi-bin/fbidq.html?FBgn0036939) | - | [CG7365](http://flybase.org/cgi-bin/fbidq.html?FBgn0036939) |
| [CG7398](http://flybase.org/cgi-bin/fbidq.html?FBgn0024921) | Transportin | [Trn](http://flybase.org/cgi-bin/fbidq.html?FBgn0024921) |
| [CG7404](http://flybase.org/cgi-bin/fbidq.html?FBgn0035849) | estrogen-related receptor | [ERR](http://flybase.org/cgi-bin/fbidq.html?FBgn0035849) |
| [CG7462](http://flybase.org/cgi-bin/fbidq.html?FBgn0085445) | Ank2 | [Ank2](http://flybase.org/cgi-bin/fbidq.html?FBgn0085445) |
| [CG7465](http://flybase.org/cgi-bin/fbidq.html?FBgn0035551) | - | [CG7465](http://flybase.org/cgi-bin/fbidq.html?FBgn0035551) |
| [CG7467](http://flybase.org/cgi-bin/fbidq.html?FBgn0003013) | osa | [osa](http://flybase.org/cgi-bin/fbidq.html?FBgn0003013) |
| [CG7470](http://flybase.org/cgi-bin/fbidq.html?FBgn0037146) | - | [CG7470](http://flybase.org/cgi-bin/fbidq.html?FBgn0037146) |
| [CG7532](http://flybase.org/cgi-bin/fbidq.html?FBgn0028915) | - | [CG7532](http://flybase.org/cgi-bin/fbidq.html?FBgn0028915) |
| [CG7546](http://flybase.org/cgi-bin/fbidq.html?FBgn0035793) | - | [CG7546](http://flybase.org/cgi-bin/fbidq.html?FBgn0035793) |
| [CG7568](http://flybase.org/cgi-bin/fbidq.html?FBgn0039673) | - | [CG7568](http://flybase.org/cgi-bin/fbidq.html?FBgn0039673) |
| [CG7586](http://flybase.org/cgi-bin/fbidq.html?FBgn0020240) | Macroglobulin complement-related | [Mcr](http://flybase.org/cgi-bin/fbidq.html?FBgn0020240) |
| [CG7602](http://flybase.org/cgi-bin/fbidq.html?FBgn0037554) | DNApol-iota | [DNApol-iota](http://flybase.org/cgi-bin/fbidq.html?FBgn0037554) |
| [CG7644](http://flybase.org/cgi-bin/fbidq.html?FBgn0028645) | beaten path Ib | [beat-Ib](http://flybase.org/cgi-bin/fbidq.html?FBgn0028645) |
| [CG7659](http://flybase.org/cgi-bin/fbidq.html?FBgn0015550) | target of Poxn | [tap](http://flybase.org/cgi-bin/fbidq.html?FBgn0015550) |
| [CG7675](http://flybase.org/cgi-bin/fbidq.html?FBgn0038610) | - | [CG7675](http://flybase.org/cgi-bin/fbidq.html?FBgn0038610) |
| [CG7708](http://flybase.org/cgi-bin/fbidq.html?FBgn0038641) | - | [CG7708](http://flybase.org/cgi-bin/fbidq.html?FBgn0038641) |
| [CG7712](http://flybase.org/cgi-bin/fbidq.html?FBgn0033570) | - | [CG7712](http://flybase.org/cgi-bin/fbidq.html?FBgn0033570) |
| [CG7714](http://flybase.org/cgi-bin/fbidq.html?FBgn0038645) | - | [CG7714](http://flybase.org/cgi-bin/fbidq.html?FBgn0038645) |
| [CG7715](http://flybase.org/cgi-bin/fbidq.html?FBgn0038646) | - | [CG7715](http://flybase.org/cgi-bin/fbidq.html?FBgn0038646) |
| [CG7719](http://flybase.org/cgi-bin/fbidq.html?FBgn0260399) | greatwall | [gwl](http://flybase.org/cgi-bin/fbidq.html?FBgn0260399) |
| [CG7724](http://flybase.org/cgi-bin/fbidq.html?FBgn0036698) | - | [CG7724](http://flybase.org/cgi-bin/fbidq.html?FBgn0036698) |
| [CG7758](http://flybase.org/cgi-bin/fbidq.html?FBgn0027945) | pumpless | [ppl](http://flybase.org/cgi-bin/fbidq.html?FBgn0027945) |
| [CG7772](http://flybase.org/cgi-bin/fbidq.html?FBgn0030883) | - | [CG7772](http://flybase.org/cgi-bin/fbidq.html?FBgn0030883) |
| [CG7833](http://flybase.org/cgi-bin/fbidq.html?FBgn0015271) | Origin recognition complex subunit 5 | [Orc5](http://flybase.org/cgi-bin/fbidq.html?FBgn0015271) |
| [CG7851](http://flybase.org/cgi-bin/fbidq.html?FBgn0032013) | Sarcoglycan alpha | [Scgalpha](http://flybase.org/cgi-bin/fbidq.html?FBgn0032013) |
| [CG7882](http://flybase.org/cgi-bin/fbidq.html?FBgn0033047) | - | [CG7882](http://flybase.org/cgi-bin/fbidq.html?FBgn0033047) |
| [CG7957](http://flybase.org/cgi-bin/fbidq.html?FBgn0038578) | Mediator complex subunit 17 | [MED17](http://flybase.org/cgi-bin/fbidq.html?FBgn0038578) |
| [CG8023](http://flybase.org/cgi-bin/fbidq.html?FBgn0035860) | eIF4E-3 | [eIF4E-3](http://flybase.org/cgi-bin/fbidq.html?FBgn0035860) |
| [CG8046](http://flybase.org/cgi-bin/fbidq.html?FBgn0033388) | - | [CG8046](http://flybase.org/cgi-bin/fbidq.html?FBgn0033388) |
| [CG8057](http://flybase.org/cgi-bin/fbidq.html?FBgn0260972) | alicorn | [alc](http://flybase.org/cgi-bin/fbidq.html?FBgn0260972) |
| [CG8066](http://flybase.org/cgi-bin/fbidq.html?FBgn0038243) | - | [CG8066](http://flybase.org/cgi-bin/fbidq.html?FBgn0038243) |
| [CG8092](http://flybase.org/cgi-bin/fbidq.html?FBgn0033998) | relative of woc | [row](http://flybase.org/cgi-bin/fbidq.html?FBgn0033998) |
| [CG8116](http://flybase.org/cgi-bin/fbidq.html?FBgn0037614) | - | [CG8116](http://flybase.org/cgi-bin/fbidq.html?FBgn0037614) |
| [CG8147](http://flybase.org/cgi-bin/fbidq.html?FBgn0043791) | - | [CG8147](http://flybase.org/cgi-bin/fbidq.html?FBgn0043791) |
| [CG8178](http://flybase.org/cgi-bin/fbidq.html?FBgn0024319) | Nach | [Nach](http://flybase.org/cgi-bin/fbidq.html?FBgn0024319) |
| [CG8193](http://flybase.org/cgi-bin/fbidq.html?FBgn0033367) | - | [CG8193](http://flybase.org/cgi-bin/fbidq.html?FBgn0033367) |
| [CG8194](http://flybase.org/cgi-bin/fbidq.html?FBgn0010406) | Ribonuclease X25 | [RNaseX25](http://flybase.org/cgi-bin/fbidq.html?FBgn0010406) |
| [CG8196](http://flybase.org/cgi-bin/fbidq.html?FBgn0033366) | Ance-4 | [Ance-4](http://flybase.org/cgi-bin/fbidq.html?FBgn0033366) |
| [CG8213](http://flybase.org/cgi-bin/fbidq.html?FBgn0033359) | - | [CG8213](http://flybase.org/cgi-bin/fbidq.html?FBgn0033359) |
| [CG8253](http://flybase.org/cgi-bin/fbidq.html?FBgn0034046) | tungus | [tun](http://flybase.org/cgi-bin/fbidq.html?FBgn0034046) |
| [CG8254](http://flybase.org/cgi-bin/fbidq.html?FBgn0041156) | extra-extra | [exex](http://flybase.org/cgi-bin/fbidq.html?FBgn0041156) |
| [CG8297](http://flybase.org/cgi-bin/fbidq.html?FBgn0034050) | - | [CG8297](http://flybase.org/cgi-bin/fbidq.html?FBgn0034050) |
| [CG8369](http://flybase.org/cgi-bin/fbidq.html?FBgn0040532) | - | [CG8369](http://flybase.org/cgi-bin/fbidq.html?FBgn0040532) |
| [CG8374](http://flybase.org/cgi-bin/fbidq.html?FBgn0016792) | dalmatian | [dmt](http://flybase.org/cgi-bin/fbidq.html?FBgn0016792) |
| [CG8384](http://flybase.org/cgi-bin/fbidq.html?FBgn0001139) | groucho | [gro](http://flybase.org/cgi-bin/fbidq.html?FBgn0001139) |
| [CG8399](http://flybase.org/cgi-bin/fbidq.html?FBgn0034067) | - | [CG8399](http://flybase.org/cgi-bin/fbidq.html?FBgn0034067) |
| [CG8445](http://flybase.org/cgi-bin/fbidq.html?FBgn0034088) | - | [CG8445](http://flybase.org/cgi-bin/fbidq.html?FBgn0034088) |
| [CG8540](http://flybase.org/cgi-bin/fbidq.html?FBgn0035790) | Cyp316a1 | [Cyp316a1](http://flybase.org/cgi-bin/fbidq.html?FBgn0035790) |
| [CG8571](http://flybase.org/cgi-bin/fbidq.html?FBgn0016983) | smallminded | [smid](http://flybase.org/cgi-bin/fbidq.html?FBgn0016983) |
| [CG8577](http://flybase.org/cgi-bin/fbidq.html?FBgn0033327) | PGRP-SC1b | [PGRP-SC1b](http://flybase.org/cgi-bin/fbidq.html?FBgn0033327) |
| [CG8603](http://flybase.org/cgi-bin/fbidq.html?FBgn0085408) | Shroom | [Shroom](http://flybase.org/cgi-bin/fbidq.html?FBgn0085408) |
| [CG8610](http://flybase.org/cgi-bin/fbidq.html?FBgn0012058) | Cdc27 | [Cdc27](http://flybase.org/cgi-bin/fbidq.html?FBgn0012058) |
| [CG8629](http://flybase.org/cgi-bin/fbidq.html?FBgn0035742) | - | [CG8629](http://flybase.org/cgi-bin/fbidq.html?FBgn0035742) |
| [CG8696](http://flybase.org/cgi-bin/fbidq.html?FBgn0002570) | Larval visceral protein H | [LvpH](http://flybase.org/cgi-bin/fbidq.html?FBgn0002570) |
| [CG8740](http://flybase.org/cgi-bin/fbidq.html?FBgn0027585) | - | [CG8740](http://flybase.org/cgi-bin/fbidq.html?FBgn0027585) |
| [CG8873](http://flybase.org/cgi-bin/fbidq.html?FBgn0031652) | jetlag | [jet](http://flybase.org/cgi-bin/fbidq.html?FBgn0031652) |
| [CG8889](http://flybase.org/cgi-bin/fbidq.html?FBgn0259985) | - | [CG8889](http://flybase.org/cgi-bin/fbidq.html?FBgn0259985) |
| [CG8896](http://flybase.org/cgi-bin/fbidq.html?FBgn0004364) | 18 wheeler | [18w](http://flybase.org/cgi-bin/fbidq.html?FBgn0004364) |
| [CG8912](http://flybase.org/cgi-bin/fbidq.html?FBgn0014870) | P-element somatic inhibitor | [Psi](http://flybase.org/cgi-bin/fbidq.html?FBgn0014870) |
| [CG8931](http://flybase.org/cgi-bin/fbidq.html?FBgn0030717) | - | [CG8931](http://flybase.org/cgi-bin/fbidq.html?FBgn0030717) |
| [CG8959](http://flybase.org/cgi-bin/fbidq.html?FBgn0030691) | - | [CG8959](http://flybase.org/cgi-bin/fbidq.html?FBgn0030691) |
| [CG8962](http://flybase.org/cgi-bin/fbidq.html?FBgn0025809) | Platelet-activating factor acetylhydrolase alpha | [Paf-AHalpha](http://flybase.org/cgi-bin/fbidq.html?FBgn0025809) |
| [CG8988](http://flybase.org/cgi-bin/fbidq.html?FBgn0033656) | S2P | [S2P](http://flybase.org/cgi-bin/fbidq.html?FBgn0033656) |
| [CG8989](http://flybase.org/cgi-bin/fbidq.html?FBgn0004828) | Histone H3.3B | [His3.3B](http://flybase.org/cgi-bin/fbidq.html?FBgn0004828) |
| [CG9030](http://flybase.org/cgi-bin/fbidq.html?FBgn0030599) | - | [CG9030](http://flybase.org/cgi-bin/fbidq.html?FBgn0030599) |
| [CG9057](http://flybase.org/cgi-bin/fbidq.html?FBgn0030608) | Lipid storage droplet-2 | [Lsd-2](http://flybase.org/cgi-bin/fbidq.html?FBgn0030608) |
| [CG9129](http://flybase.org/cgi-bin/fbidq.html?FBgn0035196) | - | [CG9129](http://flybase.org/cgi-bin/fbidq.html?FBgn0035196) |
| [CG9144](http://flybase.org/cgi-bin/fbidq.html?FBgn0031773) | - | [Fbw5](http://flybase.org/cgi-bin/fbidq.html?FBgn0031773) |
| [CG9259](http://flybase.org/cgi-bin/fbidq.html?FBgn0032913) | - | [CG9259](http://flybase.org/cgi-bin/fbidq.html?FBgn0032913) |
| [CG9270](http://flybase.org/cgi-bin/fbidq.html?FBgn0032908) | - | [CG9270](http://flybase.org/cgi-bin/fbidq.html?FBgn0032908) |
| [CG9288](http://flybase.org/cgi-bin/fbidq.html?FBgn0260464) | - | [CG9288](http://flybase.org/cgi-bin/fbidq.html?FBgn0260464) |
| [CG9299](http://flybase.org/cgi-bin/fbidq.html?FBgn0036881) | Cuticular protein 76Bd | [Cpr76Bd](http://flybase.org/cgi-bin/fbidq.html?FBgn0036881) |
| [CG9300](http://flybase.org/cgi-bin/fbidq.html?FBgn0036886) | - | [CG9300](http://flybase.org/cgi-bin/fbidq.html?FBgn0036886) |
| [CG9310](http://flybase.org/cgi-bin/fbidq.html?FBgn0004914) | Hepatocyte nuclear factor 4 | [Hnf4](http://flybase.org/cgi-bin/fbidq.html?FBgn0004914) |
| [CG9330](http://flybase.org/cgi-bin/fbidq.html?FBgn0036888) | - | [CG9330](http://flybase.org/cgi-bin/fbidq.html?FBgn0036888) |
| [CG9354](http://flybase.org/cgi-bin/fbidq.html?FBgn0037686) | Ribosomal protein L34b | [RpL34b](http://flybase.org/cgi-bin/fbidq.html?FBgn0037686) |
| [CG9384](http://flybase.org/cgi-bin/fbidq.html?FBgn0036446) | - | [CG9384](http://flybase.org/cgi-bin/fbidq.html?FBgn0036446) |
| [CG9394](http://flybase.org/cgi-bin/fbidq.html?FBgn0034588) | - | [CG9394](http://flybase.org/cgi-bin/fbidq.html?FBgn0034588) |
| [CG9453](http://flybase.org/cgi-bin/fbidq.html?FBgn0028985) | Serine protease inhibitor 4 | [Spn4](http://flybase.org/cgi-bin/fbidq.html?FBgn0028985) |
| [CG9485](http://flybase.org/cgi-bin/fbidq.html?FBgn0034618) | - | [CG9485](http://flybase.org/cgi-bin/fbidq.html?FBgn0034618) |
| [CG9528](http://flybase.org/cgi-bin/fbidq.html?FBgn0031814) | real-time | [retm](http://flybase.org/cgi-bin/fbidq.html?FBgn0031814) |
| [CG9540](http://flybase.org/cgi-bin/fbidq.html?FBgn0020508) | Antigen 5-related 2 | [Ag5r2](http://flybase.org/cgi-bin/fbidq.html?FBgn0020508) |
| [CG9655](http://flybase.org/cgi-bin/fbidq.html?FBgn0026630) | nessy | [nes](http://flybase.org/cgi-bin/fbidq.html?FBgn0026630) |
| [CG9681](http://flybase.org/cgi-bin/fbidq.html?FBgn0043578) | PGRP-SB1 | [PGRP-SB1](http://flybase.org/cgi-bin/fbidq.html?FBgn0043578) |
| [CG9699](http://flybase.org/cgi-bin/fbidq.html?FBgn0259923) | Septin 4 | [Sep4](http://flybase.org/cgi-bin/fbidq.html?FBgn0259923) |
| [CG9742](http://flybase.org/cgi-bin/fbidq.html?FBgn0030765) | Small ribonucleoprotein G | [SmG](http://flybase.org/cgi-bin/fbidq.html?FBgn0030765) |
| [CG9755](http://flybase.org/cgi-bin/fbidq.html?FBgn0003165) | pumilio | [pum](http://flybase.org/cgi-bin/fbidq.html?FBgn0003165) |
| [CG9793](http://flybase.org/cgi-bin/fbidq.html?FBgn0037620) | - | [CG9793](http://flybase.org/cgi-bin/fbidq.html?FBgn0037620) |
| [CG9815](http://flybase.org/cgi-bin/fbidq.html?FBgn0034861) | - | [CG9815](http://flybase.org/cgi-bin/fbidq.html?FBgn0034861) |
| [CG9842](http://flybase.org/cgi-bin/fbidq.html?FBgn0011826) | Protein phosphatase 2B at 14D | [Pp2B-14D](http://flybase.org/cgi-bin/fbidq.html?FBgn0011826) |
| [CG9981](http://flybase.org/cgi-bin/fbidq.html?FBgn0030746) | - | [CG9981](http://flybase.org/cgi-bin/fbidq.html?FBgn0030746) |
| [CG9995](http://flybase.org/cgi-bin/fbidq.html?FBgn0027655) | huntingtin | [htt](http://flybase.org/cgi-bin/fbidq.html?FBgn0027655) |
| [CR30087](http://flybase.org/cgi-bin/fbidq.html?FBgn0050087) | - | [CG30087](http://flybase.org/cgi-bin/fbidq.html?FBgn0050087) |
| [CR30241](http://flybase.org/cgi-bin/fbidq.html?FBgn0050241) | transfer RNA:CR30241 | [tRNA:CR30241](http://flybase.org/cgi-bin/fbidq.html?FBgn0050241) |
| [CR30249](http://flybase.org/cgi-bin/fbidq.html?FBgn0050249) | transfer RNA:CR30249 | [tRNA:CR30249](http://flybase.org/cgi-bin/fbidq.html?FBgn0050249) |
| [CR31485](http://flybase.org/cgi-bin/fbidq.html?FBgn0051485) | transfer RNA:CR31485:pseudogene | [tRNA:CR31485:Psi](http://flybase.org/cgi-bin/fbidq.html?FBgn0051485) |
| [CR31511](http://flybase.org/cgi-bin/fbidq.html?FBgn0027934) | alpha-Est4aPsi | [alpha-Est4aPsi](http://flybase.org/cgi-bin/fbidq.html?FBgn0027934) |
| [CR31850](http://flybase.org/cgi-bin/fbidq.html?FBgn0004191) | small nuclear RNA U2 at 34ABa | [snRNA:U2:34ABa](http://flybase.org/cgi-bin/fbidq.html?FBgn0004191) |
| [CR31863](http://flybase.org/cgi-bin/fbidq.html?FBgn0041606) | bereft | [bft](http://flybase.org/cgi-bin/fbidq.html?FBgn0041606) |
| [CR32525](http://flybase.org/cgi-bin/fbidq.html?FBgn0052525) | transfer RNA:CR32525 | [tRNA:CR32525](http://flybase.org/cgi-bin/fbidq.html?FBgn0052525) |
| [CR33363](http://flybase.org/cgi-bin/fbidq.html?FBgn0053363) | 5SrRNA-Psi:CR33363 | [5SrRNA-Psi:CR33363](http://flybase.org/cgi-bin/fbidq.html?FBgn0053363) |
