# Supplementary material for: Chronic activation of the epithelial immune system of the fruit fly's salivary glands has a negative effect on organismal growth and induces a peculiar set of target genes
Source: BMC Genomics. 2010 Apr 26;11:265. doi: 10.1186/1471-2164-11-265 (PMC2874812; doi:10.1186/1471-2164-11-265)
Supplement: Additional file 5 — Drosophila Salivary glands genes upregulated following IMD-pathway activation - ribonucleoprotein complex. This file contains a list of those genes whose expression in the salivary glands is upregulated significantly. It comprises only those genes with an annotation as ribonucleoprotein complex. [file 1471-2164-11-265-S5.DOC]

*Drosophila Salivary glands genes upregulated following IMD-pathway activation*

Drosophila Salivary glands IMD upregulated genes GO cellular component level 6

[ribonucleoprotein complex (GO:0030529)](http://amigo.geneontology.org/cgi-bin/amigo/go.cgi?view=assoc&search_constraint=terms&query=GO:0030529)

| **SUBMITTED ID** | **NAME** | **SYMBOL** |
| --- | --- | --- |
| [CG3949](http://flybase.org/cgi-bin/fbidq.html?FBgn0015393) | hoi-polloi | [hoip](http://flybase.org/cgi-bin/fbidq.html?FBgn0015393) |
| [CG6764](http://flybase.org/cgi-bin/fbidq.html?FBgn0037899) | - | [CG6764](http://flybase.org/cgi-bin/fbidq.html?FBgn0037899) |
| [CG6011](http://flybase.org/cgi-bin/fbidq.html?FBgn0027784) | Prp18 | [Prp18](http://flybase.org/cgi-bin/fbidq.html?FBgn0027784) |
| [CG7424](http://flybase.org/cgi-bin/fbidq.html?FBgn0031980) | Ribosomal protein L36A | [RpL36A](http://flybase.org/cgi-bin/fbidq.html?FBgn0031980) |
| [CG3997](http://flybase.org/cgi-bin/fbidq.html?FBgn0023170) | Ribosomal protein L39 | [RpL39](http://flybase.org/cgi-bin/fbidq.html?FBgn0023170) |
| [CG12220](http://flybase.org/cgi-bin/fbidq.html?FBgn0039835) | mitochondrial ribosomal protein L32 | [mRpL32](http://flybase.org/cgi-bin/fbidq.html?FBgn0039835) |
| [CG7925](http://flybase.org/cgi-bin/fbidq.html?FBgn0003714) | technical knockout | [tko](http://flybase.org/cgi-bin/fbidq.html?FBgn0003714) |
| [CG6779](http://flybase.org/cgi-bin/fbidq.html?FBgn0002622) | Ribosomal protein S3 | [RpS3](http://flybase.org/cgi-bin/fbidq.html?FBgn0002622) |
| [CG2998](http://flybase.org/cgi-bin/fbidq.html?FBgn0030136) | Ribosomal protein S28b | [RpS28b](http://flybase.org/cgi-bin/fbidq.html?FBgn0030136) |
| [CG8615](http://flybase.org/cgi-bin/fbidq.html?FBgn0035753) | Ribosomal protein L18 | [RpL18](http://flybase.org/cgi-bin/fbidq.html?FBgn0035753) |
| [CG14641](http://flybase.org/cgi-bin/fbidq.html?FBgn0037220) | - | [CG14641](http://flybase.org/cgi-bin/fbidq.html?FBgn0037220) |
| [CG8922](http://flybase.org/cgi-bin/fbidq.html?FBgn0002590) | Ribosomal protein S5a | [RpS5a](http://flybase.org/cgi-bin/fbidq.html?FBgn0002590) |
| [CG8332](http://flybase.org/cgi-bin/fbidq.html?FBgn0034138) | Ribosomal protein S15 | [RpS15](http://flybase.org/cgi-bin/fbidq.html?FBgn0034138) |
| [CG5258](http://flybase.org/cgi-bin/fbidq.html?FBgn0029148) | NHP2 | [NHP2](http://flybase.org/cgi-bin/fbidq.html?FBgn0029148) |
| [CG2168](http://flybase.org/cgi-bin/fbidq.html?FBgn0017545) | Ribosomal protein S3A | [RpS3A](http://flybase.org/cgi-bin/fbidq.html?FBgn0017545) |
| [CG18591](http://flybase.org/cgi-bin/fbidq.html?FBgn0031962) | - | [CG18591](http://flybase.org/cgi-bin/fbidq.html?FBgn0031962) |
| [CG1527](http://flybase.org/cgi-bin/fbidq.html?FBgn0004404) | Ribosomal protein S14b | [RpS14b](http://flybase.org/cgi-bin/fbidq.html?FBgn0004404) |
| [CG7622](http://flybase.org/cgi-bin/fbidq.html?FBgn0002579) | Ribosomal protein L36 | [RpL36](http://flybase.org/cgi-bin/fbidq.html?FBgn0002579) |
| [CG31450](http://flybase.org/cgi-bin/fbidq.html?FBgn0051450) | mitochondrial ribosomal protein S18A | [mRpS18A](http://flybase.org/cgi-bin/fbidq.html?FBgn0051450) |
| [CG1320](http://flybase.org/cgi-bin/fbidq.html?FBgn0035335) | mitochondrial ribosomal protein L23 | [mRpL23](http://flybase.org/cgi-bin/fbidq.html?FBgn0035335) |
| [CG31184](http://flybase.org/cgi-bin/fbidq.html?FBgn0051184) | - | [LSm3](http://flybase.org/cgi-bin/fbidq.html?FBgn0051184) |
| [CG12324](http://flybase.org/cgi-bin/fbidq.html?FBgn0033555) | Ribosomal protein S15Ab | [RpS15Ab](http://flybase.org/cgi-bin/fbidq.html?FBgn0033555) |
| [CG1249](http://flybase.org/cgi-bin/fbidq.html?FBgn0037434) | snRNP2 | [snRNP2](http://flybase.org/cgi-bin/fbidq.html?FBgn0037434) |
| [CG4247](http://flybase.org/cgi-bin/fbidq.html?FBgn0038307) | mitochondrial ribosomal protein S10 | [mRpS10](http://flybase.org/cgi-bin/fbidq.html?FBgn0038307) |
| [CG17420](http://flybase.org/cgi-bin/fbidq.html?FBgn0028697) | Ribosomal protein L15 | [RpL15](http://flybase.org/cgi-bin/fbidq.html?FBgn0028697) |
| [CG11522](http://flybase.org/cgi-bin/fbidq.html?FBgn0039857) | Ribosomal protein L6 | [RpL6](http://flybase.org/cgi-bin/fbidq.html?FBgn0039857) |
| [CG5920](http://flybase.org/cgi-bin/fbidq.html?FBgn0004867) | string of pearls | [sop](http://flybase.org/cgi-bin/fbidq.html?FBgn0004867) |
| [CG11985](http://flybase.org/cgi-bin/fbidq.html?FBgn0040534) | - | [CG11985](http://flybase.org/cgi-bin/fbidq.html?FBgn0040534) |
| [CG9091](http://flybase.org/cgi-bin/fbidq.html?FBgn0030616) | Ribosomal protein L37a | [RpL37a](http://flybase.org/cgi-bin/fbidq.html?FBgn0030616) |
| [CG8268](http://flybase.org/cgi-bin/fbidq.html?FBgn0035827) | Srp9 | [Srp9](http://flybase.org/cgi-bin/fbidq.html?FBgn0035827) |
| [CG5338](http://flybase.org/cgi-bin/fbidq.html?FBgn0039129) | Ribosomal protein S19b | [RpS19b](http://flybase.org/cgi-bin/fbidq.html?FBgn0039129) |
| [CG5352](http://flybase.org/cgi-bin/fbidq.html?FBgn0010083) | Small ribonucleoprotein particle protein B | [SmB](http://flybase.org/cgi-bin/fbidq.html?FBgn0010083) |
| [CG10423](http://flybase.org/cgi-bin/fbidq.html?FBgn0039300) | Ribosomal protein S27 | [RpS27](http://flybase.org/cgi-bin/fbidq.html?FBgn0039300) |
| [CG6510](http://flybase.org/cgi-bin/fbidq.html?FBgn0010409) | Ribosomal protein L18A | [RpL18A](http://flybase.org/cgi-bin/fbidq.html?FBgn0010409) |
| [CG3314](http://flybase.org/cgi-bin/fbidq.html?FBgn0014026) | Ribosomal protein L7A | [RpL7A](http://flybase.org/cgi-bin/fbidq.html?FBgn0014026) |
| [CG5184](http://flybase.org/cgi-bin/fbidq.html?FBgn0038474) | mitochondrial ribosomal protein S11 | [mRpS11](http://flybase.org/cgi-bin/fbidq.html?FBgn0038474) |

Drosophila Salivary glands IMD upregulated genes GO cellular component level 6

[proteasome complex (sensu Eukaryota) (GO:0000502)](http://amigo.geneontology.org/cgi-bin/amigo/go.cgi?view=assoc&search_constraint=terms&query=GO:0000502)

| **SUBMITTED ID** | **NAME** | **SYMBOL** |
| --- | --- | --- |
| [CG9327](http://flybase.org/cgi-bin/fbidq.html?FBgn0261394) | Proteasome 29kD subunit | [Pros29](http://flybase.org/cgi-bin/fbidq.html?FBgn0261394) |
| [CG18495](http://flybase.org/cgi-bin/fbidq.html?FBgn0026781) | Proteasome alpha1 subunit | [Prosalpha1](http://flybase.org/cgi-bin/fbidq.html?FBgn0026781) |
| [CG11981](http://flybase.org/cgi-bin/fbidq.html?FBgn0026380) | Proteasome beta3 subunit | [Prosbeta3](http://flybase.org/cgi-bin/fbidq.html?FBgn0026380) |
| [CG10230](http://flybase.org/cgi-bin/fbidq.html?FBgn0028691) | Rpn9 | [Rpn9](http://flybase.org/cgi-bin/fbidq.html?FBgn0028691) |
| [CG4097](http://flybase.org/cgi-bin/fbidq.html?FBgn0002284) | Proteasome 26kD subunit | [Pros26](http://flybase.org/cgi-bin/fbidq.html?FBgn0002284) |
| [CG5266](http://flybase.org/cgi-bin/fbidq.html?FBgn0086134) | Proteasome 25kD subunit | [Pros25](http://flybase.org/cgi-bin/fbidq.html?FBgn0086134) |
| [CG1489](http://flybase.org/cgi-bin/fbidq.html?FBgn0020369) | Pros45 | [Pros45](http://flybase.org/cgi-bin/fbidq.html?FBgn0020369) |
| [CG12323](http://flybase.org/cgi-bin/fbidq.html?FBgn0029134) | Proteasome beta5 subunit | [Prosbeta5](http://flybase.org/cgi-bin/fbidq.html?FBgn0029134) |
| [CG30382](http://flybase.org/cgi-bin/fbidq.html?FBgn0050382) | - | [CG30382](http://flybase.org/cgi-bin/fbidq.html?FBgn0050382) |
| [CG16916](http://flybase.org/cgi-bin/fbidq.html?FBgn0028686) | Rpt3 | [Rpt3](http://flybase.org/cgi-bin/fbidq.html?FBgn0028686) |

Drosophila Salivary glands IMD upregulated genes GO molecular function level 4

[cation transporter activity (GO:0008324)](http://amigo.geneontology.org/cgi-bin/amigo/go.cgi?view=assoc&search_constraint=terms&query=GO:0008324)

| **SUBMITTED ID** | **NAME** | **SYMBOL** |
| --- | --- | --- |
| [CG14724](http://flybase.org/cgi-bin/fbidq.html?FBgn0019624) | Cytochrome c oxidase subunit Va | [CoVa](http://flybase.org/cgi-bin/fbidq.html?FBgn0019624) |
| [CG9603](http://flybase.org/cgi-bin/fbidq.html?FBgn0040529) | - | [CG9603](http://flybase.org/cgi-bin/fbidq.html?FBgn0040529) |
| [CG8186](http://flybase.org/cgi-bin/fbidq.html?FBgn0022097) | Vha36 | [Vha36](http://flybase.org/cgi-bin/fbidq.html?FBgn0022097) |
| [CG2968](http://flybase.org/cgi-bin/fbidq.html?FBgn0028342) | lethal (1) G0230 | [l(1)G0230](http://flybase.org/cgi-bin/fbidq.html?FBgn0028342) |
| [CG6030](http://flybase.org/cgi-bin/fbidq.html?FBgn0016120) | ATP synthase, subunit d | [ATPsyn-d](http://flybase.org/cgi-bin/fbidq.html?FBgn0016120) |
| [CG11154](http://flybase.org/cgi-bin/fbidq.html?FBgn0010217) | ATP synthase-beta | [ATPsyn-beta](http://flybase.org/cgi-bin/fbidq.html?FBgn0010217) |
| [CG2934](http://flybase.org/cgi-bin/fbidq.html?FBgn0028665) | Vacuolar H[+]-ATPase C39 subunit | [VhaAC39](http://flybase.org/cgi-bin/fbidq.html?FBgn0028665) |
| [CG3560](http://flybase.org/cgi-bin/fbidq.html?FBgn0030733) | - | [CG3560](http://flybase.org/cgi-bin/fbidq.html?FBgn0030733) |
| [CG11015](http://flybase.org/cgi-bin/fbidq.html?FBgn0031830) | - | [CG11015](http://flybase.org/cgi-bin/fbidq.html?FBgn0031830) |
| [CG11043](http://flybase.org/cgi-bin/fbidq.html?FBgn0031831) | - | [CG11043](http://flybase.org/cgi-bin/fbidq.html?FBgn0031831) |
| [CG7625](http://flybase.org/cgi-bin/fbidq.html?FBgn0028663) | VhaM9.7-2 | [VhaM9.7-2](http://flybase.org/cgi-bin/fbidq.html?FBgn0028663) |

Drosophila Salivary glands IMD upregulated genes

KEGG pathways

[Oxidative phosphorylation (dme00190)](http://www.genome.jp/dbget-bin/www_bget?pathway+dme00190)

| **SUBMITTED ID** | **NAME** | **SYMBOL** |
| --- | --- | --- |
| [CG2014](http://flybase.org/cgi-bin/fbidq.html?FBgn0039669) | - | [CG2014](http://flybase.org/cgi-bin/fbidq.html?FBgn0039669) |
| [CG7625](http://flybase.org/cgi-bin/fbidq.html?FBgn0028663) | VhaM9.7-2 | [VhaM9.7-2](http://flybase.org/cgi-bin/fbidq.html?FBgn0028663) |
| [CG18624](http://flybase.org/cgi-bin/fbidq.html?FBgn0029971) | - | [CG18624](http://flybase.org/cgi-bin/fbidq.html?FBgn0029971) |
| [CG9762](http://flybase.org/cgi-bin/fbidq.html?FBgn0011455) | lethal (3) neo18 | [l(3)neo18](http://flybase.org/cgi-bin/fbidq.html?FBgn0011455) |
| [CG3560](http://flybase.org/cgi-bin/fbidq.html?FBgn0030733) | - | [CG3560](http://flybase.org/cgi-bin/fbidq.html?FBgn0030733) |
| [CG11015](http://flybase.org/cgi-bin/fbidq.html?FBgn0031830) | - | [CG11015](http://flybase.org/cgi-bin/fbidq.html?FBgn0031830) |
| [CG11043](http://flybase.org/cgi-bin/fbidq.html?FBgn0031831) | - | [CG11043](http://flybase.org/cgi-bin/fbidq.html?FBgn0031831) |
| [CG3683](http://flybase.org/cgi-bin/fbidq.html?FBgn0035046) | - | [CG3683](http://flybase.org/cgi-bin/fbidq.html?FBgn0035046) |
| [CG5703](http://flybase.org/cgi-bin/fbidq.html?FBgn0030853) | - | [CG5703](http://flybase.org/cgi-bin/fbidq.html?FBgn0030853) |
| [CG2968](http://flybase.org/cgi-bin/fbidq.html?FBgn0028342) | lethal (1) G0230 | [l(1)G0230](http://flybase.org/cgi-bin/fbidq.html?FBgn0028342) |
| [CG8844](http://flybase.org/cgi-bin/fbidq.html?FBgn0021967) | Pdsw | [Pdsw](http://flybase.org/cgi-bin/fbidq.html?FBgn0021967) |

*Drosophila Salivary glands genes downregulated following IMD-pathway activation*

Drosophila Salivary glands IMD downregulated genes GO molecular function level 4

[peptide receptor activity (GO:0001653)](http://amigo.geneontology.org/cgi-bin/amigo/go.cgi?view=assoc&search_constraint=terms&query=GO:0001653)

| **SUBMITTED ID** | **NAME** | **SYMBOL** |
| --- | --- | --- |
| [CG6515](http://flybase.org/cgi-bin/fbidq.html?FBgn0004841) | Tachykinin-like receptor at 86C | [Takr86C](http://flybase.org/cgi-bin/fbidq.html?FBgn0004841) |
| [CG7285](http://flybase.org/cgi-bin/fbidq.html?FBgn0036790) | allatostatin C receptor 1 | [star1](http://flybase.org/cgi-bin/fbidq.html?FBgn0036790) |
| [CG10823](http://flybase.org/cgi-bin/fbidq.html?FBgn0038880) | SIFamide receptor | [SIFR](http://flybase.org/cgi-bin/fbidq.html?FBgn0038880) |
| [CG13702](http://flybase.org/cgi-bin/fbidq.html?FBgn0036789) | allatostatin C receptor 2 | [AlCR2](http://flybase.org/cgi-bin/fbidq.html?FBgn0036789) |
| [CG11325](http://flybase.org/cgi-bin/fbidq.html?FBgn0025595) | Gonadotropin-releasing hormone receptor | [GRHR](http://flybase.org/cgi-bin/fbidq.html?FBgn0025595) |
| [CG10001](http://flybase.org/cgi-bin/fbidq.html?FBgn0039595) | Allatostatin Receptor 2 | [AR-2](http://flybase.org/cgi-bin/fbidq.html?FBgn0039595) |

Drosophila Salivary glands IMD downregulated genes GO molecular function level 5

[alkali metal ion binding (GO:0031420)](http://amigo.geneontology.org/cgi-bin/amigo/go.cgi?view=assoc&search_constraint=terms&query=GO:0031420)

| **SUBMITTED ID** | **NAME** | **SYMBOL** |
| --- | --- | --- |
| [CG7708](http://flybase.org/cgi-bin/fbidq.html?FBgn0038641) | - | [CG7708](http://flybase.org/cgi-bin/fbidq.html?FBgn0038641) |
| [CG1066](http://flybase.org/cgi-bin/fbidq.html?FBgn0003383) | Shaker cognate b | [Shab](http://flybase.org/cgi-bin/fbidq.html?FBgn0003383) |
| [CG4370](http://flybase.org/cgi-bin/fbidq.html?FBgn0039081) | Inwardly rectifying potassium channel 2 | [Irk2](http://flybase.org/cgi-bin/fbidq.html?FBgn0039081) |
| [CG10952](http://flybase.org/cgi-bin/fbidq.html?FBgn0000535) | ether a go-go | [eag](http://flybase.org/cgi-bin/fbidq.html?FBgn0000535) |
| [CG8178](http://flybase.org/cgi-bin/fbidq.html?FBgn0024319) | Nach | [Nach](http://flybase.org/cgi-bin/fbidq.html?FBgn0024319) |
| [CG5670](http://flybase.org/cgi-bin/fbidq.html?FBgn0002921) | Na pump alpha subunit | [Atpalpha](http://flybase.org/cgi-bin/fbidq.html?FBgn0002921) |
